# Effective Nitrate Electroconversion to Ammonia Using an Entangled Co_3_O_4_/Graphene Nanoribbon Catalyst

**DOI:** 10.1021/acsami.4c18269

**Published:** 2024-12-27

**Authors:** Marciélli K. R. Souza, Eduardo S. F. Cardoso, Leandro M. C. Pinto, Isabela S. C. Crivelli, Clauber D. Rodrigues, Robson S. Souto, Ary T. Rezende-Filho, Marcos R. V. Lanza, Gilberto Maia

**Affiliations:** † Institute of Chemistry, Federal University of Mato Grosso do Sul, Avenida Senador Filinto Muller 1555, Campo Grande, Mato Grosso do Sul 79074-460, Brazil; ‡ São Carlos Institute of Chemistry, University of São Paulo, Avenida Trabalhador São-Carlense 400, São CarlosSão Paulo 13566-590, Brazil; § State University of Mato Grosso do Sul, Rua Rogério Luis Rodrigues s/n, Glória de Dourados, Mato Grosso do Sul 79730-000, Brazil; ∥ Faculty of Engineering, Architecture and Urbanism, and Geography, Federal University of Mato Grosso do Sul, Avenida Costa e Silva, s/n°, Campo Grande, Mato Grosso do Sul 79070-900, Brazil

**Keywords:** Ammonia, nitrate electroreduction, graphene nanoribbon, Co_3_O_4_, entanglement

## Abstract

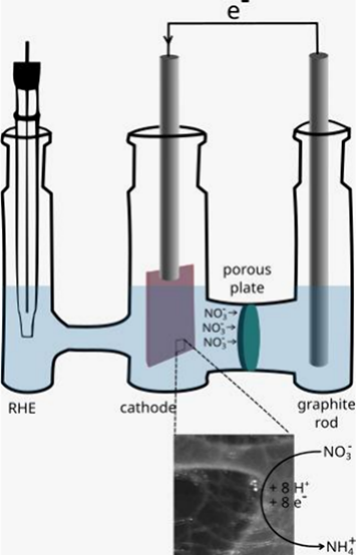

There has been huge interest among chemical scientists in the electrochemical reduction of nitrate (NO_3_^–^) to ammonia (NH_4_^+^) due to the useful application of NH_4_^+^ in nitrogen fertilizers and fuel. To conduct such a complex reduction reaction, which involves eight electrons and eight protons, one needs to develop high-performance (and stable) electrocatalysts that favor the formation of reaction intermediates that are selective toward ammonia production. In the present study, we developed and applied Co_3_O_4_/graphene nanoribbon (GNR) electrocatalysts with excellent properties for the effective reduction of NO_3_^–^ to NH_4_^+^, where NH_4_^+^ yield rate of 42.11 mg h^–1^ mg_cat_^–1^, FE of 98.7%, NO_3_^–^ conversion efficiency of 14.71%, and NH_4_^+^ selectivity of 100% were obtained, with the application of only 37.5 μg cm^–2^ of the catalysts (for the best catalyst —Co_3_O_4_(Cowt %55)GNR, only 20.6 μg cm^–2^ of Co was applied), confirmed by loadings ranging from 19–150 μg cm^–2^. The highly satisfactory results obtained from the application of the proposed catalysts were favored by high average values of electrochemically active surface area (ECSA) and low *R*_ct_ values, along with the presence of several planes in Co_3_O_4_ entangled with GNR and the occurrence of a kind of “(Co_3_(Co(CN)_6_)_2_(H_2_O)_12_)_1.333_ complex” structure on the catalyst surface, in addition to the effective migration of NO_3_^–^ from the cell cathodic branch to the anodic branch, which was confirmed by the experiment conducted using a H-cell separated by a Nafion 117 membrane. The in situ FTIR and Raman spectroscopy results helped identify the adsorbed intermediates, namely, NO_3_^–^, NO_2_^–^, NO, and NH_2_OH, and the final product NH_4_^+^, which are compatible with the proposed NO_3_^–^ electroreduction mechanism. The Density Functional Theory (DFT) calculations helped confirm that the Co_3_O_4_(Cowt %55)GNR catalyst exhibited a better performance in terms of nitrate electroreduction in comparison with Co_3_O_4_(Cowt %75), considering the intermediates identified by the in situ FTIR and Raman spectroscopy results and the rate-determining step (RDS) observed for the transition of *NO to *NHO (0.43 eV).

## Introduction

With the world facing imminent threats of climate change in our present time, the electrochemical conversion of nitrate (NO_3_^–^) to ammonia (NH_3_) has become extremely important to chemical scientists and the society at large, since the product of this conversion process can be used in diverse applications that can contribute to pollution reduction and environmental preservation. Among these applications include the following: (i) NH_3_ can be used in nitrogen fertilizers and fuels^1^ — green liquid and hydrogen-rich energy carrier, which is carbon-free and easily transportable^2^ — and as chemical precursors;^1^ (ii) NO_3_^–^ can be electrochemically converted to the nontoxic N_2_ gas, which is of great interest to environmental scientists;^3^ and (iii) NO_3_^–^ electrochemical conversion to NH_3_ can be used in place of the Haber–Bosch (H–B) industrial NH_3_ synthesis process (N_2_ + 3H_2_ → 2NH_3_— synthesis process that requires both high temperature and pressure: 350–550 °C, 150–350 atm^4^), which involves the consumption of a large amount of energy and high carbon emission.^5,6^ NH_3_ production under the H–B process involves the consumption of approximately 2% of global energy and the release of nearly 1.8 tons of CO_2_ per NH_3_ ton produced.^4^ The amount of CO_2_ released during the preparation of hydrogen gas used in the H–B process is equivalent to nearly 1.5% of global CO_2_ released from fossil fuels into the atmosphere.^7^ Due to the lower dissociation energy of the N=O bond (only 204 kJ mol^–1^), theoretically, NO_3_^–^ requires low overpotentials to be reduced to NH_3_.^8^

NO_3_^–^ pollution in the environment is caused by the rampant use of artificial fertilizers, undesirable industrial activities, fossil fuels combustion, and household/human waste accumulated in animals and plants, as well as in surface and underground waters.^9^ The presence of NO_3_^–^ in drinking water poses serious risks to human health; these risks include liver damage, cancer, and blue baby syndrome — linked to nitrite anions (NO_2_^–^, a product derived from NO_3_^–^ transformation),^10^ particularly when it is in concentrations higher than 10 mg L^–1^ in NO_3_^–^ – N (maximum contaminant levels (MCL) in terms of N mass per L^11^).^12^ Previously, researchers typically focused their attention on the electroreduction of NO_3_^–^/NO_2_^–^ (electrochemical denitrification) to N_2_; however, once the importance of ammonia as a green energy carrier gained ample recognition, the research interest has largely shifted toward the reduction of NO_*x*_^–^ to NH_3_. This approach, involving NO_*x*_^–^ reduction to NH_3_, not only removes N-pollutants from water but also leads to the synthesis of ammonia, which has greater economic value; the technique exhibits good reduction efficiency and does not involve any chemical input or secondary pollution.^13^ The eco-friendly electrochemical reduction of NO_3_^–^ (NO_3_^–^ reduction reaction, NO_3_^–^RR) to NH_3_ has been well documented in the literature, and the main goal, as can be seen in the vast array of studies reported on the subject matter, has been to develop highly effective electrocatalysts which are capable of promoting/enhancing the occurrence of the complex eight proton-coupled electron transfer process.^14^

A few examples of Co-based electrocatalysts that have been employed for the electrochemical reduction of NO_3_^–^ to ammonia include the following: amorphous cobalt phosphide nanoshuttles (CoP PANSs);^3^ metallic Co nanoarrays (Co-NAs);^9^ Co_2_AlO_4_ nanosheet array on carbon cloth (Co_2_AlO_4_/CC);^1^ core–shell Cu-CuO_*x*_ and Co-CoO phases on Cu foil;^5^ nanosheets of CoO_*x*_;^4^ nanosheet arrays of Co–CoO;^7^ cobalt–phosphorus alloy film supported on Ti plate;^8^ Ni foam with Cu_2_O or/and Co(OH)_*x*_ nanocomposites (Ni/Cu_2_O, Ni/Co(OH)_*x*_ and Ni/Cu_2_O/Co(OH)_*x*_);^12^ and 3D mesopore-rich Co-NC (NC = nitrogen-doped carbon).^2^ A number of Co_3_O_4_-based electrocatalysts have also been used for the production of ammonia through the electrochemical reduction of NO_3_^–^; among these electrocatalysts include the following: 3D structured Co_3_O_4_/CF electrode with Co(III), aiming at the adsorption of NO_3_^–^, while Co(II) favors the production of H*;^15^ Cu–Co_3_O_4_ nanowire arrays on carbon cloth which optimize the intermediate hydro-deoxygenation free-energy change during nitrate reduction;^16^ Co_3_O_4_ nanosheet arrays with cobalt vacancies on carbon cloth (v_Co_-Co_3_O_4_/CC) which help enhance the electron density on Co active sites;^17^ (Cu_0.6_Co_0.4_)Co_2_O_4_ in place of inert Co_Td_ (tetrahedral Co) sites with Cu^2+^ and optimized octahedral Co (Co_Oh_) sites;^18^ physical mixing of Cu_2_O and Co_3_O_4_ nanocubes ((100) lattice) on carbon paper (Cu_2_O+Co_3_O_4_ tandem catalyst; reduction of NO_3_^–^ to NO_2_^–^ on Cu_2_O, followed by the conversion of NO_2_^–^ to NH_3_ in the closely located Co_3_O_4_ particle);^19^ Co_3–x_Ni_*x*_O_4_ (Co_3–*x*_Ni_*x*_O_4_, *x* = 0, 0.5, 1, 1.5) nanoarray on carbon cloth with incorporated Ni which promotes the surface reconstruction of Co_3_O_4_ to Co_*y*_Ni_1–y_(OH)_2_ and tuning its electronic structure;^20^ Co_3_O_4_ nanoparticles embedded in porous carbon nanofibers (Co_3_O_4_@CNF), where CNF is beneficial for the dispersion of Co_3_O_4_, enhancing the conductivity, and with Co_3_O_4_ clusters exhibiting low NO_3_^–^ adsorption energy.^21^ Several studies reported in the literature have also employed Co_3_O_4_-based electrocatalysts for NO_3_^–^ electrochemical reduction to N_2_; among the electrocatalysts employed have included needle-like Co_3_O_4_ self-supported on cobalt foam (Co_3_O_4_/CF), with NH_4_^+^ transformed to N_2_ in the presence of Cl^–^ due to the electrochemical generation of ClO^–^^13^ and Co_3_O_4_–TiO_2_/Ti, where NO_3_^–^ reduction is mediated by the Co^2+^–Co^3+^–Co^2+^ redox cycle, resulting mostly in N_2_ when 2000 mg L^–1^ of chloride ions concentration is applied.^10^ Co_3_O_4_-based electrocatalysts have also been used for the electrochemical reduction of nitrogen oxyanions (NO_*x*_^–^ = NO_3_^–^ and NO_2_^–^) to ammonia (NH_3_); among the electrocatalysts employed for this purpose have included sulfur-modified Co_3_O_4_ spinel nanosheets (S–Co_3_O_4_) which help tailor the catalyst electronic structure^22^ and oxygen vacancy (O_v_)-rich Co_3_O_4_ nanoparticles, due to the abundance of O_v_ and the small particle size.^23^

Taking into account that several Co- and Co_3_O_4_-based electrocatalysts have been reported in the literature, and having no knowledge of the existence of studies related to the application of Co_3_O_4_ entangled with GNR and the development of a kind of ‘(Co_3_(Co(CN)_6_)_2_(H_2_O)_12_)_1.333_ complex’ for the electrochemical reduction of NO_3_^–^ to produce NH_4_^+^, in the present study, we employed a simple hydrothermal method for the production of Co_3_O_4_(Cowt %75), Co_3_O_4_(Cowt %38)GNR, Co_3_O_4_(Cowt %55)GNR, and Co_3_O_4_(Cowt %53)GNR electrocatalysts; the electrocatalysts were successfully applied for the effective reduction of NO_3_^–^ to NH_4_^+^ following the mechanism described by Anastasiadou et al.^24^ The effective reduction of NO_3_^–^ to NH_4_^+^ was confirmed by the in situ FTIR and Raman spectroscopy results which helped identify the adsorbed intermediates (NO_3_^–^, NO_2_^–^, NO, and NH_2_OH) and the final product NH_4_^+^. The application of the entangled Co_3_O_4_/GNR electrocatalysts in the presence of only 37.5 μg cm^–2^ (the loading range studied was 19–150 μg cm^–2^) of the catalysts (20.6 μg cm^–2^ of Co for the best catalyst —Co_3_O_4_(Cowt %55)GNR) resulted in an NH_4_^+^ yield rate of 42.11 mg h^–1^ mg_cat_^–1^, Faradaic efficiency (FE) of 98.7%, NO_3_^–^ conversion efficiency of 14.71%, and NH_4_^+^ selectivity of 100%. The density functional theorem (DFT) calculations involving the adsorbed intermediates, identified through the results obtained from the in situ FTIR and Raman spectroscopy analyses, confirmed that the adsorbed NO intermediate exhibits a lower energy transition to the next intermediate (*NO to *HNO, 0.43 eV for the RDS) for the Co_3_O_4_(Cowt %53)GNR in comparison with the Co_3_O_4_(Cowt %75) electrocatalyst (0.65 eV for the RDS).

## Experimental Section

### Reagents

The reagents employed in the experiments were as follows: HNO_3_ (70%; from Alphatec), H_2_SO_4_ (98%; from Merck), H_3_PO_4_ (85%; from Dinâmica), NaNO_3_ (99.5%; from Merck), HCl (37%; from Vetec), K_2_S_2_O_8_ (99%; from Merck), NO_2_ 100 ppm solution (from Sigma-Aldrich), multi cation standard 1 for IC (NH_4_^+^ 400 ppm; from Sigma-Aldrich), multi anion standard 1 for IC (NO_3_^–^ 20 ppm; from Sigma-Aldrich), NH_4_OH (solution 28 wt % in H_2_O; from Merck), H_2_O_2_ (30%; from Vetec), P_2_O_5_ (99%; from Vetec), K_2_SO_4_ (99%; from Sigma-Aldrich), urea (99%; from Neon), NaClO (10–12%; from Neon), KMnO_4_ (98%; from Nuclear), 4-(dimethylamino)benzaldehyde (99%; from Sigma-Aldrich), C_2_H_6_O (95%, from Vetec), nitric acid/dipiconilic acid solution 17 mM (from Sigma-Aldrich), Na_2_CO_3_/NaHCO_3_ 64/20 mM solution (from Sigma-Aldrich), CoCl_2_·6H_2_O (98%; from Sigma-Aldrich), NH_4_Cl (99.5%; from Sigma-Aldrich), phenol (99%; from Sigma-Aldrich), sulfanilamide (98%; from Sigma-Aldrich), N-(1-naphthyl)ethylenediamine dihydrochloride (98%; from Sigma-Aldrich), acid sulfamic (99%; from Sigma-Aldrich), sodium nitroprusside dihydrate (Na_2_[Fe(CN)_5_]NO·2H_2_O) (99%; from Sigma-Aldrich), Nafion (20 wt %; from Sigma-Aldrich), hydrazine sulfate (NH_2_NH_2_·H_2_SO_4_) (99%; from Sigma-Aldrich), and multiwalled carbon nanotubes (MWCNTs) with a dimension of 10 ± 1 ηm (external diameter) × 4.5 ± 0.5 ηm (internal diameter) and 3–6 μm long, with six to eight tube walls (from Sigma-Aldrich).

### Syntheses

The mechanism applied for the synthesis of the graphene nanoribbons (GNR)^25^ has been described in detail in the Supporting Information.

The Co_3_O_4_(Cowt %55)GNR sample was produced by mixing 48 mg of GNR, 300 mg of CoCl_2_·6H_2_O, 1.5 g of urea, and 90 mL of ultrapure water in a beaker and sonicating the mixture for 40 min. Subsequently, the dispersion was transferred to a Teflon-lined stainless-steel autoclave and kept at 180 °C for 24 h. After cooling at room temperature, the product was washed with ultrapure water several times by centrifugation and then dried in an oven at 40 °C for 24 h (see Scheme S1). It is noteworthy that the hybrid Co_3_O_4_/carbon nanotube (CNT) has been produced via the hydrothermal method in lower temperature and time (150 °C, 3 h) using Co(OAc)_2_, NH_4_OH, and CNT;^26^ these synthesis conditions are different from those employed in our present work.

For the synthesis of the bare Co_3_O_4_(Cowt %75) sample, we employed the same procedure described above, but in the absence of GNR. To produce the Co_3_O_4_(Cowt %38)GNR and Co_3_O_4_(Cowt %53)GNR samples, the same procedure was also employed, but in the presence of 150 and 450 mg of CoCl_2_·6H_2_O, respectively, instead of 300 mg of CoCl_2_·6H_2_O. Importantly, the application of 450 mg of CoCl_2_·6H_2_O in the synthesis of the Co_3_O_4_(Cowt %53)GNR sample did not lead to a better performance in terms of NH_4_^+^production from the electrochemical reduction of NO_3_^–^, as will be proven below. In addition, the presence of a lower amount of Co (wt %53) in the Co_3_O_4_(Cowt %53)GNR sample in comparison with the Co_3_O_4_(Cowt %55)GNR sample suggests that the maximum Co loading is obtained during the synthesis of the Co_3_O_4_(Cowt %55)GNR sample (which has been found to be the best catalyst in the present work). In view of that, the results obtained for the Co_3_O_4_(Cowt %53)GNR sample will not be discussed here.

### Electrode Preparation

The carbon paper (CP) sheet cleaning process was initiated through a leaching process aimed at removing any residual metal impregnated in the CP. In a beaker, the CP sheet was placed in a 30 mL of 0.5 M H_2_SO_4_/0.5 M HNO_3_ solution and sonicated for 20 min; next, the sheet was heated at 50 °C on a hot plate for 8 h.^27^ The CP sheet was then washed several times in ultrapure water until a neutral pH was obtained; after that, the material was dried at room temperature.

Subsequently, a uniform thin film was produced on the CP electrode surface by dripping an aqueous solution of GNR, Co_3_O_4_(Cowt %75), Co_3_O_4_(Cowt %38)GNR, Co_3_O_4_(Cowt %55)GNR, and Co_3_O_4_(Cowt %53)GNR (resulting in a surface loading of 37.5 μg cm^–2^; the ink solution contained 0.1% (v/v) Nafion) on the CP electrode surface; in addition, 10 μL of 0.1% Nafion was also poured on the surface of the catalyst film. The thin films were dried at room temperature. After that, the modified electrodes were immersed in ultrapure water before being placed in the electrochemical cell.

### Apparatuses and Measurements

In general, the electrochemical experiments were conducted in a H-type glass cell containing anodic and cathodic branches separated by sintered glass with porosity of 4 (10 to 16 μm); the cell consisted of three electrodes: a CP sheet (1.0 cm^2^), a reversible hydrogen electrode (RHE), and a graphite rod, which were used as working, reference, and counter electrodes, respectively. The distance between the working electrode and the counter electrode was 7.6 cm. 0.1 M K_2_SO_4_ was employed as the supporting electrolyte. After the electrochemical experiments were performed in 0.1 M K_2_SO_4_, different concentrations of NaNO_3_ were added into the cathodic branch. The solutions were saturated with Ar (5.0 purity, acquired from White Martins). The bare CP or modified CP electrode was considered to have been “stabilized” for the electrochemical results to be recorded after being subjected to the following analyses: three cyclic voltammetry (CV) analyses in the potential range of 0.7 to −0.2 V (50 mV s^–1^); three stationary linear sweep voltammetry (LSV) analyses in the potential range of 0.2 to −1.0 V (5 mV s^–1^); and ten CV analyses from 0.7 to −0.2 V (50 mV s^–1^). Specifically, for the analysis of the electrochemical behavior of the CVs shown in the Supporting Information, we employed a single-compartment glass cell with the same electrodes described above.

The CV and LSV analyses were performed using a bipotentiostat AFCBP1 (Pine Research Instrumentation). For the electrochemical impedance spectroscopy (EIS) analyses, we employed a PGSTAT-128N potentiostat-galvanostat (Autolab), equipped with the FRA2.X module. EIS measurements were performed in the frequency range of 10 mHz to 100 kHz, with disturbance potential of 10 mV (rms).

The CV analyses conducted at different potential scan rates in a nonfaradaic potential region, centered around the open circuit potential (OCP) region, and in the potential window of 0.1 V were used to calculate the double-layer capacitance (*C*_dl_)^27^ based on eq 1 below:
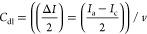
1where *I*_a_ and *I*_c_ stand for the anodic and cathodic currents, respectively, in the middle of the CV potential window, and *v* is the potential scan rate.

The ECSA values were obtained by dividing the C_dl_ values by the specific capacitance (*C*_s_) value, considered here as 0.040 mF cm^–2^,^28^ in 0.1 M K_2_SO_4_.

The morphology and distribution of the nanocomposites and nanoparticles were characterized by transmission electron microcopy (TEM) using FEI TECNAI G^2^ F20 HR-TEM equipment, operated at 200 kV. The composite films were also characterized by scanning electron microscopy with a field emission gun (SEM-FEG), using a JEOL JSM 7200F, coupled to energy dispersive X-ray spectroscopy (EDS).

The Raman spectral data were recorded using a LabRam HR Evolution micro-Raman spectrometer (Horiba Jobin-Yvon) at room temperature; this was done using a solid-state laser operating at 633 nm, a standard grid (600 grmm^–1^) and an EMCCD detector (Synapse EM). The samples were excited with a low-intensity laser (2 mW) in order to avoid overheating and the occurrence of photochemical phenomena. A 100 objective lens (Olympus, MPlan N) was used to focus the laser on the sample. The spectra were collected in an acquisition time of 12 s. The in situ Raman spectral data were recorded using a LabRam HR Evolution micro-Raman spectrometer (Horiba Jobin-Yvon) at room temperature; this was done using a solid-state laser operating at 473 nm and an EMCCD detector (Synapse EM). In the electrodes cell (screen-printed electrode, Metrohm), which consisted of a carbon working electrode (0.126 cm^2^), a carbon counter electrode, and a Ag reference electrode with a solution drop covering the electrodes, the modified carbon working electrode was excited with a 12.5 mW intensity laser in order to avoid overheating and the occurrence of photochemical phenomena. A 600 objective lens (Olympus, MPlan N) was used to focus the laser on the modified carbon working electrode. The spectra were collected in an acquisition time of 1.0 s. The conversion of Ag/AgCl (assumed as approximated to Ag) to RHE potential was conducted by obtaining the potential for Pt plate electrode in the presence of 0.1 M K_2_SO_4_ or 0.1 M K_2_SO_4_ + 40 mM NaNO_3_ solution saturated with H_2_ gas against RHE and Ag/AgCl reference electrodes and taking the solution pH (measured) into account.^29^

The in situ FTIR spectral data (1000 to 1650 cm^–1^) were recorded using a FTIR Vertex 70v spectrometer (Bruker) equipped with an evacuated optical bench and reflection unit directed toward an electrochemical cell containing a glassy carbon (GC, 0.385 cm^2^) working electrode modified with a catalyst, a RHE reference electrode, and a Pt counter electrode.

To measure the elemental composition of the surface, X-ray photoelectron spectroscopy (XPS) analyses were carried out using a PHI Quantera II. The Al Kα line (1486.6 eV), which was operated at 15 kV and 25 W, was used as the ionization source. After background subtraction was performed, the spectra were deconvoluted using a combination of Lorentzian (30%) and Gaussian Voigt (70%).

The crystal structure of the composites was analyzed by X-ray diffraction (XRD) using a Bruker D8 Advance X-ray diffractometer, operated under the following conditions: potential of 40 kV and current of 40 mA (1.6 kW). The experimental parameters adopted included the following: scanning rate of 0.02° s^–1^ at 2θ; Cu – Kα radiation with λ = 1.540501 Å; energy of 8.047 keV; and Si powder employed as the reference standard.

Elemental analyses (EA) were performed using the Scientific Flash 2000 CHNS/O Elemental Analyzer Thermo equipment, under cycle operating conditions (run time) of 720 s and oven temperature of 950 °C for CHNS determinations, and under the cycle (run time) of 400 s and oven temperature of 1060 °C for O determination.

The thermogravimetric characterization analyses were performed using a Shimadzu TGA-50 thermogravimetric analyzer, with a flow of synthetic air (50 mL min^–1^) at temperatures ranging from 30 to 905 °C, and a heating rate of 10 °C min^–1^, using a sample mass of 5 mg in a platinum cell.

The procedure involving the samples digestions was performed as described in^30^ and based on the following steps: 10 mL of HNO_3_ (65%) was added in 2 mg of all samples in a glass beaker, covered with a watch glass. The solution was kept under heating and stirring for 2 h at 85 °C until the brown-colored NO_2_ disappeared. After the solution was cooled, 2 mL of HClO_4_ was added therein, and the mixture was heated at 200 °C until fumes of HClO_4_ appeared. The solution was then cooled at room temperature and transferred into a PTFE evaporating dish; after that, 5 mL of HF (40%) was added into the solution, which was then subjected to stirring until complete evaporation. The final residue was completely dissolved in concentrated HCl and transferred to a volumetric flask of 10 mL. All the samples, including the blank sample, were subjected to the same procedure. For further analyses, an aliquot of 0.5 mL of the samples was diluted in another volumetric flask of 10 mL, in the presence of ultrapure water. The samples were subjected to atomic absorption spectroscopy (AAS) in order to measure the Co concentration in the heterostructures; the analyses were conducted using the AAS equipment from PerkinElmer PinAAcle 900T.

The ultraviolet–visible (UV–visible) absorbance responses were obtained from a Hitachi U-3000 spectrophotometer.

Ion chromatographic analyses were carried out using a 930 Compact IC Flex (Metrohm) ion chromatograph with a conductivity detector. For the analysis in the cation mode, ammonia was determined using a Metrosep C6–100/4.0 cation column at a flow rate of 0.9 mL min^–1^, where 1.7 mM nitric acid/dipiconylic acid was applied as eluent. For the analysis in the anion mode, nitrate and nitrite were determined using a MetrosepA Supp 5–150/4.0 chromatographic column at a flow rate of 0.7 mL min^–1^, with 3.2 mM Na_2_CO_3_/1.0 mM NaHCO_3_ applied as eluent. In both analyses (cation and anions), a looping of 20 μL was employed.

The NH_4_^+^ yield or production rate in a specified applied potential was calculated as follows:
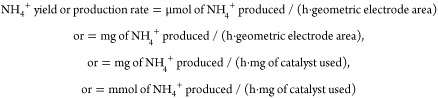
2The nitrate conversion efficiency (%)^12^ was determined as follows:
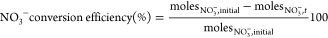
3Where moles_NO_3_^–^__, initial_ stands for the nitrate moles in the cell cathodic branch at time zero minus the nitrate moles that migrated to the cell anodic branch at each time; moles_NO_3_^–^__, *t*_ represents the nitrate moles that remained in the cell cathodic branch at time *t*.

The nitrate migration (%) from the cell cathodic branch to the anodic branch was determined as follows:
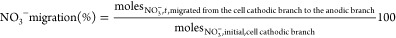
4Where moles_NO_3_^–^__, initial, cell cathodic branch_ stands for the nitrate moles in the cell cathodic branch at time zero; and moles_NO_3_^–^__, *t*, migrated from the cell cathodic branch to the anodic branch_ is the nitrate moles in the cell anodic branch at time t.

NH_4_^+^ selectivity (%)^12^ was determined as follows:
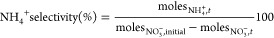
5where moles_NH_4_^+^__,*t*_ is the ammonia moles at time t.

The faradaic efficiency (FE (%)) was determined as follows:^9^
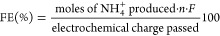
6where *n* is equal to 8 for NH_4_^+^ production from NO_3_^–^ reduction; and *F* is the Faraday constant (96 485 C mol^–1^).

## Computational Methods

All the DFT calculations were performed using the GPAW code^31,32^—an efficient and flexible tool for electronic structure calculations. The exchange-correlation interactions were treated using the Perdew–Burke–Ernzerhof (PBE) functional,^33^ which is a widely employed generalized gradient approximation (GGA) method. A plane-wave basis set was employed with an energy cutoff of 450 eV, where accurate results were secured while maintaining computational efficiency.

For the Brillouin zone integration, a Monkhorst–Pack^34^ k-point grid of 4 × 4 × 1 was used. The total energy convergence criterion was set such that the change in absolute energy between iterations was less than 1 × 10^–5^ eV. In the geometry optimization steps, the system was considered converged when the forces acting on each atom were reduced to below 0.02 eV/Å.

The modeling of the Co_3_O_4_(111) surface, based on the XRD and HR-TEM results (see below), and the Co_3_O_4_(111)(Cowt %55)GNR composite was conducted using a (2 × 2) supercell, as depicted in Figure S1. A vacuum region of 15 Å was used to separate adjacent slabs. First, the structure of Co_3_O_4_(111) was fully optimized in order to determine its equilibrium geometry. After that, the GNR was introduced into the system, and the combined structure was relaxed again to account for the interactions between Co_3_O_4_ and the GNR.

Subsequently, the adsorbate species—NO_3_, NO_2_, NO, NHO, NH_2_O, NH_2_OH, NH_2_, and NH_3_—were positioned at their respective adsorption sites. A relaxation process was then carried out so as to accommodate any structural changes induced by the adsorption.

In addition to the slab calculations, the total energies of the gas-phase species — H_2_O, NO_3_, and H_2_, were computed for comparison purposes; this facilitated an accurate evaluation of the adsorption energies.

**Scheme 1:**
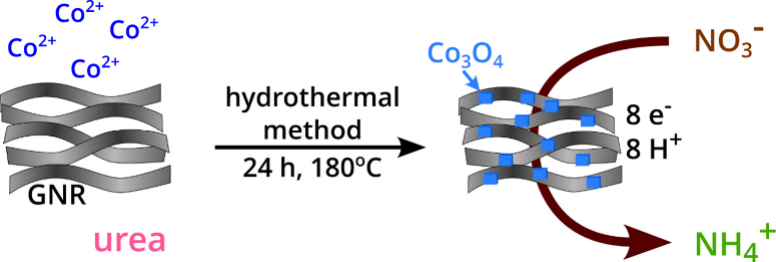
Schematic Illustration of the Mechanism Involving the Preparation of the Co_3_O_4_/GNR Materials and Their Application in the Electrochemical Reduction of NO_3_^–^ to NH_4_^+^

Scheme 1 presents an outline of the design concept of the Co_3_O_4_/GNR materials and their relation to NH_4_^+^ production from the NO_3_^–^ electroreduction.

## Results and Discussion

### Material Characterization

**Figure 1:**
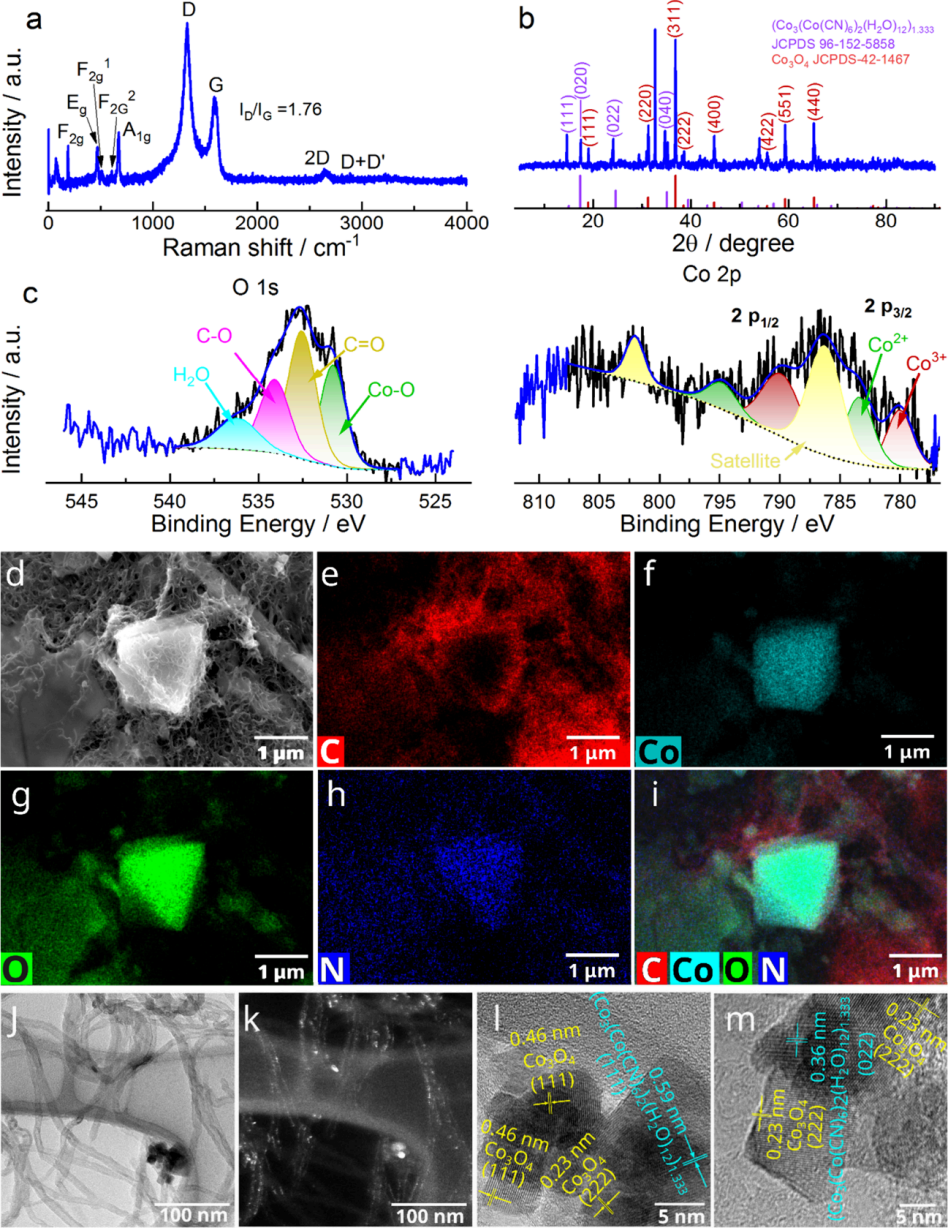
(a) Raman spectrum; (b) XRD spectrum; (c) O 1s and Co 2p HR-XPS spectra; (d-i) SEM mapping images; (j-k) TEM images; and (l-m) HR-TEM images for the Co_3_O_4_(Cowt %55)GNR sample.

In order to gain direct insights into the crystallinity and defects within the Co_3_O_4_/GNR catalysts, including the identification of edges within the GNR, Raman spectroscopy experiments were performed. Figures 1a and S2a show the Raman spectra obtained for the bare GNR(Cowt %0) and Co_3_O_4_(Cowt %75) samples, as well as for the different Co_3_O_4_/GNR samples investigated.

The Raman spectra obtained for the Co_3_O_4_(Cowt %55)GNR (Figure 1a) and the bare Co_3_O_4_(Cowt %75) and Co_3_O_4_(Cowt %38)GNR (Figure S2a) catalysts show strong vibration peaks, on average, at 188, 469, 512, 607, and 673 cm^–1^, corresponding to F_2g_, E_g_, F_2g_^1^, F_2g_^2^, and A_1g_ Raman-active modes of the Co_3_O_4_ cubic phase.^35−37^ Based on the studies reported in the literature, the E_g_ and F_2g_ vibrations of the spinel lattice are considered to be associated with both Co^2+^ and Co^3+^ ions, whereas the octahedrally coordinated Co^3+^ ions are found to contribute solely to the strong A_1g_ Raman band.^38^

The Raman spectra obtained for the bare GNR(Cowt %0), Co_3_O_4_(Cowt %38)GNR, and Co_3_O_4_(Cowt %55)GNR catalysts also exhibit prominent first-order bands: the disorder band (D band) at approximately 1326 cm^–1^ and the graphite band (G band) at around 1592 cm^–1^.^25,39−42^ Compared to the G band, the relatively higher intensity of the D band is indicative of the contribution of GNR edge defects^25,40,42^ to the bare GNR(Cowt %0), Co_3_O_4_(Cowt %38)GNR and Co_3_O_4_(Cowt %55)GNR catalysts; the *I*_D_/*I*_G_ ratio in Figures 1a and S2a signifies the relative intensity of the D and G bands. Furthermore, the Raman spectra show lower-intensity signals which correspond to double-resonant bands assigned to 2D^43^ and D+D′^43^ bands at approximately 2648 and 2923 cm^–1^, respectively.^40,42,43^ Typically, the 2D band is found to be a valuable metric, as it helps one to discern the variations in sheet stacking and graphene layer count.^25,44^ The D+D′ band observed in the Raman spectra is associated with the disorder-induced damaged graphene.^42,45^

The Raman data confirmed the presence of Co_3_O_4_ and GNR (which essentially constitute the nanocomposites produced in the present work). The Co_3_O_4_ structure is found to contain Co^2+^ and Co^3+^ ions, and GNR is known for its higher conductivity (electronic properties).^46^ Both Co_3_O_4_ and GNR are found to effectively contribute to nitrate electroreduction, as will be proven below.

X-ray diffraction (XRD) analysis was also used to identify the kind of Co oxide supported in the GNR. Figure 1b shows the diffraction patterns obtained for the Co_3_O_4_(Cowt %55)GNR sample and Figure S2b shows the diffraction patterns obtained for the bare GNR(Cowt %0) and Co_3_O_4_(Cowt %75) samples, as well as for the different Co_3_O_4_/GNR samples investigated.

For the Co_3_O_4_(Cowt %55)GNR (Figure 1b) and the different Co_3_O_4_/GNR(Figure S2b) samples investigated, we identified the presence of several peaks related to Co_3_O_4_ (see the Supporting Information for the peaks attributions), in addition to the peaks at 2θ of 14.6, 17.5, 24.1, and 34.7° (0.59, 0.51, 0.36, and 0.25 nm, respectively), which correspond to the (111), (020), (022), and (040) planes, respectively, and are related to the (Co_3_(Co(CN)_6_)_2_(H_2_O)_12_)_1.333_ complex (JCPDS 96–152–5858); this result further reinforces the bond involving Co atoms with carbon and some nitrogen atoms present in the GNR. Also, we observed the presence of a more intense peak at 2θ of 32.7°, related to the Co_3_O_4_(Cowt %55)GNR catalyst, and which is most likely associated with the bond involving Co atoms with carbon and some nitrogen atoms present in the GNR. The XRD results confirm that the Co_3_O_4_/GNR structures are constituted by Co_3_O_4_ and Co atoms which are bonded with carbon and some nitrogen atoms present in the GNR. See the discussion on the bare GNR(Cowt %0) and Co_3_O_4_(Cowt %75) samples in the Supporting Information.

The XPS survey spectra (Figure S3) obtained for the different samples are discussed in the Supporting Information (see Table S1). The results obtained from the elemental analyses (Table S2) and TG responses (Figure S4) are also discussed in the Supporting Information.^25,42,47^

The data obtained from the AAS analysis (Table S3) were used to quantify the amount of Co (wt %) present in the samples investigated and to name the samples, as has been done previously. The amount of Co recorded for the bare Co_3_O_4_(Cowt %75) sample (wt. 75%) was very close to the theoretical value expected for a pure Co_3_O_4_ sample (wt. 74%); this confirms that the bare Co_3_O_4_(Cowt %75) sample is pure, as observed from the XRD result (Figure S2b). Furthermore, the amount of Co recorded for the other samples was also in line with our expectations; the “saturation” of Co observed mostly in the form of Co_3_O_4_ occurred in the Co_3_O_4_(Cowt %55)GNR sample.

The O 1s high-resolution XPS (HR-XPS) spectra obtained for the Co_3_O_4_(Cowt %55)GNR sample (Figure 1c), bare GNR(Cowt %0) and Co_3_O_4_(Cowt %75) samples, and for the different Co_3_O_4_/GNR samples (Figure S2c) show, in general, a broad peak — with the exception of the Co_3_O_4_GNR poststability sample (two peaks), deconvoluted into four peaks. The Co_3_O_4_(Cowt %55)GNR (Figure 1c) and the other samples (Figure S2c) containing Co_3_O_4_ exhibited four deconvoluted peaks related to the O 1s HR-XPS spectrum; these peaks were attributed to the chemical states of Co–O, C=O, C–O, and H_2_O, and were positioned, on average, at 529.7, 531.4, 533.3, and 535.1 eV, respectively (Table S4).^27,48^ The identification of C=O, C–O, and H_2_O chemical states for the bare Co_3_O_4_(Cowt %75) sample (Figure S2c) is attributed to the fact that the sample was supported by carbon tape during the XPS measurements. The average % of contents recorded were 18.1, 28.8, 35.2, and 17.9 for the Co–O, C=O, C–O, and H_2_O chemical states, respectively (Table S4). The main contributions to the % of contents were found to come from C–O and C=O, followed by Co–O; this clearly points to the relevance of Co_3_O_4_ entangled with GNR when it comes to catalytic responses in nitrate reduction.

The Co 2p HR-XPS spectra recorded for the Co_3_O_4_(Cowt %55)GNR (Figure 1c) and the other samples containing Co (Figure S2c) show, in general, two peaks (the bare GNR(Cowt %0) sample does not show any peak, only noise, Figure S2c), with an average ratio of 1.8:1, and their respective satellite shake-ups corresponding to 2p_3/2_ and 2p_1/2_ levels, respectively.^27^ These two peaks were deconvoluted into two additional peaks, corresponding to Co^3+^, on average, at 780.3 and 793.7 eV and Co^2+^ at 783.3 and 796.4 eV, respectively, with satellites observed at 787.3 and 802.4 eV(Table S4).^27^ The spin–orbital splitting of 13.3 eV, on average, between the peaks and the presence of the satellite peaks clearly point to the presence of Co^3+^ and Co^2+^ species in the bare Co_3_O_4_(Cowt %75) sample, as well as in the different Co_3_O_4_/GNR samples and the Co_3_O_4_GNR poststability sample (Figures 1c and S2c and Table S4). The Co^3+^and Co^2+^ species exhibit content percentages of 33.3% and 29.6%, on average, respectively (Table S4); this is totally consistent with the presence of cobalt spinel (II, III) or Co_3_O_4_.^49^

A thorough discussion on the C 1s HR-XPS^25,42,50^ (Figure S5) can be found in the Supporting Information. Figure S6 shows that the HR-XPS spectra obtained for P 2p, S 2p, Cl 2p, N 1p, Mn 2p, and Fe 2p, mostly constituted by noise, indicated that the N element exhibited low signal, and this prevented us from being able to quantify it, as well as the P and S elements; the result also shows that, during the GNR synthesis process, low amounts of contaminants (Cl, Mn, and Fe) remained in the samples.

For further characterization analysis, the SEM image of the Co_3_O_4_(Cowt %55)GNR sample (Figure S7d) shows some kind of Co_3_O_4_ “cloud” involving/covering the GNR, which appears to be more “solid” and with some Co_3_O_4_ nanoparticles in the Co_3_O_4_GNR poststability sample (Figure S7e). The SEM images obtained for the other catalysts are discussed in the Supporting Information.

The SEM mapping images obtained for the Co_3_O_4_(Cowt %55)GNR sample (Figure 1d-i) show the presence of the C element, which is quite well distributed in the sample, though with less “density” when covered by the Co element which appears (Co element) more intensely only in the pyramidal Co_3_O_4_ structure. The O element follows mostly the Co_3_O_4_ structures. The N element can be found to be quite well distributed throughout the sample, certainly with less “density”; this element accompanies both the intensity of the O element and that of the C element. This mapping response shows that there is a combination involving the C, Co, O and N elements in the Co_3_O_4_(Cowt %55)GNR sample. This combination was identified in the XRD response for the Co_3_O_4_(Cowt %55)GNR sample (Figure 1b), and it could contribute to the most effective outcome in nitrate electrochemical reduction observed for this sample, as will be demonstrated below. The SEM mapping images (Figure S8) obtained for the other samples are discussed in the Supporting Information.

The TEM images obtained for the Co_3_O_4_(Cowt %55)GNR sample showed the presence of a sufficient amount of GNR and a small agglomerate of Co_3_O_4_ crystals (Figure 1j), with the Co element (Co_3_O_4_) clearly covering (entangled with) the GNR (Figure 1k). The term “entanglement” used in the manuscript is understood as Co_3_O_4_ crystals being well dispersed in GNR. The HR-TEM images (Figures 1l-m) show the presence of small crystals (with average size of 10.3 nm; these particles agglomerate to form bigger particles with average size of 24.2 nm (Figure 1j) and even larger particles such as the ones shown in Figure 1d) and plates under the GNR, with finger patterns of (222) and (111) Co_3_O_4_ exposed planes with distances of 0.23 and 0.46 nm (JCPDS 42–1467), respectively, and (111) and (022) of the (Co_3_(Co(CN)_6_)_2_(H_2_O)_12_)_1.333_ complex exposed planes with distances of 0.59 and 0.36 nm (JCPDS 96–152–5858), respectively. The diffraction pattern (Figure S9n) of the image displayed in Figure S9m showed the ring diffraction related to (111) and (222) Co_3_O_4_, as well as (022) and (242) of the (Co_3_(Co(CN)_6_)_2_(H_2_O)_12_)_1.333_ complex planes (JCPDS 42–1467 and 96–152–5858). The TEM and HR-TEM images, the images used to produce the electron diffraction patterns, and the electron diffraction pattern images (Figure S9) obtained for the other samples are all discussed in the Supporting Information.

In summary, the TEM and HR-TEM images, the images used to produce the electron diffraction patterns, and the electron diffraction pattern images showed that the entanglement of Co_3_O_4_ and GNR, which encompasses the appearance of the ‘(Co_3_(Co(CN)_6_)_2_(H_2_O)_12_)_1.333_ complex’, is more effective in the Co_3_O_4_(Cowt %55)GNR sample, as already observed from the XRD data (Figure 1b), as well as from the SEM mapping images (Figures 1d-i and Figure S8), and the TG (Figure S4) and AAS combined results.

### Electrochemical Analysis

#### CV Profile, ECSA, and Impedance Spectroscopy

Figure S10 shows the cyclic voltammetry (CV) profiles obtained from the application of two potential windows: (i) from 1.65 to −0.35 V (Figure S10a – S10e); and (ii) from 0.70 to −0.70 V (Figure S10f–j); these CV profiles are thoroughly discussed in the Supporting Information. The key information derived from the profiles is that the bare GNR(Cowt %0) sample (Figure S10e) shows a discrete redox couple at around 1.0 V, which is typically characteristic of hydroquinone/quinone oxi-reduction.^42^ The bare Co_3_O_4_(Cowt %75) sample shows a well-defined current density peak at around 1.40 V (Figure S10d), which is typically characteristic of Co^2+^–Co^3+^ oxidation.^51^ In the Co_3_O_4_(Cowt %55)GNR sample, the current densities related to NO_3_^–^ reduction are observed in potentials more negative than 0.03 V (Figure S10g); remarkably, this sample exhibited the most positive potential among all the samples–in terms of current densities associated with NO_3_^–^ reduction.

The C_dl_ values (eq 1) obtained from the inclination of Figures S11 and S13 (acquired from CV profiles shown in Figures S12 and S14) are summarized in Tables S5 and S6. For the Co_3_O_4_(Cowt %55)GNR sample in the presence of varying concentrations of NaNO_3_ (Table S5), we observed no significant differences in the ECSA values (∼11.5 cm^2^, with the exception of 70 mM NaNO_3_, which recorded an ECSA value of 5.9 cm^2^); the ECSA values recorded for this sample were much higher than those recorded for the bare CP (0.8 cm^2^) and bare Co_3_O_4_(Cowt %75) (2.7 cm^2^) samples but lower than those of the bare GNR(Cowt %0) (46.3 cm^2^) and Co_3_O_4_(Cowt %38)GNR samples (22.5 cm^2^) at NaNO_3_ concentration of 40 mM (Table S6). These results are found to be in total agreement with the CV profiles shown in Figure S10. In essence, the results show that the ideal catalyst (Co_3_O_4_(Cowt %55)GNR) should display an improved ECSA value compared to that of the bare Co_3_O_4_ catalyst (entanglement between Co_3_O_4_ and GNR, with the presence of the ‘(Co_3_(Co(CN)_6_)_2_(H_2_O)_12_)_1.333_ complex’), though the value should not be as high as that of the Co_3_O_4_(Cowt %38)GNR catalyst, as will be elucidated below.

**Figure 2:**
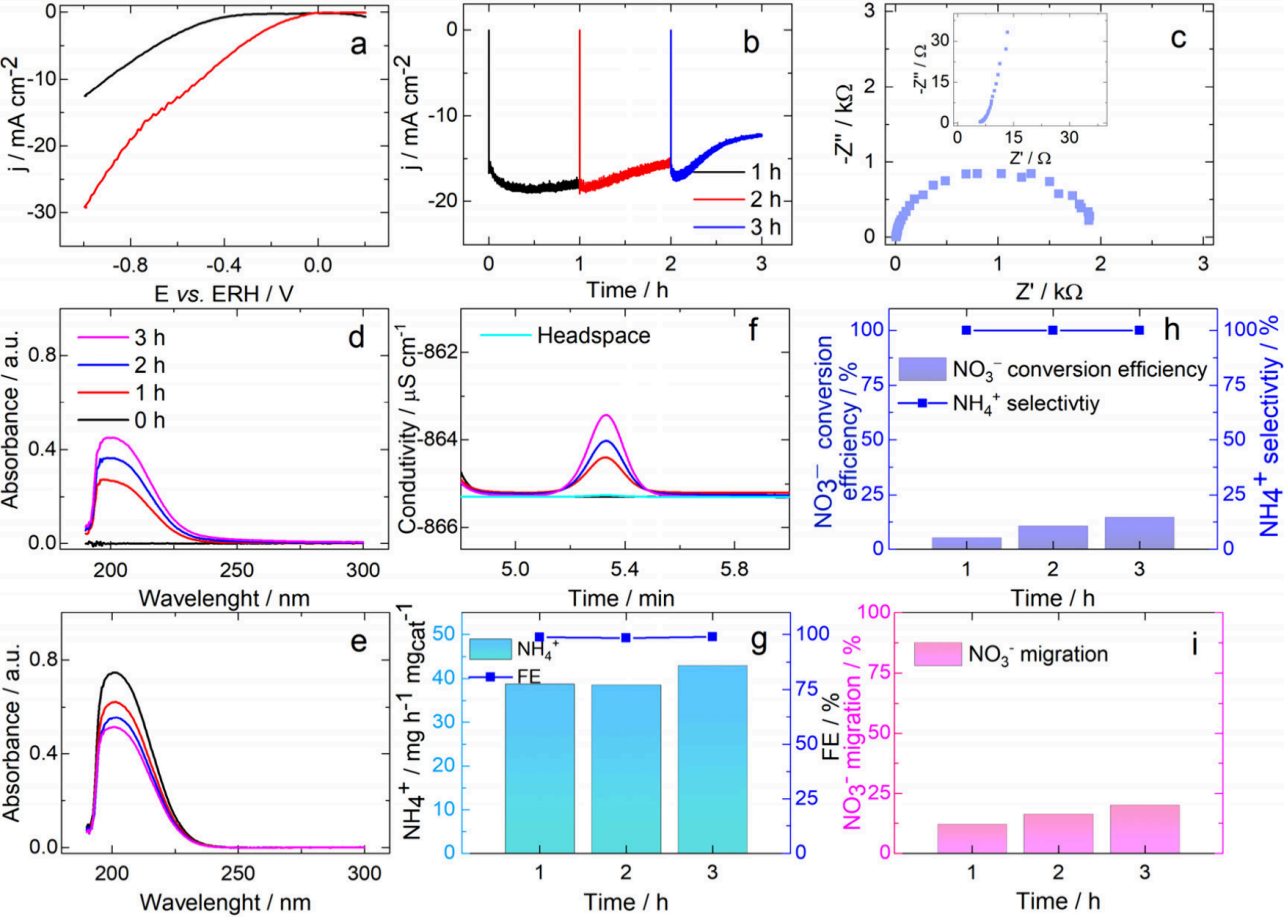
Responses obtained for the CP electrode modified with 37.5 μg cm^–2^ of Co_3_O_4_(Cowt %55)GNR: (a) stationary linear sweep voltammograms (LSVs) recorded at ν = 5 mV s^–1^ based on the application of Ar-saturated 0.1 M K_2_SO_4_ (black line) as supporting electrolyte, in the presence of 40 mM NaNO_3_ (red line) in the H-cell (separated by sintered glass) cathodic branch. Scans were initiated at 0.2 V; (b) chronoamperometric result (−0.6 V) obtained based on the application of 0.1 M K_2_SO_4_ as electrolyte solution, in the presence of 40 mM NaNO_3_ in the H-cell cathodic branch. After each 1 h of chronoamperometric experiment, the experiment was interrupted in order to remove aliquots from the solution; (c) EIS result (Nyquist-plot) obtained from the application of 0.1 M K_2_SO_4_ as supporting electrolyte, in the presence of 40 mM NaNO_3_ in the H-cell cathodic branch; UV curves used to quantify NO_3_^–^ in the H-cell (d) anodic (60 μL) and (e) cathodic (20 μL) branches during the chronoamperometric experiments conducted at −0.6 V; (f) IC curves used to quantify NH_4_^+^ (100 μL) in the H-cell cathodic branch during the chronoamperometric experiments conducted at −0.6 V; (h) NO_3_^–^ conversion efficiency and NH_4_^+^ selectivity, (i) NO_3_^–^ migration, and (g) NH_4_^+^ yield rate and FE values obtained after 3 h of chronoamperometric experiments conducted based on the application of the Co_3_O_4_(Cowt %55)GNR catalyst in Ar-saturated 0.1 M K_2_SO_4_, in the presence of 40 mM NaNO_3_, at a potential of −0.6 V.

Figures 2c and S15 show the Nyquist plots obtained for the samples (EIS results). It is important to note that due to the complexity of the reaction system (NO_3_^–^ electroreduction with excessive byproducts), the EIS results obtained in this study will be discussed only in terms of *R*_s_ and general *R*_ct_ values, with a view to comparing the catalysts investigated; the *R*_ct_ values will be used as a probable guide to identifying the “best catalyst”. As can be observed, the plots (see Figure 2c and Figure S15) show that the electrolyte solution resistance (*R*_s_) obtained was 8.4 Ω, on average, (Table S7) for the different NaNO_3_ concentrations and different catalysts investigated, with the exception of the bare CP electrode (34.5 Ω). Regarding the charge transfer resistance (*R*_ct_), the Co_3_O_4_(Cowt %38)GNR catalyst recorded the lowest *R*_ct_ value (0.7 kΩ) (Table S7) in the presence of 40 mM NaNO_3_, while the bare CP catalyst recorded the highest *R*_ct_ value (≫60 kΩ). The combination of Co_3_O_4_ and GNR leads to a considerable decrease in the *R*_ct_ value of the bare Co_3_O_4_(Cowt %75) sample (Table S7). However, to effectively produce ammonia, the optimal *R*_ct_ value for the best catalyst (Co_3_O_4_(Cowt %55)GNR) at the best NaNO_3_ concentration (40 mM) is 1.9 kΩ (Table S7), as explained below. The average solution pH ranged from 9.5, before the chronoamperometry experiments to 11.2, after these experiments (Table S7).

#### LSV and Chronoamperometry

The responses obtained from the LSV analysis are shown in Figures 2a and Figures S16 and 17; looking at the responses, one will observe that, at the current density of −10 mA cm^–2^, the Co_3_O_4_(Cowt %55)GNR catalyst recorded the highest overpotential (η) that catalyzes the nitrate reduction when 40 mM NaNO_3_ is applied (Figure 2a, η = 403 mV). The closest η is obtained at 70 mM NaNO_3_ (Figure S16g, η = 390 mV), while the lowest η is obtained at 10 mM NaNO_3_ (Figure S16a, η = 43.5 mV). The Co_3_O_4_(Cowt %38)GNR catalyst in the presence of 40 mM NaNO_3_ (Figure S17b) recorded an η of 227 mV. The bare Co_3_O_4_(Cowt %75)catalyst recorded an η of −54 mV (Figure S17c). These results further confirm that the best catalyst for nitrate reduction in the present study is Co_3_O_4_(Cowt %55)GNR. It is worth pointing out that the LSV responses shown in Figures 2a and S16 and S17 are not based on *iR* drop compensation, even though our H-cell R_s_ results (Table S7) allow us to perform 100% of *iR* drop compensation.

The results obtained from the chronoamperometric experiments conducted are shown in Figures 2b and S18–20. The “pulses” observed after each one h of chronoamperometric experiment are attributed to the interruption of the experiment in order to allow the withdrawal of aliquots.

The Co_3_O_4_(Cowt %55)GNR catalyst exhibited more negative current densities, on average, during the 3 h period of the chronoamperometric experiments conducted at −0.6 V (Figure 2b), even though there was a positive increase in the current densities (positively) in the last hour of the experiments over time. The choice of the potentials and the 40 mM NaNO_3_ concentration for the chronoamperometric experiments was primarily driven by the LSV responses (Figures 2a and S16). The chronoamperometric experiments were conducted at −0.6 V for the Co_3_O_4_(Cowt %55)GNR catalyst in the presence of different nitrate concentrations (Figure S19) exhibited the most negative current densities, on average, in 3 h of chronoamperometric experiments for the nitrate concentrations of 30 and 40 mM (Figures 2b and S19c-d).

The chronoamperometric current densities recorded for the bare CP and GNR(Cowt %0) are utterly negligible (Figures S20d-e). The chronoamperometric current densities of the bare Co_3_O_4_(Cowt %75) and Co_3_O_4_(Cowt %38)GNR catalysts were quite close (Figures S20b-c). Below is the order of the chronoamperometric current densities recorded for the catalysts: Co_3_O_4_(Cowt %55)GNR > bare Co_3_O_4_(Cowt %75) ∼ Co_3_O_4_(Cowt %38)GNR > bare CP ∼ bare GNR(Cowt %0).

#### Analysis of the Products Derived from NO_3_^–^ Electrochemical Reduction

Figure S21 shows the UV–visible standard curves used to quantify the NO_3_^–^, NO_2_^–^, NH_4_^+^, and N_2_H_4_ products and their respective linear equations; Figure S22 shows the IC standard curves used to quantify the NO_3_^–^, NO_2_^–^, and NH_4_^+^ products and their respective linear equations. The methods used to quantify these products^52−55^ are described in the Supporting Information.

Figures 2d-e and Figure S23 show the UV responses obtained from the quantification of NO_3_^–^ in both the H-cell anodic and cathodic branches before and during the 3 h period of the chronoamperometric experiments conducted using the Co_3_O_4_(Cowt %55)GNR catalyst at different potentials (Figures 2d-e, −0.6 V); the responses obtained point to NO_3_^–^ migration from the cell cathodic branch to the anodic branch, in addition to the consumption of NO_3_^–^. It is worth noting that the NO_3_^–^ concentration was initially placed only in the cathodic branch in all of the experiments. Table S8 shows that, in the absence of an applied potential, the highest NO_3_^–^ migration percentage recorded from the H-cell cathodic branch to the anodic branch was 3.86%, after 3 h of experiment.

Figures 2h and S24a show that at −0.6 and −0.7 V vs RHE, the Co_3_O_4_(Cowt %55)GNR catalyst exhibited NO_3_^–^ conversion efficiency of 14.71 and 15.78%, respectively, with NH_4_^+^ selectivity of 100%; this justifies the choice of the potential of −0.6 V vs RHE as the best potential, based on cost-benefit analysis. We were unable to identify the presence of NO_2_^–^ (via UV absorbance and IC results) and N_2_H_4_ (via visible absorbance results) products in both the cathodic branch and the anodic branch of the cell. NH_4_^+^ was not identified in the anodic branch. The recorded percentage of NO_3_^–^ migration was 20.17% (Figures 2i and S24d), which was a bit higher than the NO_3_^–^ conversion efficiency (Figure 2h) obtained for the Co_3_O_4_(Cowt %55)GNR catalyst at −0.6 V vs RHE. The NO_3_^–^ migration value recorded for the system with applied potential (20.17%) was 5.22 higher in comparison with the value (3.86%, Table S8) recorded for the system without applied potential; this essentially points to the influence of applied potential on NO_3_^–^migration.

We based our mechanism of NH_4_^+^ production following the pathway described by Anastasiadou et al.^24^ in alkaline/neutral medium, as shown in the equations below and confirmed by the in situ FTIR and Raman spectroscopic results and DFT calculations:
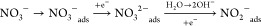
7Continues to produce NH_4_^+^:
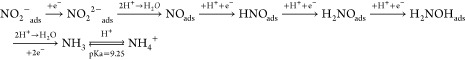
8Figure S25 shows the UV responses obtained for NO_3_^–^ quantification in both the H-cell anodic and cathodic branches before and after 3 h of chronoamperometry experiments conducted using the Co_3_O_4_(Cowt %55)GNR catalyst at different NO_3_^–^ concentrations; once again, the responses showed NO_3_^–^ migration from the cathodic branch to the anodic branch (Figures 2i and S24e), apart from the consumption of NO_3_^–^ (see Figures 2h and S24b). In Figure S24b, one will observe that, at −0.6 V vs RHE, the Co_3_O_4_(Cowt %55)GNR catalyst recorded the highest NO_3_^–^ conversion efficiency (32.77%) in the presence of 10 mM NO_3_^–^, with NH_4_^+^ selectivity of 100% maintained up to 40 mM NO_3_^–^ (on average). After 40 mM NO_3_^–^, NH_4_^+^ selectivity falls drastically to 14.93% at 100 mM NO_3_^–^. NO_3_^–^ migration was also high (24.06%, see Figure S24e) for 10 mM NO_3_^–^; however, as can be noted, an average NO_3_^–^ migration of 17.65% was recorded for the varying NO_3_^–^ concentrations investigated (Figure S24e).

Figure S26 shows the UV responses obtained for NO_3_^–^ quantification in both the H-cell anodic and cathodic branches before and after 3 h of chronoamperometric experiments conducted using different catalysts at 40 mM NO_3_^–^ concentration. The results also show NO_3_^–^ migration from the cathodic branch to the anodic branch, as well as NO_3_^–^ consumption; see a detailed discussion of these results in the Supporting Information.

It is worth noting that in order to determine the aforementioned NH_4_^+^ selectivity, we employed NH_4_^+^ quantification values obtained from the UV–visible curves shown in Figures S27–S29. Also, the IC curves are shown in Figures S30–S33 for the quantification of NO_3_^–^ and NH_4_^+^, we were able to confirm the values presented in Figures 2h-i and S24; indeed, the values obtained from the curves were found to be very close to those presented in Figures 2h-i and S24.

Figure S34 shows the results obtained from the electrochemical experiments conducted using the CP electrode modified with 37.5 μg cm^–2^ Co_3_O_4_(Cowt %55)GNR in Ar–saturated 0.1 M K_2_SO_4_, in the absence of NaNO_3_; NaNO_3_ was not applied in the electrolyte solution because we wanted to show that NH_4_^+^ is derived from NO_3_^–^ and not from other N sources. First, the LSV response is shown in Figure S34a can be found to be very similar to that shown in Figure 2a. Second, the chronoamperometric responses (Figure S34b) are at least three times lower in current densities in comparison with the chronoamperometric responses in Figure 2b. Third, the *R*_ct_ value recorded is 1.5 kΩ (Figure S34c), which is similar to that obtained for the Co_3_O_4_(Cowt %55)GNR catalyst in the presence of varying concentrations of NO_3_^–^ (Table S7). The solution pH ranged from 6.0, before the chronoamperometry experiments, to 12.1, after the experiments. Fourth, we were neither able to detect the presence of NH_4_^+^ (Figures S34d-e) and NO_3_^–^ (Figures S34f-g) in both the H-cell anodic and cathodic branches, nor the presence of hydrazine (Figure S34h) and NO_2_^–^ (Figure S34i) in the cathodic branch.

**Figure 3:**
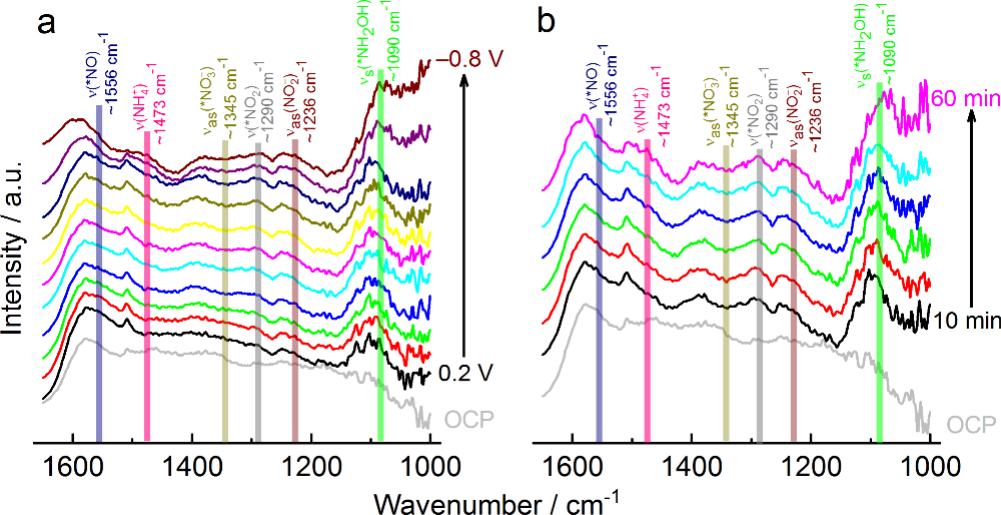
(a) In situ FTIR spectra at different chronoamperometric potentials (difference of 100 mV for each spectrum from 0.2 to–0.8 V vs RHE) and the OCP spectrum; b) in situ FTIR spectra in different times (after 10 min of chronoamperometry at −0.6 V vs RHE for the acquisition of each spectrum) and the OCP spectrum. The Co_3_O_4_(Cowt %55)GNR catalyst (37.5 μg cm^–2^) supported on GC was used as a working electrode in the presence of 40 mM NaNO_3_ and 0.1 M K_2_SO_4_.

Figure S35 shows the results obtained from the electrochemical experiments conducted using the CP electrode modified with 37.5 μg cm^–2^ of Co_3_O_4_(Cowt %55)GNR in Ar-saturated 0.1 M K_2_SO_4_, in the presence of 40 mM NaNO_3_, in both the H-cell anodic and cathodic branches; the NaNO_3_ concentration was applied in order to evaluate the influence of NO_3_^–^ migration in the responses. First, the LSV response (Figure S35a) was lower (current densities) in comparison with that recorded in Figure 2a when 40 mM NaNO_3_ was present initially only in the cell cathodic branch; also, the response was even lower than that recorded in the absence of 40 mM NaNO_3_ (Figure S35a). Second, the chronoamperometric responses (Figure S35b) were found to be at least two times lower in current densities in comparison with the responses presented in Figure 3b. Third, the *R*_ct_ value was 5.0 kΩ (Figure S35c); this value was higher than the values recorded for the other modified electrodes (with the exception of the bare Co_3_O_4_(Cowt %75)) including one electrode which contained varying concentrations of NO_3_^–^ (Table S7). The solution pH ranged from 6.3, before the chronoamperometry experiments, to 12.3, after the experiments. Fourth, we detected some variation in the NO_3_^–^ concentration in the H-cell anodic and cathodic branches (Figures S35d-e).

The amounts of NH_4_^+^ produced were quantified by the increased IC responses shown in Figure S35f for the H-cell cathodic branch. After three h of chronoamperometric experiments, the maximum NO_3_^–^ conversion efficiency and NH_4_^+^ selectivity recorded were 23.3% and 22.0% (Figure S35h), respectively; interestingly, the NO_3_^–^ conversion efficiency value was better while the NH_4_^+^ selectivity value was worse than that recorded in similar experiments conducted when 40 mM NaNO_3_ was initially present only in the H-cell cathodic branch (NO_3_^–^ conversion efficiency of 14.71% and NH_4_^+^ selectivity of 100%, see Figure 2h). NO_3_^–^ migration recorded after 3 h of chronoamperometric experiments was 5.79% (Figure S35i); this value was 3.5 times lower than the value (20.17%)(Figure 2i) obtained from similar experiments conducted when 40 mM NaNO_3_ was initially present only in the H-cell cathodic branch, though it was close to the value recorded for the system operated in the absence of applied potential when 40 mM NaNO_3_ was initially present only in the H-cell cathodic branch (3.86%, Table S8). These results point to the relevance of NO_3_^–^ migration from the electrochemical cell cathodic branch to the anodic branch when it comes to the improvement of the NH_4_^+^ yield rate, as will be discussed below. Equations S1–S3, modified from eqs 3–5, used for quantifying NO_3_^–^ conversion, NH_4_^+^ selectivity, and NO_3_^–^ migration for the analysis conducted with NaNO_3_ present in both the H-cell anodic and cathodic branches, can be found in the Supporting Information.

Finally, Figures 2g (considering IC curves used to quantify NH_4_^+^, Figure 2f) and S36 show the NH_4_^+^ yield rate in mg h^–1^ mg_cat_^–1^ and the FE results obtained for the catalysts investigated in this study. Table S9 also presents the NH_4_^+^ yield rates on other dimensions.

The Co_3_O_4_(Cowt %55)GNR catalyst employed in the presence of 40 mM NO_3_^–^ at −0.60 V vs RHE (Figures 2g and S36a-b) recorded the best NH_4_^+^ yield rate (42.11 mg h^–1^ mg_cat_^–1^); this value is comparable to the values reported in the literature for the best catalysts employed for NO_3_^–^ reduction targeted at NH_4_^+^ production (Table S10; compare also the values of Table S10 with Table S9), considering the catalyst loading of 37.5 μg cm^–2^ applied in our present work. The FE recorded was 98.7%; however, it should be noted that FE values above 96% were obtained only for NO_3_^–^ concentrations in the range of 40–60 mM. FE was found to decrease for NO_3_^–^ concentrations below 40 mM and above 60 mM (Figure S36b). For the cases in which low FE and NH_4_^+^ selectivity are recorded, the NO_3_^–^ mechanism may be changed in order to produce N_2_ as well; this is represented by the addition of the following steps below:

the conversion of NO_3_^–^ to NO_2_^–24^ (eq 7) is followed by a Duca-Feliu-Koper mechanism,^56^ which is aimed at producing N_2_ as well:
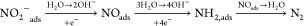
9

or by a Katsounaros-Kyriacou mechanism,^57^ which is aimed at producing N_2_ as well:
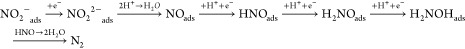
10The other catalysts were unable to reach the high NH_4_^+^ yield rate value recorded for the Co_3_O_4_(Cowt %55)GNR catalyst in the presence of 40 mM NO_3_^–^, at the potential of −0.60 V vs RHE. The bare GNR(Cowt %0) catalyst recorded considerably lower FE, while the bare CP catalyst recorded no FE (Figure S36c).

When applied in the presence of 40 mM NO_3_^–^ in both the H-cell anodic and cathodic branches at −0.60 V vs RHE (Figure S35g), the Co_3_O_4_(Cowt %55)GNR catalyst (Figure S35g) recorded NH_4_^+^ yield rate of 12.09 mg h^–1^mg_cat_^–1^, which was 3.5 times lower than the value recorded for the same catalyst when 40 mM NO_3_^–^ was initially present only in the H-cell cathodic branch at −0.60 V vs RHE (Figure 2g). In addition, the FE recorded when the Co_3_O_4_(Cowt %55)GNR catalyst was applied under the first conditions was 89.7%; this value was also lower than the value obtained when the catalyst was applied, with 40 mM NO_3_^–^ initially present only in the cell cathodic branch, at −0.60 V vs RHE (FE = 98.7%; see Figure 2g). When it comes to NH_4_^+^ production from NO_3_^–^ electrochemical reduction, these results clearly point to the undeniable relevance of the migration of NO_3_^–^ from the H-cell cathodic branch to the anodic branch when NaNO_3_ is present only initially in the H-cell cathodic branch. This could be related to the complex NO_3_^–^ electrochemical reduction reaction involving several adsorbed intermediates, as described in eqs 7–8, which may affect the adsorption of these intermediates, depending on the flow of NO_3_^–^ species near the catalyst surface.

Considering the large range of catalyst loadings used in the literature (35–6400 μg cm^–2^, Table S10), we decided to evaluate the effects of applying 150 (Figure S37), 75 (Figure S38), 56 (Figure S39), and 19 μg cm^–2^ (Figure S40) of Co_3_O_4_(Cowt %55)GNR in the CP electrode (Figure S41) using Ar-saturated 0.1 M K_2_SO_4_ as supporting electrolyte, with 40 mM NaNO_3_ present only in the H-cell cathodic branch. First, the LSV responses (Figures S37a-40a) obtained were lower (current densities) in comparison with the LSV responses presented in Figure 2a. Second, the chronoamperometric responses (Figures S37b-40b) were found to be, on average, two times lower in current densities in comparison with the responses presented in Figure 2b. Third, the *R*_ct_ values (Table S11 and Figures S37c-40c) were higher than the *R*_ct_ value obtained for the CP electrode modified with 37.5 μg cm^–2^ of Co_3_O_4_(Cowt %55)GNR (TableS7 and Figure 2c). The solution pH ranged from 6.9, before the chronoamperometry experiments, to 13.4, after the experiments (Table S11). Fourth, there was variation in the NO_3_^–^ concentration in the H-cell anodic and cathodic branches (Figures S37–40(d-e)).

The amounts of NH_4_^+^ produced were quantified by the increased IC responses shown in Figures S37f-40f for the H-cell cathodic branch. After 3 h of chronoamperometric experiments, the NH_4_^+^ yield rates obtained ranged from 3.85 to 7.92 mg h^–1^ mg_cat_^–1^(with FE ranging from 89.7 to 86%) for the CP electrodes modified with 150 (Figure S37g), 75 (Figure S38g), and 56 μg cm^–2^ (Figure S39g) of Co_3_O_4_(Cowt %55)GNR, while the CP electrode modified with 19 μg cm^–2^ of Co_3_O_4_(Cowt %55)GNR (Figure S40g) recorded NH_4_^+^ yield rate of 30.7 mg h^–1^mg_cat_^–1^ (with FE of 98.4%). These values are lower than the values recorded for the CP electrode modified with 37.5 μg cm^–2^ (Figure 2g) of Co_3_O_4_(Cowt %55)GNR; this result helps further confirm that this loading (37.5 μg cm^–2^) is the best among the loadings investigated when it comes to NO_3_^–^ electroreduction to produce NH_4_^+^. Another finding that deserves being mentioned is that, after three h of chronoamperometric experiments, the NO_3_^–^ conversion efficiency recorded for the CP electrodes modified with 150 (Figure S37h), 75 (Figure S38h), 56 (Figure S39h), and 19 μg cm^–2^ (Figure S40h) of Co_3_O_4_(Cowt %55)GNR ranged from 12.6 to 7.0%, with NH_4_^+^ selectivity ranging from 41.4 to 99.6%; these values are worse than those recorded in similar experiments conducted using the CP electrode modified with 37.5 μg cm^–2^ (Figure 2h) of Co_3_O_4_(Cowt %55)GNR (NO_3_^–^ conversion efficiency of 14.71% and NH_4_^+^ selectivity of 100%, see Figure 2h). NO_3_^–^ migration percentages recorded after 3 h of chronoamperometric experiments were in the range of 3.6 to 14.5% (Figures S37i-40i); these values are lower than the value (20.17%) (Figure 2i) obtained from similar experiments conducted using the CP electrode modified with 37.5 μg cm^–2^ (Figure 2i) of Co_3_O_4_(Cowt %55)GNR. In essence, the results show that when the Co_3_O_4_(Cowt %55)GNR loading is higher than 37.5 μg cm^–2^, there is an increase in the neighboring active sites and NO_ads_ or HNO_ads_ species, and this causes the Feliu-Koper^56^ or Katsounaros-Kyriacou^57^ mechanism to favorably produce N_2_ instead of NH_4_^+^.

Considering that the use of the H-cell separated with sintered glass clearly enabled the migration of NO_3_^–^ from the cathode to the anode chamber, and that the NH_4_^+^ produced (and intermediates) during the NO_3_^–^ electroreduction process most probably also permeated through the sintered glass and migrated to the anode where it is oxidized,^58,59^ all this can lead to some inaccurate numbers when it comes to determining the yield rate of ammonia and FE values; in view of that, an experiment was conducted using a H-cell separated with a Nafion 117 membrane (Figure S42), which is typically used to prevent ion exchange between the two electrodes in the electrolyte. The results obtained from this experiment are shown in Figure S43.

First, the LSV responses (Figures S43a) were lower (current densities) in comparison with those presented in Figure 2a. Second, the chronoamperometric responses (Figures S43b) were found to be, on average, two times lower in current densities in comparison with the responses presented in Figure 2b. Third, the *R*_ct_ value (Table S11 and Figure S43c) was lower than that recorded in the H-cell separated by the sintered glass (Table S7 and Figure 2c). The solution pH ranged from 8.4, before the chronoamperometry experiments, to 13.0, after the experiments (Table S11). Fourth, there was variation in the NO_3_^–^ concentration in the H-cell anodic and cathodic branches (Figures S43(d-e)).

The amounts of NH_4_^+^ produced were quantified by the increased IC responses shown in Figure S43f for the H-cell cathodic branch. After three h of chronoamperometric experiments, the NH_4_^+^ yield rate recorded was 8.75 mg h^–1^mg_cat_^–1^, with FE = 60.7% (Figure S43g). While these values are much lower than the values recorded in the H-cell separated by sintered glass (NH_4_^+^ yield rate of 42.11 mg h^–1^mg_cat_^–1^ and FE of 98.7%, see Figure 2g), they are relatively close to those obtained from the experiment conducted in the H-cell separated by sintered glass, which initially contained NO_3_^–^ in both the anodic and cathodic branches (NH_4_^+^ yield rate of 12.09 mg h^–1^mg_cat_^–1^ and FE of 89.7%, see Figure S35g). Furthermore, after three h of chronoamperometric experiments, the NO_3_^–^ conversion efficiency recorded was 16.1%, with NH_4_^+^ selectivity of 25.5% (Figure S43h). While the NO_3_^–^ conversion efficiency value is relatively higher and the NH_4_^+^ selectivity value is relatively lower compared to the values recorded for a similar experiment conducted in the H-cell separated by sintered glass (NO_3_^–^ conversion efficiency of 14.71% and NH_4_^+^ selectivity of 100%, see Figure 2h), they are close to the values recorded for the experiment conducted in the H-cell separated by sintered glass, which initially contained NO_3_^–^ in both the anodic and cathodic branches (NO_3_^–^ conversion efficiency of 23.3% and NH_4_^+^ selectivity of 22.0% (Figure S35h)). NO_3_^–^ migration recorded after 3 h of chronoamperometric experiments was 4.01% (Figure S43i); while this value is much lower than that obtained in a similar experiment conducted in the H-cell separated by sintered glass (20.17%) (Figure 2i), it is very close to the value obtained from the experiment conducted in the H-cell separated by sintered glass, which initially contained NO_3_^–^ in both the anodic and cathodic branches (NO_3_^–^ migration of 3.77%, see Figure S35i).

It is interesting to note that, despite using the Nafion 117 membrane as separator, there was NO_3_^–^ migration from the cathodic branch to the anodic branch (and probably some NH_4_^+^ migration too) in similar amounts as observed in the experiment conducted using the H-cell separated by sintered glass, where NO_3_^–^ was initially present in both the anodic and cathodic branches; furthermore, relatively close NH_4_^+^ yield rate, FE, NO_3_^–^ conversion, and NH_4_^+^ selectivity values were recorded for both systems. These results point to the immense relevance of NO_3_^–^ migration between the anodic and cathodic branches when it comes to obtaining high values of NH_4_^+^ yield rate and FE, as observed through the application of the Co_3_O_4_(Cowt %55)GNR catalyst. Indeed, the high NH_4_^+^ yield rate and FE values were obtained by using sintered glass as a separator and applying NO_3_^–^ initially only in the cathodic branch; these findings reflect the accuracy of the results described in this work.

#### In-situ FTIR Measurements

To identify the intermediates and products generated during NO_3_^–^ electroreduction, in situ FTIR (Figure S44) measurements were conducted using the Co_3_O_4_(Cowt %55)GNR catalyst (37.5 μg cm^–2^) supported on GC, in the absence (Figure S45) and presence of 40 mM NaNO_3_ (Figure 3). Figure 3a shows the FTIR spectra obtained under different chronoamperometric potentials, taking the OCP spectrum as the reference. As can be observed, there are positive bands (more intense after 0 V in the direction of negative potentials) at around 1090, 1236, 1290, 1473, and 1556 cm^–1^ which correspond to the stretching vibration of adsorbed NH_2_OH (ν_s_(*NH_2_OH)),^60,61^ NO_2_^–^ antisymmetric stretching vibration (ν_as_(NO_2_^–^)),^7,61,62^ vibration of adsorbed NO_2_ (ν(*NO_2_)),^60^ N–H bending vibration of NH_4_^+^ (ν_b_(NH_4_^+^)),^60−62^ and NO adsorbed vibration (ν(*NO)),^60,62^ respectively, and a negative band at around 1345 cm^–1^, which is linked to the NO_3_^–^ asymmetric stretching vibration (ν_as_(NO_3_^–^)).^7,60−62^ In general, there is an increase in the intensity of these peaks when the chronoamperometric time is increased at a potential of −0.6 V (Figure 3b); in addition, the peaks are not observed in the absence of NaNO_3_ in 0.1 M K_2_SO_4_ (Figure S45). The in situ FTIR results helped to definitively show that the action mechanism of nitrate electroreduction in the Co_3_O_4_(Cowt %55)GNR catalyst is exactly as described by Anastasiadou et al.^24^ (eqs 7–8).

#### In Situ Raman Measurements

**Figure 4:**
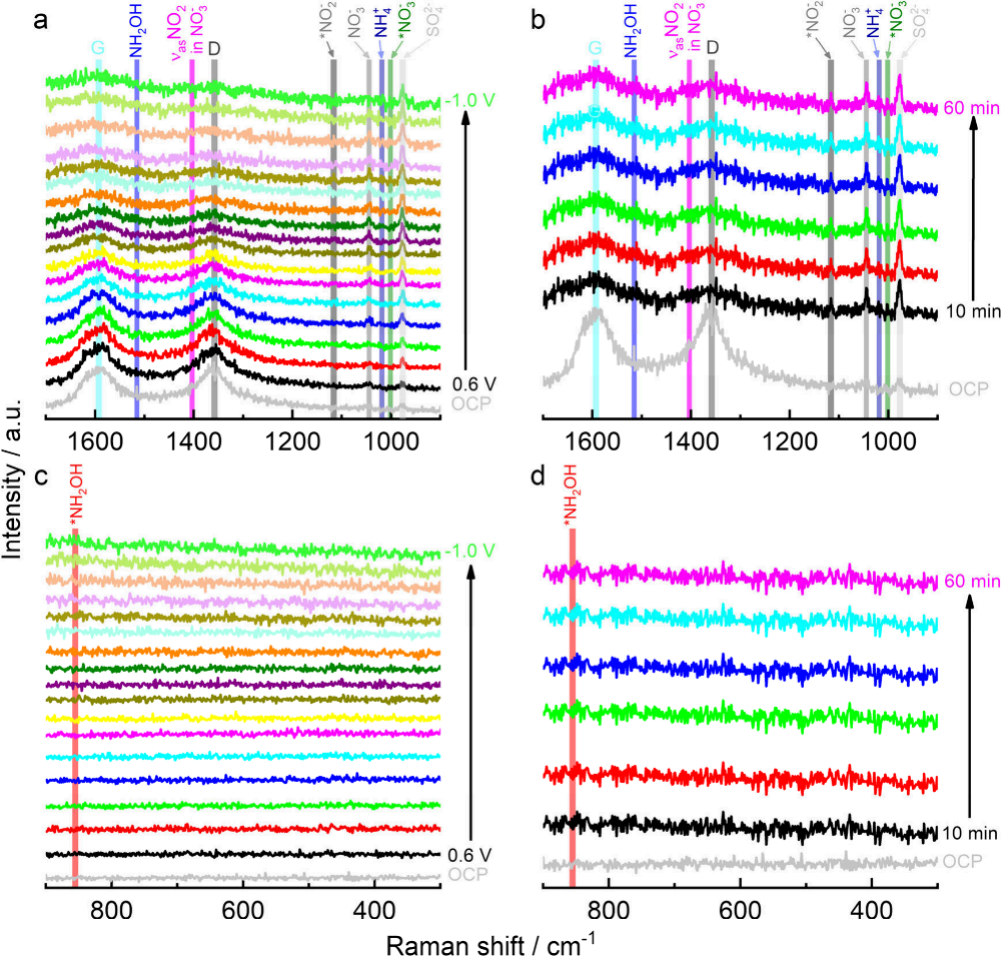
(a and c) In situ Raman spectra at different chronoamperometric potentials (difference of 100 mV for each spectrum from 0.6 to–1.0 V vs RHE) and the OCP spectrum; (b and d) in situ Raman spectra in different times (after 10 min of chronoamperometry at −0.6 V vs RHE for the acquisition of each spectrum) and the OCP spectrum. The carbon-supported Co_3_O_4_(Cowt %55)GNR catalyst (37.5 μg cm^–2^) was used as a working electrode in 0.1 M K_2_SO_4_ in the presence of 40 mM NaNO_3_.

With the application of the carbon-supported Co_3_O_4_(Cowt %55)GNR catalyst (37.5 μg cm^–2^), in the absence (Figure S47) and presence of 40 mM NaNO_3_ (Figure 4), the in situ Raman (Figure S46) measurements were used to identify the intermediates and products that have been generated during the electroreduction of NO_3_^–^. Figures 4a and 4c show the Raman spectra under different chronoamperometric potentials, where the OCP spectrum is taken as a reference. As can be observed, bands can be found at around 977, 1001, 1019, 1045, 1116, 1358, 1402, 1514, and 1593 cm^–1^ corresponding to SO_4_^2–^,^62^ adsorbed *NO_3_^–^ species,^63^ NH_4_^+^,^62^ NO_3_^–^,^62,63^ symmetric stretching vibration of adsorbed NO_2_ in a nitro configuration (*NO_2_^–^),^64^ GNR disorder band (D band), antisymmetric vibrations of NO_2_ group in NO_3_^–^ (ν_as_ NO_2_ in NO_3_^–^),^64^ N–H bending of NH_2_OH,^64^ and GNR graphite band (G band) respectively. The intensity of many of these bands increases at potentials more negative than −0.1 V vs RHE. The band at around 855 cm^–1^ is linked to the N–O stretch mode of surface-adsorbed *NH_2_OH intermediate.^63^ The decreased intensities of GNR D and G bands in potentials more negative than −0.5 V (Figures 4a-b) point to the adsorption of reactants and the formation and desorption of intermediates in different stages of the nitrate electroreduction process. In addition, in general, the intensity of these peaks increases when the chronoamperometric time is increased to −0.6 V (Figures 4b and 4d); also, only bands related to SO_4_^2–^ and GNR D and G are identified in the absence of NaNO_3_ in 0.1 M K_2_SO_4_ (Figure S47). The in situ Raman results also helped definitively confirm that the action mechanism of nitrate electroreduction via the Co_3_O_4_(Cowt %55)GNR catalyst is exactly as described by Anastasiadou et al.^24^ (eqs 7–8).

The in situ Raman responses obtained for the bare carbon-supported Co_3_O_4_(Cowt %75) catalyst (37.5 μg cm^–2^) applied in the presence of 0.1 M K_2_SO_4_ (Figure S48a and d) exhibited only the characteristic Raman peak at around 975 cm^–1^ corresponding to SO_4_^2–^, and at 1365 and 1590 cm^–1^ corresponding to the D and G bands (more visible in the OCP and from the potential of 0.2 to −0.3 V), respectively, for the bare carbon-supported Co_3_O_4_(Cowt %75) catalyst. In the presence of 40 mM NaNO_3_ and 0.1 M K_2_SO_4_ (Figure S48 b and e), there is a visible peak at around 1043 cm^–1^, which is related to NO_3_^–^ species in solution. The in situ Raman responses obtained for the bare carbon-supported GNR(Cowt %0) catalyst (37.5 μg cm^–2^) applied in the presence of 40 mM NaNO_3_ and 0.1 M K_2_SO_4_ (Figure S48c and f) exhibited only the characteristic Raman peaks at around 1367 and 1587 cm^–1^ corresponding to the D and G bands, respectively, masking even the Raman peaks related to NO_3_^–^ and SO_4_^2–^ species. These responses clearly reinforce the electrocatalytic effectiveness of the Co_3_O_4_(Cowt %55)GNR catalyst when applied in nitrate reduction to produce ammonium, revealed through the intermediates (Figure 4), as suggested by Anastasiadou et al.^24^ (eqs 7–8).

#### NH_3_ Production Mechanism from DFT Calculations

Here, we investigated the mechanism involving NH_3_ production using DFT calculations, taking into account the intermediates and products identified via the in situ FTIR and Raman spectroscopy analyses, and which are also in line with the mechanism suggested by Anastasiadou et al.^24^ (eqs 7–8). The stepwise reaction process and the Gibbs free energy changes (Δ*G*)^65,66^ for each reaction step were calculated using the expressions outlined in the Supporting Information.

**Figure 5:**
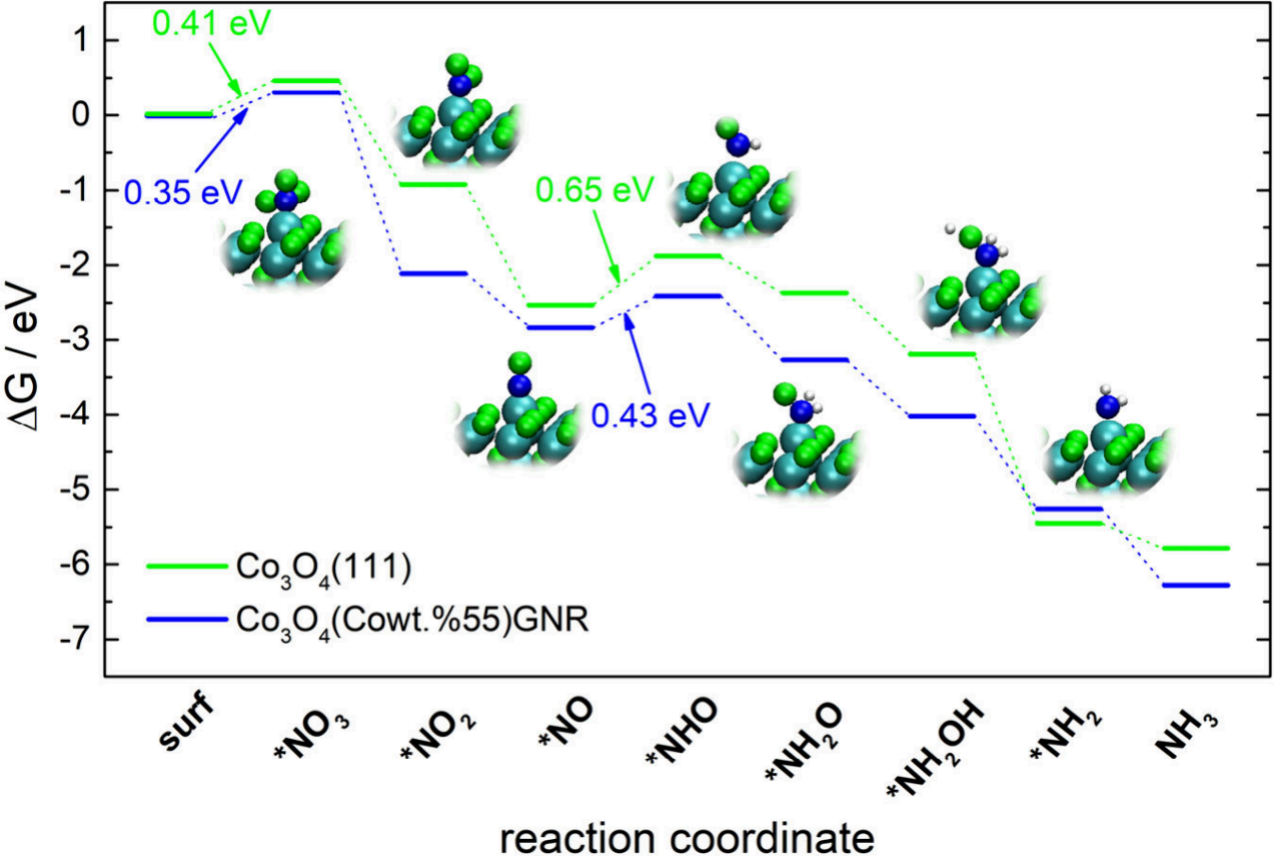
Free energy diagram for the production of NH_3_. Color of the atoms of the internal structures: dark cyan = Co, green = O, blue = N, and white = H.

The NH_3_ production diagram is shown in Figure 5, with representative potentials taken at *U* = 0 V. In both calculated systems, with and without the GNR, the first step, corresponding to the adsorption of NO_3_, involves a high energy. The RDS in the process involves the addition of the first hydrogen atom to NO, corresponding to an energy of 0.65 eV in the absence of GNR and 0.43 eV in the presence of GNR; this is in line with the Δ*G*_4_ step. It is worth noting that only the Δ*G*_7_ step is found to be more favorable in the system without GNR. For all the other steps, the presence of GNR plays a significant role in facilitating ammonia production; essentially, this confirms the importance of the entanglement between Co_3_O_4_ and GNR for the Co_3_O_4_(Cowt %55)GNR catalyst. The DFT results are in line with the results reported in the literature for other electrocatalysts used for nitrate reduction.^60,67^ It should be noted that while some authors use the adsorption free energy to construct the energy diagram for the nitrate reduction mechanism,^68−70^ in the present study, we have employed the reaction free energy (Δ*G*_1_ to Δ*G*_8_ equations in the SI) to construct the diagram (Figure 5).

The first step—adsorption of NO_3_—which involves high energy, and the RDS in the nitrate electroreduction process, which involves the addition of the first hydrogen atom to NO (Figure 5), can be linked to the effective migration of NO_3_^–^ from the H-cell cathodic branch to the anodic branch, which favors the species adsorption on the catalyst surface during the electrochemical reduction of NO_3_^–^ aiming at the production of NH_4_^+^.

## Conclusion

Among the electrocatalysts investigated in this study, the Co_3_O_4_(Cowt %55)GNR catalyst exhibited the best results when applied for nitrate electroreduction to produce NH_4_^+^. With the application of only 37.5 μg cm^–2^ of the catalyst (20.6 μg cm^–2^ of Co), the sample recorded a NH_4_^+^ yield rate of 42.11 mg h^–1^mg_cat_^–1^, FE of 98.7%, NO_3_^–^ conversion efficiency of 14.71%, and NH_4_^+^ selectivity of 100%; this was confirmed through the analysis of catalyst loadings ranging from 19 to 150 μg cm^–2^. The outstanding results obtained by the Co_3_O_4_(Cowt %55)GNR catalyst were favored by the following factors: high average values of ECSA and low values of *R*_ct_; highest entanglement involving Co_3_O_4_ and GNR, and a highly more effective occurrence of the (Co_3_(Co(CN)_6_)_2_(H_2_O)_12_)_1.333_ complex-like structure; the effective migration of NO_3_^–^ from the electrochemical cell cathodic branch to the anodic branch, which was confirmed by the experiment conducted in a H-cell separated by a Nafion 117 membrane—this appeared to favor the species adsorption on the catalyst surface—species involved in the complex reaction of NO_3_^–^ electrochemical reduction to produce NH_4_^+^; and the electrochemical stability of the Co_3_O_4_(Cowt %55)GNR catalyst. The in situ FTIR and Raman results and the DFT calculations helped confirm the presence of the adsorbed intermediates NO_3_^–^, NO_2_^–^, NO, and NH_2_OH and the final product NH_4_^+^ derived from NO_3_^–^ electroreduction, with the NO adsorbed intermediate presenting low energy transition (0.43 eV for the RDS) to the next intermediate (*NO to *HNO) for the Co_3_O_4_(Cowt %53)GNR catalyst in comparison with the Co_3_O_4_(Cowt %75) catalyst (0.65 eV for the RDS).

## Supporting Information Available

Experimental details, figures, and tables related to the supplementary results (PDF)
