# Supplementary material for: Effective Nitrate Electroconversion to Ammonia Using an Entangled Co3O4/Graphene Nanoribbon Catalyst
Source: ACS Appl Mater Interfaces. 2024 Dec 27;17(1):1295–310. doi: 10.1021/acsami.4c18269 (PMC11783537; doi:10.1021/acsami.4c18269)
Supplement: Supplementary file 1 — am4c18269_si_001.pdf [file am4c18269_si_001.pdf]

Supporting Information

Effective nitrate electroconversion to ammonia using an entangled Co_3O_4/graphene nanoribbon catalyst

Marciélli K. R. Souza[a], Eduardo S. F. Cardoso[a,b], Leandro M. C. Pinto[a], Isabela S. C. Crivelli[a], Clauber D. Rodrigues[c], Robson S. Souto[b], Ary T. Rezende-Filho[d], Marcos R. V. Lanza[b], Gilberto Maia[a]*

[a]Institute of Chemistry, Federal University of Mato Grosso do Sul, Av. Senador Filinto Muller 1555, Campo Grande, MS 79074-460, Brazil

[b]São Carlos Institute of Chemistry, University of São Paulo, Avenida Trabalhador São-Carlense 400, São Carlos, SP 13566-590, Brazil

[c]State University of Mato Grosso do Sul; Rua Rogério Luis Rodrigues s/n, Glória de Dourados, MS 79730-000, Brazil

[d]Faculty of Engineering, Architecture and Urbanism, and Geography, Federal University of Mato Grosso do Sul, Av. Costa e Silva, s/nº, Campo Grande, MS 79070-900, Brazil

*Corresponding author:
gilberto.maia@ufms.br (G. Maia)

Synthesis of Graphene Nanoribbon (GNR) [1]

Initially, 0.70 g of MWCNT was dispersed in 20 mL of concentrated H_2SO_4; 0.37 g of $K_2S_2O_8$ and 0.37 g of P_2O_5 were added into the mixture, and the solution was heated at 80 °C for 6 h under stirring. After that, the solutions were then cooled to room temperature and 0 °C water was added therein. The product was filtered under vacuum using a 0.22-µm Nylon membrane and was washed with water to obtain a neutral pH. The product was dried at room temperature.

The product obtained - described in the previous paragraph, was re-oxidized using 40 mL of concentrated H_2SO_4 containing 0.70 g of $NaNO_3$ and 2.10 mg of $KMnO_4$ under stirring, with the solution kept at 0 °C. The mixture was kept at 35 °C for 2 h under stirring, and 320 mL of ultrapure water of 5°C was slowly added, followed by the drop by drop addition of 40 mL of 30 % H_2O_2; the mixture was then stirred for another 20 minutes. The solution was cooled to room temperature and transferred to an ultrapure water 3.5 L container at 0 °C. After 24 hours, the precipitate was centrifuged at 7500 rpm and washed with ultrapure water, and this was followed by washing the material with 90:10 H_2O/HCl v/v solution and then with water until a neutral pH was obtained (the supernatant). The resulting material was subjected to drying at 50°C for 24 hours, and this gave rise to graphene oxide nanoribbons (GONR).

To obtain the GNR, a homogeneous aqueous dispersion composed of 250 mg of GONR, 5.0 mL of hydrazine sulfate (21 mg) solution, and 35 µL ammonium hydroxide solution (28 % by mass % in water) was vigorously shaken in a flask for 30 minutes. The flask was then left in a water bath (95 °C) for 2 h and 30 min. Subsequently, at room temperature, the GNR was obtained after vacuum filtration using a 0.22 µm nylon film and washing with 100 mL of 0.5% (v/v) ammonium hydroxide. The excess of ammonium hydroxide was removed by washing the mixture with ultrapure water until a neutral pH was obtained; the final product obtained (GNR) was dried under vacuum at room temperature.

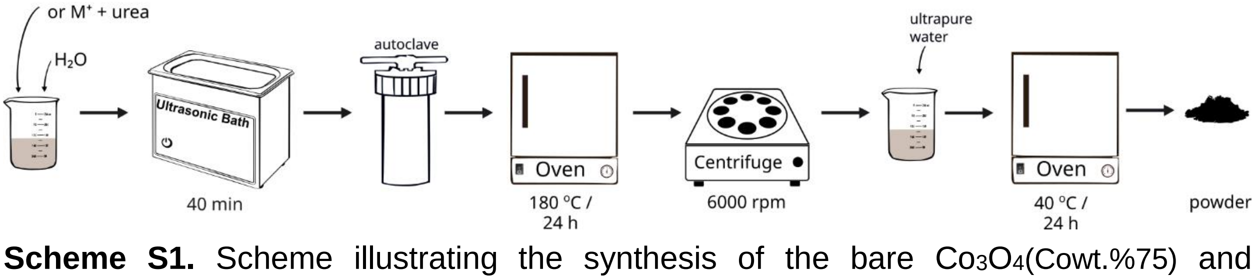

Scheme S1. Scheme illustrating the synthesis of the bare Co_3O_4(Cowt.%75) and Co_3O_4/GNR samples.

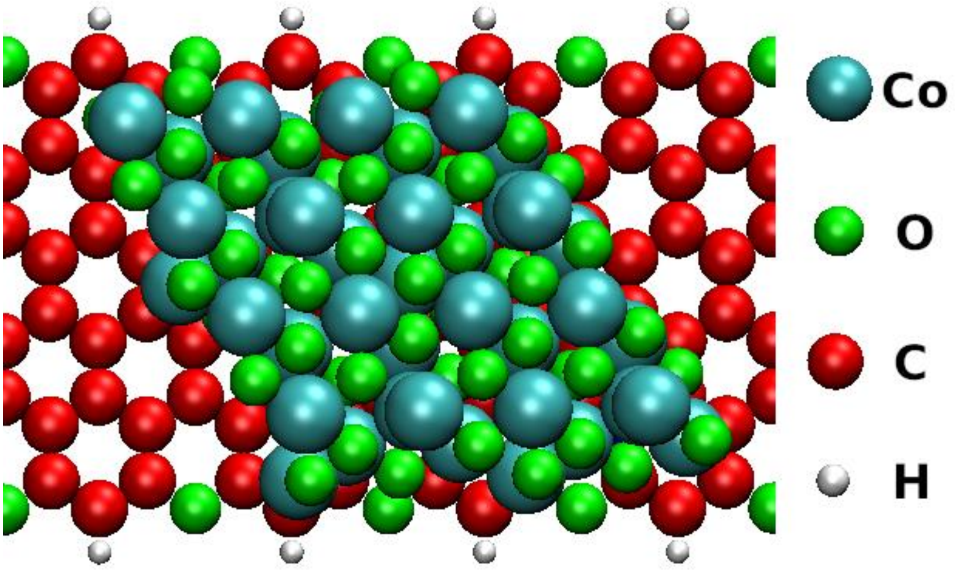

Figure S1. Co_3O_4(111)(Cowt.%55)GNR representation after full geometry optimization.

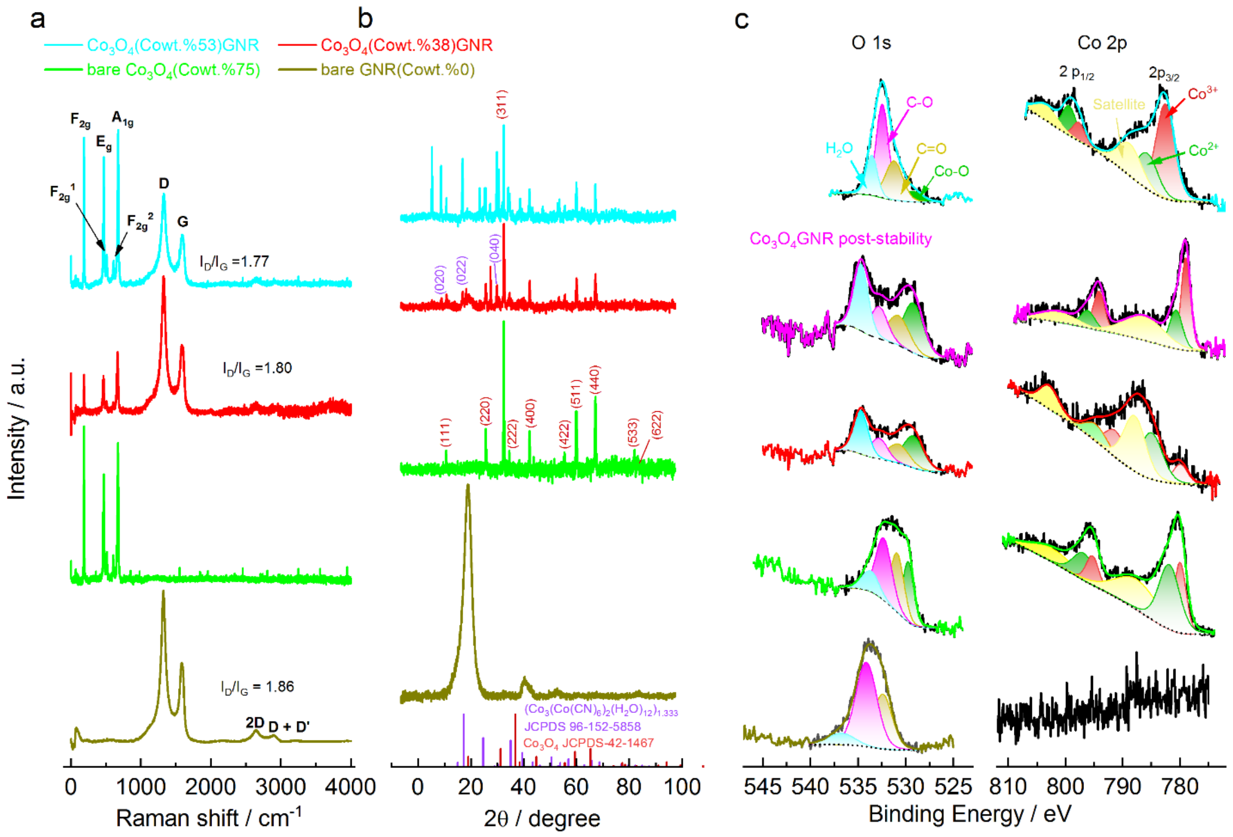

Figure S2. a) Raman spectra; **b**) XRD spectra; and **c**) O 1s and Co 2p HR-XPS spectra for the bare GNR and Co_3O_4, as well as for the different Co_3O_4/GNR samples, and the Co_3O_4GNR post-stability sample (Co_3O_4GNR post-stability sample is the Co_3O_4(Cowt.%55)GNR sample that has been subjected to three hours of chronoamperometric experiment at –0.6 V vs. RHE).

The results obtained from the XRD analyses (Figure S2b) show that the bare GNR(Cowt.%0) exhibits a pronounced peak at 2θ= 25.8° (0.35 nm), which is typically characteristic of the crystalline peak corresponding to the theoretical C graphite with (110) plane (JCPDS 89-8489), and a small peak at 2θ= 43.3° (0.21 nm) related to the (201) plane (JCPDS 89-8489) [2].

The bare Co_3O_4(Cowt.%75) sample exhibits a prominent peak at 2θ = 36.9° (0.24 nm), which is typically characteristic of crystalline peak, corresponding to the Co_3O_4 with (311) plane (JCPDS 42-1467) and other peaks at 2θ of 19, 31.3, 38.3, 44.9, 55.6, 59.4, 65.3, 77.3 and 78.4° (0.46, 0.28, 0.23, 0.20, 0.17, 0.16, 0.14,0.123 and 0.122 nm, respectively), corresponding to the (111), (220), (222), (400), (422), (511), (440), (533) and (622) planes, respectively, which are typically associated with the Co_3O_4 (JCPDS 42-1467); in essence, this result confirms that the Co oxide present in the bare Co_3O_4(Cowt.%75) sample is Co_3O_4.

The XPS survey spectra (Figure S3) show the presence of C 1s and O 1s peaks at 285 and 534 eV, respectively (Table S1), and O KLL and C KLL peaks at 750 and 980 eV, respectively, for the bare GNR(Cowt.%0). For the bare Co_3O_4(Cowt.%75) sample (Figure S3), we also identified the presence of the O 2s, Co 3p, Co 3s, Co LMM, and Co 2p (Table S1) peaks at 28, 62, 103, 483, and 780 (Table S1) eV, respectively; the presence of C 1s and C KLL peaks in the bare Co_3O_4(Cowt.%75) sample (Figure S3) is associated with the fact that the sample was supported by carbon tape when it was subjected to XPS analysis.

The XPS survey spectra (Figure S3) obtained for the Co_3O_4/GNR samples exhibited the same peaks (some less evident) that have already been recorded for the bare GNR(Cowt.%0) and Co_3O_4(Cowt.%75) samples, with the exception of the Co_3O_4GNR post-stability sample which also presented the F 1s peak at 688 eV, corresponding to the Nafion® used to support this sample on the carbon surface - this system was used for the XPS analysis of this sample.

The bare GNR(Cowt.%0) sample exhibited a higher proportion of carbon (wt.%) on its surface (86.5%, wt.), whereas the Co_3O_4(Cowt.%53)GNR sample exhibited a lower proportion of carbon (Table S1) due to the exposure of carbon tape during the analysis of the bare Co_3O_4(Cowt.%75); this resulted in the presence of much higher amount of Co in the Co_3O_4(Cowt.%53)GNR sample in comparison with that observed in

the bare Co_3O_4(Cowt.%75) sample (35 to 26.7 wt.%, Table S1). The amount of Co on the surface of the Co_3O_4(Cowt.%55)GNR sample is also found to be low when compared to Co_3O_4(Cowt.%38)GNR (7.6 to 12.9 wt.%, Table S1). The high amount of Co observed in the Co_3O_4GNR post-stability sample in comparison with that recorded in the Co_3O_4(Cowt.%55)GNR sample (16.8 to 7.6 wt.%, Table S1) is attributed to the fact that a loading of 1,000 µg cm^{-2} was employed in the former (Co_3O_4GNR post-stability sample). As expected, the bare Co_3O_4(Cowt.%75) sample exhibited the highest oxygen content on its surface, followed by the Co_3O_4(Cowt.%53)GNR sample, and the Co_3O_4(Cowt.%38)GNR sample (Table S1). Compared to the Co_3O_4(Cowt.%55)GNR sample, the relatively lower amount of oxygen observed in the Co_3O_4GNR post-stability sample (Table S1) may indicate the occurrence of some corrosion of Co during the stability test.

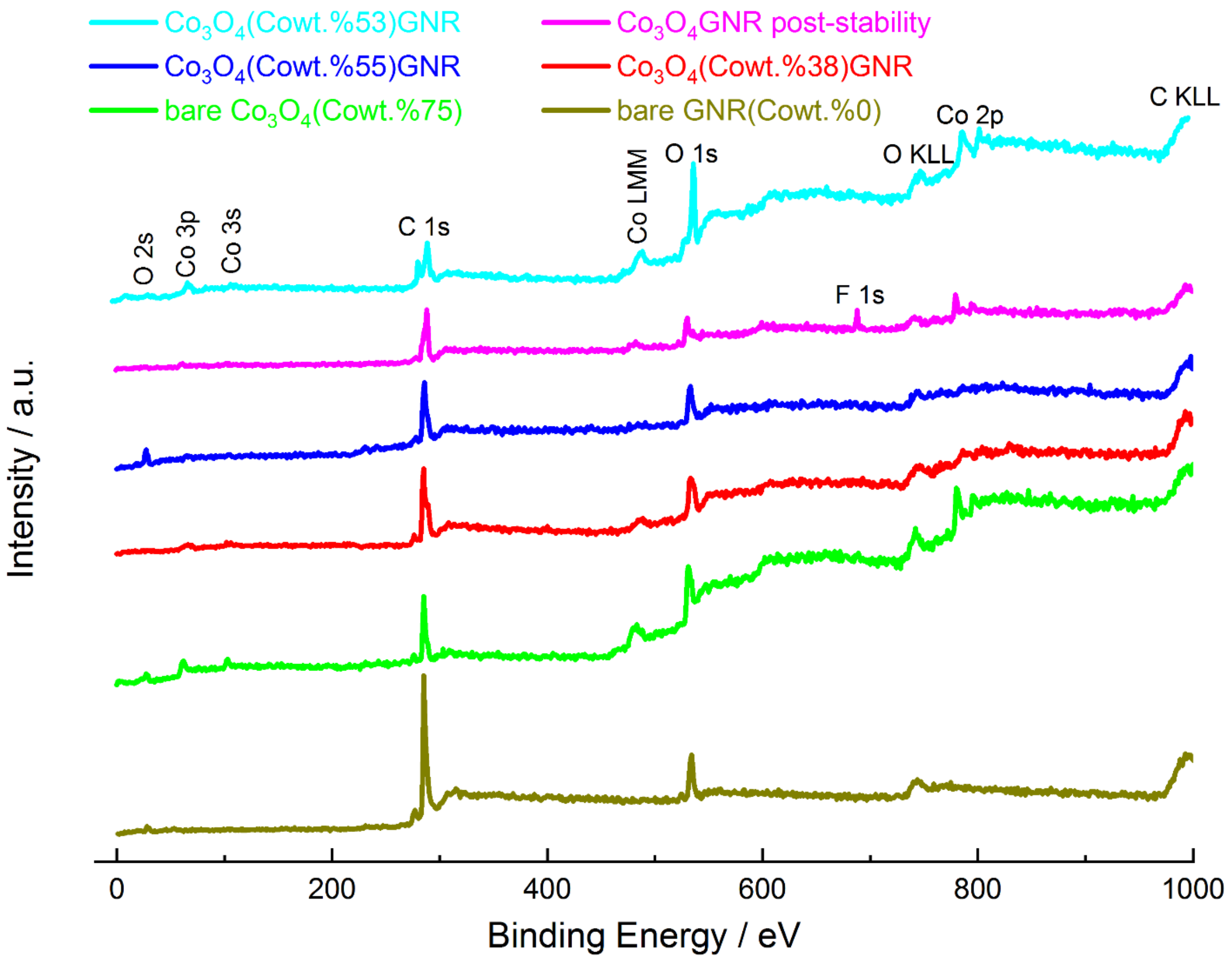

Figure S3. XPS survey spectra obtained for the bare GNR(Cowt.%0) and Co_3O_4(Cowt.%75), as well as for the different Co_3O_4/GNR and Co_3O_4GNR post-stability samples investigated in this study.

Table S1. Positions, relative sensitivity factors (R.S.F.), and atomic and mass percentages obtained from the XPS survey spectra shown in Figure S3 for the bare GNR(Cowt.%0) and Co_3O_4(Cowt.%75), as well as for the different Co_3O_4/GNR and Co_3O_4GNR post-stability samples investigated in this study.

Catalyst	Name	Position (eV)	R.S.F.	Content (at. %)	Content (mass %)
**Co_3O_4(Cowt.%53)GNR**	C 1s	285.0	1.0	57.7	37.7
	O 1s	532.0	2.85	31.4	27.3
	Co 2p	781.0	18.48	10.9	35.0
**Co_3O_4GNR post-stability**	C 1s	288.0	1.0	78.2	64.0
	O 1s	530.0	2.85	17.6	19.2
	Co 2p	779.0	18.48	4.2	16.8
**Co_3O_4(Cowt.%55)GNR**	C 1s	286.0	1.0	79.5	70.3
	O 1s	533.0	2.85	18.7	22.1
	Co 2p	786.0	18.48	1.8	7.6
**Co_3O_4(Cowt.%38)GNR**	C 1s	285.0	1.0	77.5	65.3
	O 1s	533.0	2.85	19.4	21.8
	Co 2p	786.0	18.48	3.1	12.9
**bare Co_3O_4(Cowt.%75)**	C 1s	285.0	1.0	62.6	44.9
	O 1s	531.0	2.85	29.8	28.5
	Co 2p	780.0	18.48	7.6	26.7
**bare GNR(Cowt.%0)**	C 1s	285.00	1.0	89.5	86.5
	O 1s	534.00	2.85	10.5	13.5

The results obtained from the elemental analyses (Table S2) showed that the contents of carbon and oxygen in the GNR(Cowt.%0) sample were quite close to those recorded in the XPS analyses (Table S1). The content of oxygen recorded was only 8.2 wt.%, with some type of Co oxide ('others') coming close to 92% (Table S2) for the bare Co_3O_4(Cowt.%75) sample. The Co_3O_4(Cowt.%55)GNR sample recorded a slightly higher carbon content compared to the Co_3O_4(Cowt.%38)GNR (44.8 to 40.6 wt.%, Table S2) sample, while the Co_3O_4(Cowt.%53)GNR sample recorded a significantly lower carbon content compared to the two samples (21.7 wt.%, Table S2). A similar tendency is also observed in the XPS survey results (Table S1); the values recorded for oxygen content in the elemental analysis (Table S2) were found to be quite close to those obtained in the XPS survey (Table S1). When it comes to attributing the content of 'others' to some type of Co oxide (Table S2), one will observe that the Co_3O_4(Cowt.%53)GNR sample exhibits a much higher content (49.1 wt.%) compared to the Co_3O_4(Cowt.%55)GNR and Co_3O_4(Cowt.%38)GNR samples (around 30.5 wt.%) (Table S2).

It is worth noting that for samples containing GNR, we identified the presence of a small amount of N (Table S2), where the amount of this element was found to decrease from Co_3O_4(Cowt.%38)GNR (1.36 wt.%) to Co_3O_4(Cowt.%53)GNR (0.47 wt.%) sample (equal to that of the bare GNR(Cowt.%0)); this outcome corroborates the occurrence of some bonding between Co and N, as detected in the XRD results (Figure 1b).

Table S2. Elemental analyses of the bare GNR and Co_3O_4, and of the different Co_3O_4/GNR samples investigated.

Catalyst	N (wt.%)	C (wt.%)	H (wt.%)	S (wt.%)	O (wt.%)	Others(wt.%)
**Co_3O_4(Cowt.%53)GNR**	0.47	21.7	0.75	-	28.0	49.1
**Co_3O_4(Cowt.%55)GNR**	1.17	44.8	0.58	-	23.4	30.1
**Co_3O_4(Cowt.%38)GNR**	1.36	40.6	0.55	-	26.5	31.0
**bare Co_3O_4(Cowt.%75)**	-	-	-	-	8.2	91.8
**bare GNR(Cowt.%0)**	0.47	80.0	0.63	-	10.1	8.8

The mass loss observed in the TG response for the bare GNR(Cowt.%0) sample – nearly 100% (Figure S4), was similar to that previously reported in the literature [1,2]. The bare Co_3O_4(Cowt.%75) sample recorded some gain in mass with the increase in temperature due to some gain in oxygen (Figure S4). For the Co_3O_4/GNR samples, the mass loss recorded is found to be accentuated at around 350 °C; this is attributed to the burning of GNR, which occurs hundreds of degrees lower than the observed in bare GNR due to the metal oxide present in the samples [3]. An increase in mass loss is observed from the Co_3O_4(Cowt.%53)GNR (~30%) sample to the Co_3O_4(Cowt.%38)GNR (~50%) (Figure S4) sample; this essentially shows that the content of Co_3O_4 in the Co_3O_4(Cowt.%53)GNR sample is around 70%, ~65% in the Co_3O_4(Cowt.%55)GNR, and ~50% in the Co_3O_4(Cowt.%38)GNR sample.

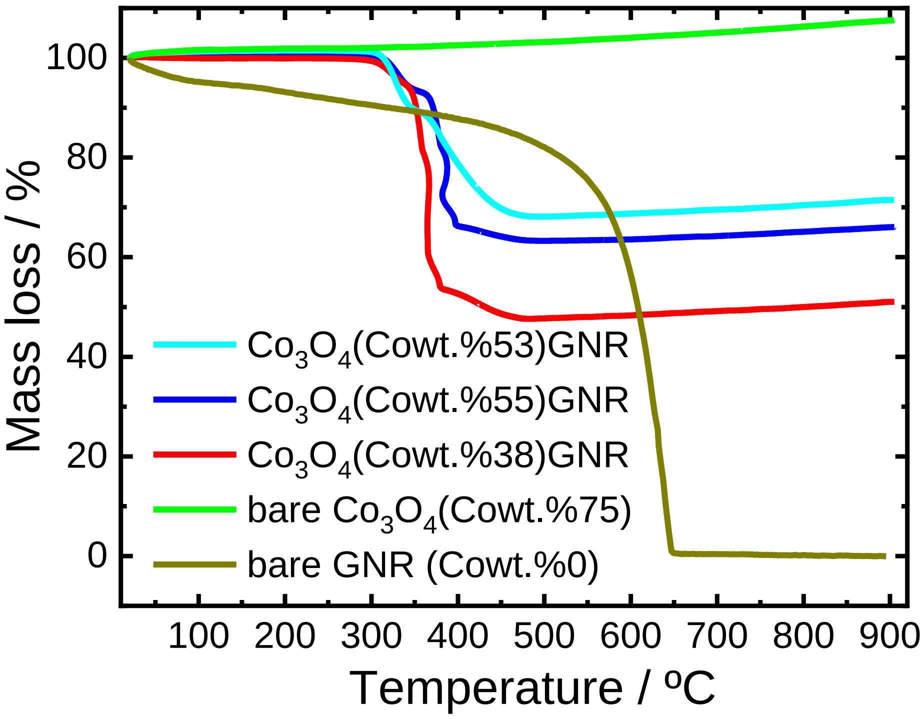

Figure S4. TG curves obtained for the bare GNR(Cowt.%0) and Co_3O_4(Cowt.%75), and for the different Co_3O_4/GNR samples investigated.

Another point that is worth mentioning here is that, considering that, theoretically, the amount of Co in Co_3O_4 is ~74%, by applying this value (74%) in the TG mass loss data, the amount of Co in the Co_3O_4(Cowt.%53)GNR will be around 52%, ~48.1% in the Co_3O_4(Cowt.%55)GNR, and ~37% in the Co_3O_4(Cowt.%38)GNR sample; this clearly points to a close proximity between the results obtained from the AAS analysis (Table S3) and those recorded in the TG analysis. The significant difference in terms of the values recorded between the two techniques (AAS and TGA) for the Co_3O_4(Cowt.%55)GNR sample may be attributed to the presence of a relatively higher amount of '$(Co_3(Co(CN)_6)_2(H_2O)_{12})_{1.333}$ complex' in this sample, which has been identified through the XRD analysis (Figure 1b).

Table S3. AAS results obtained for the bare GNR(Cowt.%0) and Co_3O_4(Cowt.%75), and for the different Co_3O_4/GNR samples investigated.

Catalyst	Aliquot (mL)	Absorbance	Concentration (mg L^{-1})	Cowt.%
Co_3O_4(Cowt.%53)GNR	0.50	0.4469	5.3	53
Co_3O_4(Cowt.%55)GNR	0.50	0.4587	5.5	55
Co_3O_4(Cowt.%38)GNR	0.50	0.3345	3.8	38
bare	0.50	0.5301	7.5	75

Co_3O_4(Cowt.%75)				
bare GNR(Cowt.%0)	0.50	0.0128	0.0	0

For the bare GNR(Cowt.%0) sample (Figure S2c), there were three deconvoluted peaks attributed to the chemical states of C=O, C–O, and H_2O, and positioned at 532.4, 534.1, and 536.7 eV, respectively (Table S4) [1,2].

Table S4. Positions and percentages of the content of functional groups or chemical states present in the bare GNR(Cowt.%0) and Co_3O_4(Cowt.%75), as well as in the different Co_3O_4/GNR and Co_3O_4GNR post-stability catalysts investigated, obtained from high–resolution XPS spectra presented in Figures 1c, S2c, and S5.

Catalyst	Name	Chemical state	Position (eV)	% content
**Co_3O_4(Cowt.%53)GNR**	O 1s	Co–O	529.2	7.3
		C=O	531.2	27.1
		C–O	532.5	47.8
		H_2O	533.6	17.8
	C 1s	C=C & C-C	282.7	20.3
		C–OH & C–O–C	284.4	31.8
		C=O &COOH	285.5	28.5
		π–π	286.3	19.4
	Co 2p	Co(III)$2p_{3/2}$	782.6	41.2
		Co(II)$2p_{3/2}$	785.7	17.7
		satellite	788.8	17.8
		Co(III)$2p_{1/2}$	797.6	5.3
		Co(II)$2p_{1/2}$	799.4	9.9
		satellite	803.4	8.1
**Co_3O_4GNRpost-stability**	O 1s	Co–O	529.2	31.4
		$Co(OH)_2$	531.2	20.1
		C–O	532.5	17.3
		H_2O	533.6	31.2
	C 1s	C=C & C-C	285.2	32.7
		C–OH & C–O–C	287.4	41.9
		C=O &COOH	288.2	25.4
	Co 2p	Co(III)$2p_{3/2}$	779.0	25.4
		Co(II)$2p_{3/2}$	780.7	14.6
		satellite	786.2	28.4
		Co(III)$2p_{1/2}$	794.2	13.0
		Co(II)$2p_{1/2}$	796.1	7.3

		satellite	801.3	11.3
**Co_3O_4(Cowt.%55)GNR**	O 1s	Co–O	530.8	26.5
		C=O	532.5	37.4
		C–O	534.1	22.7
		H_2O	536.2	13.4
	C 1s	C=C & C-C	284.8	32.9
		C–OH & C–O–C	285.9	30.0
		C=O & COOH	287.3	25.6
		π–π	289.2	11.5
	Co 2p	Co(III)$2p_{3/2}$	780.0	13.7
		Co(II)$2p_{3/2}$	783.4	16.8
		satellite	786.3	33.5
		Co(III)$2p_{1/2}$	789.9	21.4
		Co(II)$2p_{1/2}$	794.8	6.9
		satellite	802.0	7.7
**Co_3O_4(Cowt.%38)GNR**	O 1s	Co–O	529.5	2.4
		C=O	532.5	33.3
		C–O	534.3	35.7
		H_2O	536.8	28.6
	C 1s	C=C & C-C	284.3	28.1
		C–OH & C–O–C	285.7	37.4
		C=O & COOH	288.3	18.8
		π–π	289.7	15.7
	Co 2p	Co(III)$2p_{3/2}$	779.9	7.5
		Co(II)$2p_{3/2}$	785.0	26.5
		satellite	787.9	31.2
		Co(III)$2p_{1/2}$	791.3	14.3
		Co(II)$2p_{1/2}$	794.7	9.4
		satellite	802.9	11.1
**bare Co_3O_4(Cowt.%75)**	O 1s	Co–O	529.7	22.9
		C=O	530.9	27.9
		C–O	532.3	36.4
		H_2O	533.7	12.8
	C 1s	C=C & C-C	284.9	46.0
		C–OH & C–O–C	286.2	38.8
		C=O & COOH	288.3	11.9
		π–π	290.1	3.3
	Co 2p	Co(III)$2p_{3/2}$	780.0	16.7
		Co(II)$2p_{3/2}$	781.9	26.9
		satellite	787.5	26.1
		Co(III)$2p_{1/2}$	795.3	7.5
		Co(II)$2p_{1/2}$	796.8	11.4

		satellite	802.2	11.4
**bare GNR(Cowt.%0)**	O 1s	C=O	532.4	33.1
		C–O	534.1	57.2
		H_2O	536.7	9.7
	C 1s	C=C & C-C	285.0	39.0
		C–OH & C–O–C	286.2	26.4
		C=O & COOH	287.6	20.8
		π–π	289.4	13.8

Figure S5 shows the C 1s HR-XPS spectra obtained for the bare GNR(Cowt.%0) and Co_3O_4(Cowt.%75), and for the different Co_3O_4/GNR and Co_3O_4GNR post-stability catalysts investigated in this study; in general, the spectra exhibited a broad peak with shoulder or two peaks, which were deconvoluted into four peaks, attributed to the chemical states C=C & C–C, C–OH & C–O–C, C=O & COOH, and π–π positioned on average at 284.5, 286.0, 287.5, and 289.0 eV, respectively [1,2,4](Table S4). It is worth noting that, one is unable to distinguish the C 1s HR-XPS of the carbon tape or carbon paper, used to support the samples during the XPS analysis, from the C 1s HR-XPS of the GNR(Cowt.%0) sample; this explains why the bare Co_3O_4(Cowt.%75) exhibits the C 1s HR-XPS response. The main content percentages recorded were 33.2 and 34.4%, on average, for the C=C & C–C and C–OH & C–O–C chemical states, respectively (Table S4).

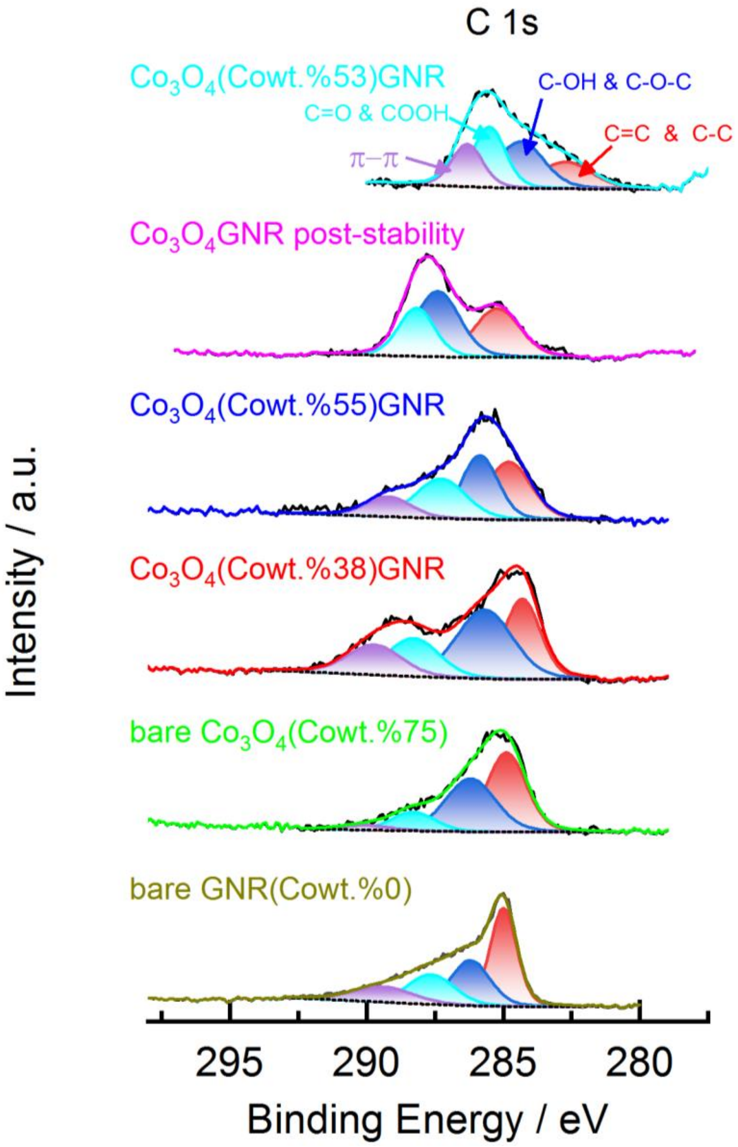

Figure S5. HR-XPS curves obtained for the bare GNR(Cowt.%0) and Co_3O_4(Cowt.%75), and for the different Co_3O_4/GNR and Co_3O_4GNR post-stability samples investigated in this study.

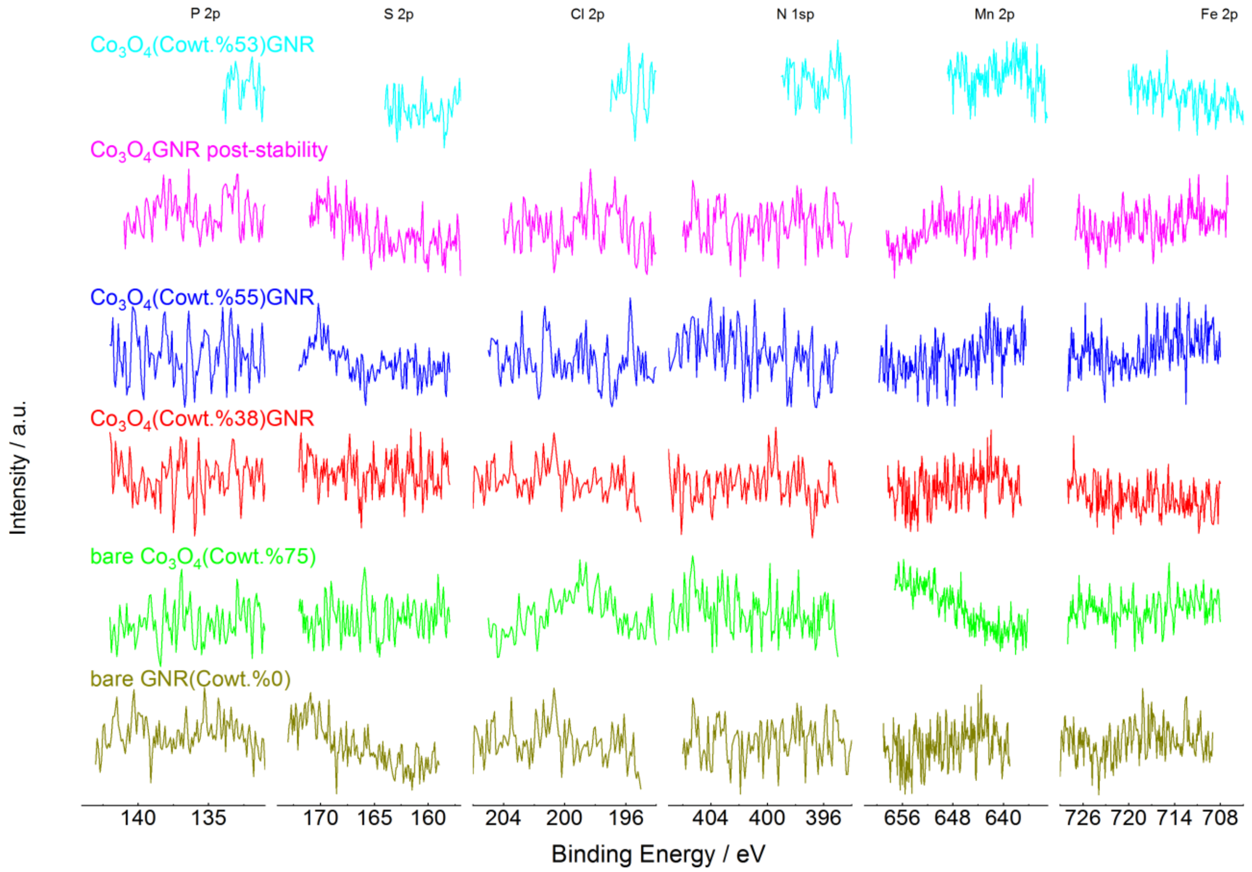

Figure S6. HR-XPS curves obtained for the bare GNR(Cowt.%0) and Co_3O_4(Cowt.%75), and for the different Co_3O_4/GNR and Co_3O_4GNR post-stability samples investigated in this study.

The SEM images in Figure S7 show the expected nanoribbon structures of the bare GNR and bipyramidal, pyramidal, plates, and other structures of the bare Co_3O_4(Cowt.%75) sample. For the Co_3O_4(Cowt.%38)GNR sample, one can clearly see the pyramidal and plates of Co_3O_4 surrounded by or on top of GNR.

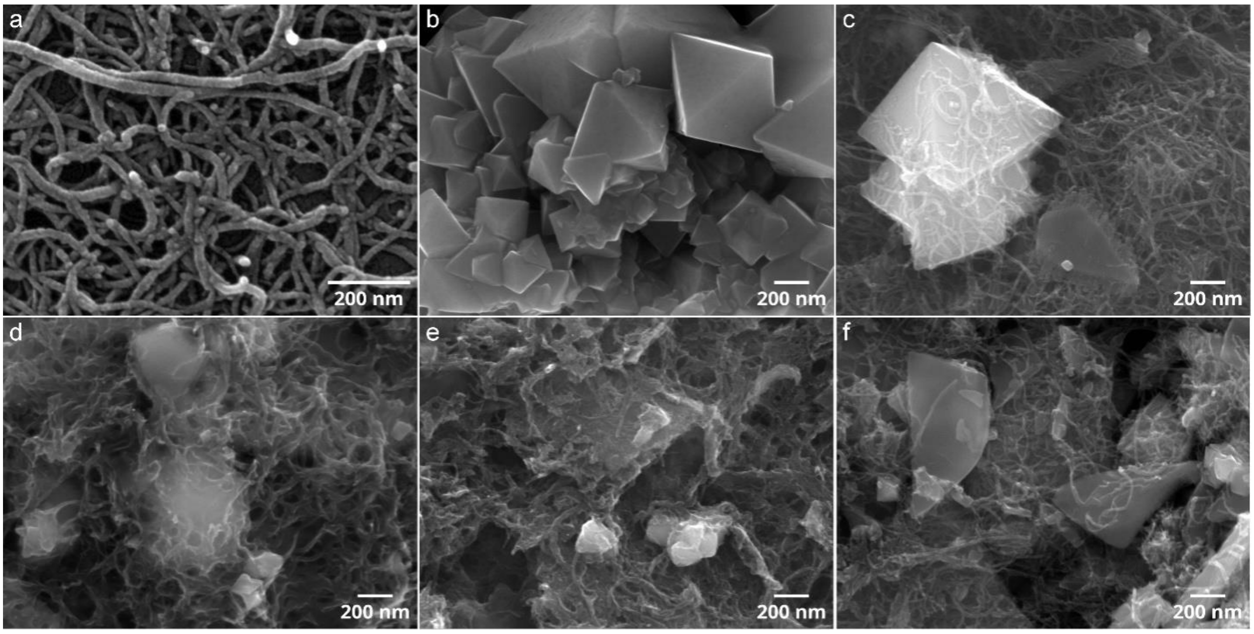

Figure S7. SEM images obtained for the following samples: (a) bare GNR(Cowt.%0), (b) bare Co_3O_4(Cowt.%75), (c) Co_3O_4(Cowt.%38)GNR, (d) Co_3O_4(Cowt.%55)GNR, (e) Co_3O_4GNR post-stability, and (f) Co_3O_4(Cowt.%53)GNR.

Figure S8 shows the SEM mapping images obtained for the bare Co_3O_4(Cowt.%75), as well as for the different Co_3O_4/GNR samples and the Co_3O_4GNR post-stability sample; the images show that the bare Co_3O_4(Cowt.%75) has mostly Co and O elements, with the O element found to be more visible on the catalyst surface (Figure S8a-e). For the Co_3O_4(Cowt.%38)GNR (Figure S8f-k), one can clearly observe the presence of the C element quite well distributed in the sample, with the Co element seen mostly in bigger Co_3O_4 structures, while the O element is seen mostly in the Co_3O_4 structures, including the small ones. The N element can be seen with less 'density', accompanying mostly the intensity of the O element. This mapping response shows that the N element is part of this catalyst, likewise the C element, which comes from the GNR. The Co_3O_4GNR post-stability sample (Figure S8l-q) follows a pattern of behaviour similar to that of the Co_3O_4(Cowt.%55)GNR sample (Figure 1d-i), with the N element present in low intensity.

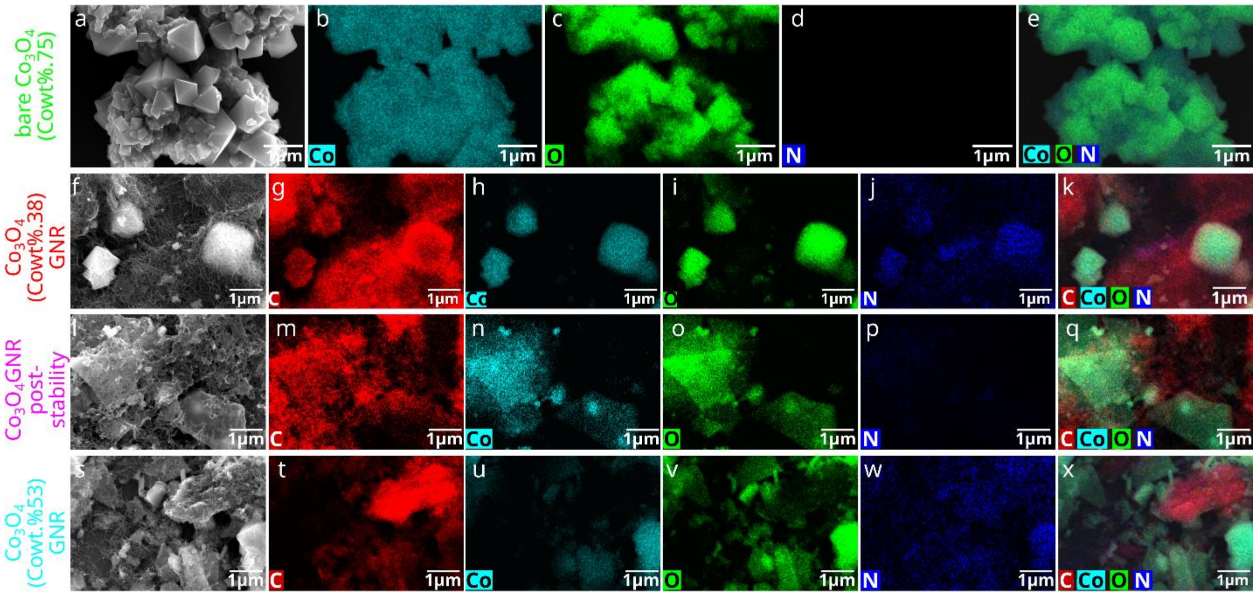

Figure S8. SEM mapping images for the bare Co_3O_4 sample, as well as for the different Co_3O_4/GNR samples and the Co_3O_4GNR post-stability sample investigated in the study.

Figure S9 shows the TEM and HR-TEM images, images used to produce the electron diffraction patterns, and the electron diffraction pattern images for the bare Co_3O_4(Cowt.%75) sample (only TEM images for the bare GNR(Cowt.%0) sample), as well as for the different Co_3O_4/GNR samples, and the Co_3O_4GNR post-stability sample investigated in this study.

Initially, the TEM images (Figures S9aa-ab) show the GNR. For the bare Co_3O_4(Cowt.%75) sample, one can see the pyramidal/plate structures (Figure S9u), clearly showing the presence of Co on the structures (Figure S9v) and the 'steps'. The finger patterns reveal the (222) and (400) Co_3O_4 exposed planes with distances of 0.23 and 0.20 nm (Figures S9w-x) (JCPDS 42-1467). The diffraction pattern (Figure S9z) of the exposed image from Figure S9y shows the ring difraction for the (111), (220), and (311) Co_3O_4 planes (JCPDS 42-1467).

The Co_3O_4 (Cowt.%38)GNR sample showed the presence of Co_3O_4 plate and GNR (Figure S9o), with Co confirmed on the Co_3O_4 plate (Figure S9p). The HR-TEM images (Figures S9q-r) exhibited the finger patterns of (222) Co_3O_4 exposed plane with a distance of 0.23 nm (JCPDS 42-1467), and (020) and (040) of the $(Co_3(Co(CN)_6)_2(H_2O)_{12})_{1.333}$ complex exposed planes with distances of 0.51 and 0.25 nm (JCPDS 96-152-5858), respectively. The diffraction pattern (Figure S9t) of the image displayed in Figure S9s showed the ring diffraction of (111), (020), (222), (131), (040), (042), and (242) planes of the $Co_3(Co(CN)_6)_2(H_2O)_{12})_{1.333}$ complex (JCPDS 96-

152-5858).

The Co_3O_4GNR post-stability sample showed a plate of Co_3O_4 which resembled a ‘benzene ring’ surrounded by a sufficient amount of GNR (Figure S9g), with the Co element (Co_3O_4) clearly covering one plate (Figure S9h). The HR-TEM images (Figures S9i-j) exhibited a kind of plate with finger patterns of (111) Co_3O_4 exposed plane with a distance of 0.46 nm (JCPDS 42-1467) and (042) of the $(Co_3(Co(CN)_6)_2(H_2O)_{12})_{1.333}$ complex exposed plane with a distance of 0.22 nm (JCPDS 96-152-5858). The diffraction pattern (Figure S9l) of the image displayed in Figure S9k showed the ring diffraction related to (111) Co_3O_4, as well as to (020), (022), (131), (040), and (133) planes of the $(Co_3(Co(CN)_6)_2(H_2O)_{12})_{1.333}$ complex (JCPDS 42-1467 and 96-152-5858).

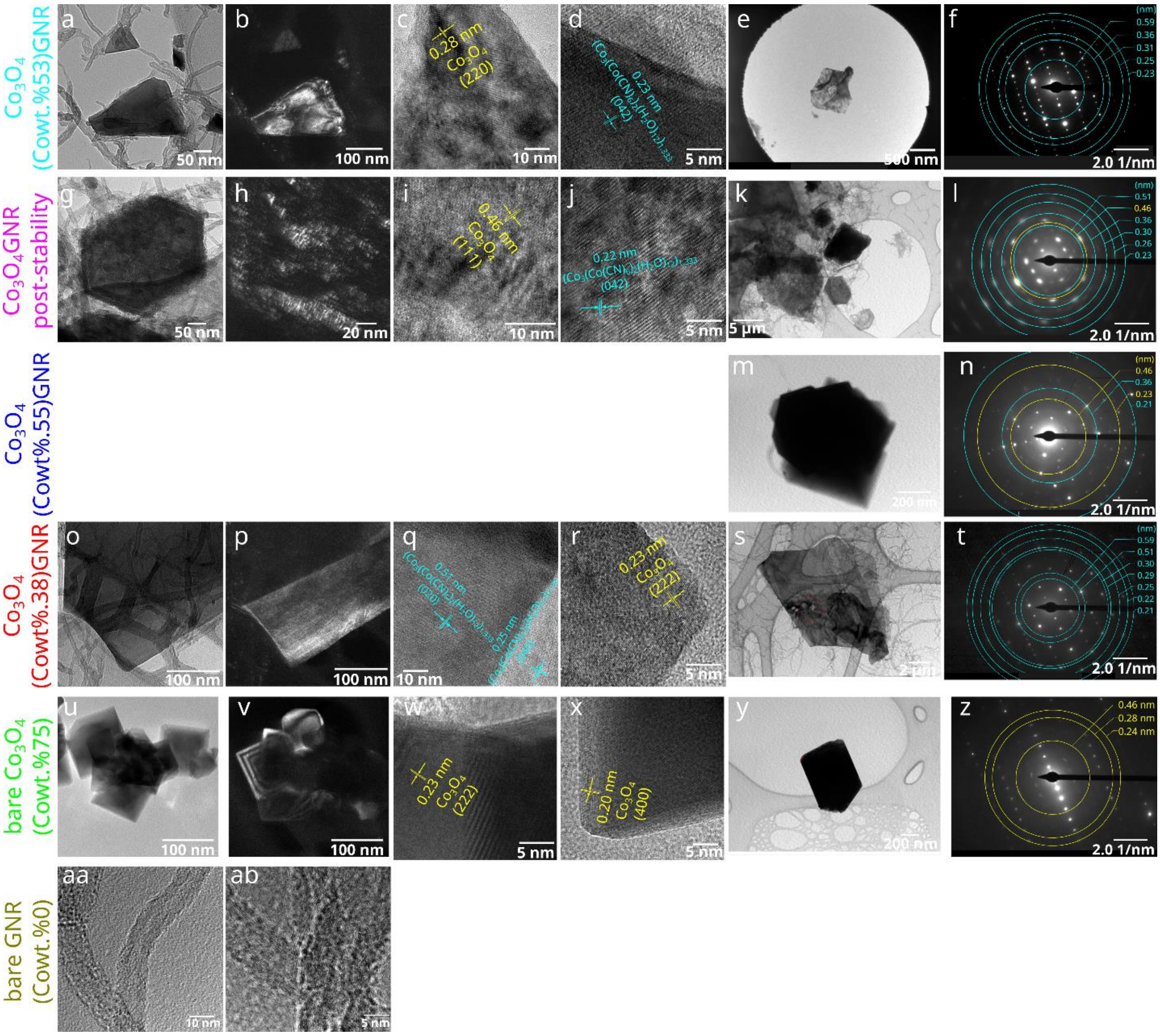

Figure S9. TEM and HR-TEM images, images used to produce the electron diffraction

patterns, and the electron diffraction pattern images for the bare Co_3O_4(Cowt.%75) sample (only TEM images for the bare GNR(Cowt.%0) sample), as well as for the different Co_3O_4/GNR samples, and the Co_3O_4GNR post-stability sample investigated in this study. It is important to note that, for the Co_3O_4(Cowt.%55)GNR sample, only the image used to produce the electron diffraction pattern and the electron diffraction pattern image are presented in this figure.

Cyclic Voltammetry Profile

As expected, the bare GNR(Cowt.%0) (Figure S10e) exhibited a discrete redox couple at around 1.0 V, which is typically characteristic of hydroquinone/quinone oxi-reduction with broad capacitive current densities[2]. Very few changes were observed in the cyclic voltammetry (CV) behaviour (current density changes) in the presence and absence of NO_3^- (Figure S10e). Interestingly, in the presence of NO_3^-, we noted an increase in current densities in potentials that were more negative than –0.40 V (Figure S10j).

The bare Co_3O_4(Cowt.%75) exhibited much lower current densities (capacitive current densities) compared to the bare GNR(Cowt.%0) catalyst and a well-defined current density peak at around 1.40 V, which is typically characteristic of Co^{2+}–Co^{3+} oxidation [5], with few changes in the presence of NO_3^- (Figure S10d). Remarkably, in the presence of NO_3^-, the current densities were found to increase in more negative potentials than 0.0 V; in the absence of NO_3^-, the current densities were found to increase in more negative potentials than –0.50 V (Figure S10i).

The Co_3O_4(Cowt.%38)GNR exhibited a mixed behavioural pattern observed for the bare GNR(Cowt.%0) and Co_3O_4(Cowt.%75) samples, with capacitive current densities slightly lower than those recorded for the bare GNR(Cowt.%0) sample, though higher than the densities recorded for the bare Co_3O_4(Cowt.%75) sample; the Co_3O_4(Cowt.%38)GNR sample displayed current density peak at around 1.34 V, which is typically characteristic of Co^{2+}–Co^{3+} oxidation [5], and very few changes in the presence of NO_3^- (Figure S10c). In the presence of NO_3^-, the current densities recorded considerable increases in more negative potentials than –0.1 V (Figure S10h). A similar behaviour (Figures S10a-b) was observed for the Co_3O_4(Cowt.%53)GNR and Co_3O_4(Cowt.%55)GNR when compared with Co_3O_4(Cowt.%38)GNR (Figure S10c), though the two former catalysts exhibited relatively lower capacitive current densities. The Co_3O_4(Cowt.%55)GNR and Co_3O_4(Cowt.%53)GNR samples (Figures S10f-g) recorded an increase in current densities to more negative potentials than 0.03 and –0.06 V, respectively, in the presence of NO_3^-.

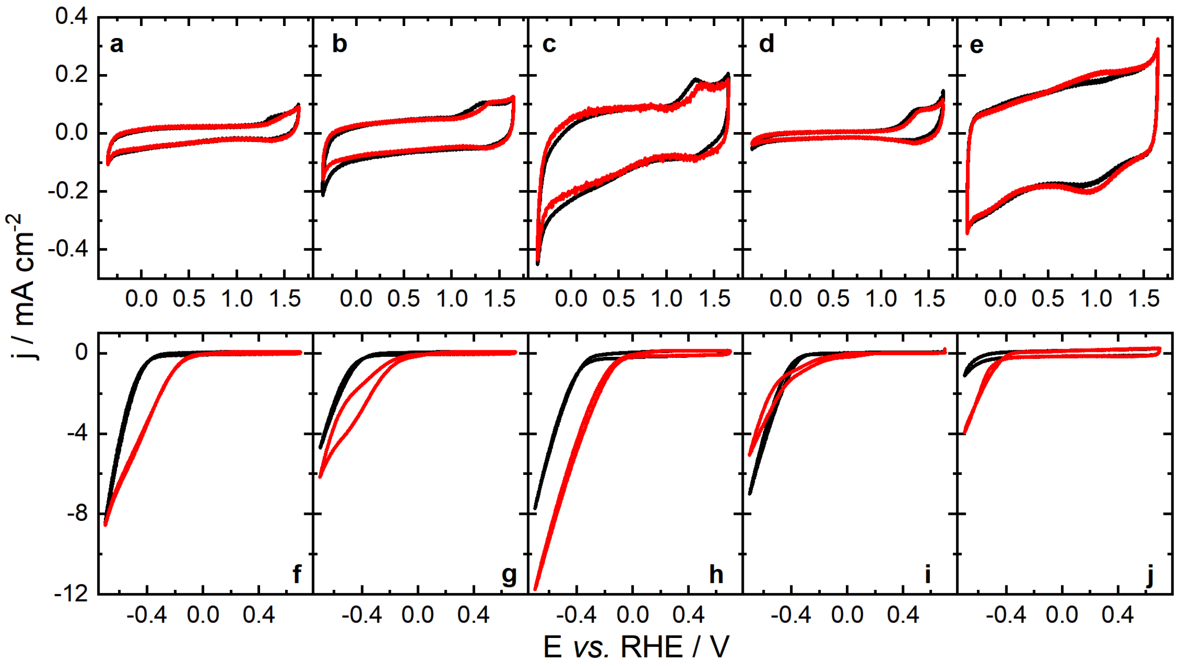

Figure S10. Cyclic voltammograms obtained for the CP electrodes modified with 37.5 μg cm^{-2} of the bare GNR(Cowt.%0) (e and j), bare Co_3O_4(Cowt.%75) (d and i), Co_3O_4(Cowt.%38)GNR (c and h), Co_3O_4(Cowt.%55)GNR (b and g), and Co_3O_4(Cowt.%53)GNR catalyst (a and f), employed in Ar–saturated 0.1 M K_2SO_4 (black lines), and in the presence of 40 mM $NaNO_3$ (red lines). Potential scan rate: 50 mV s^{-1} (scans were initiated at higher positive potentials).

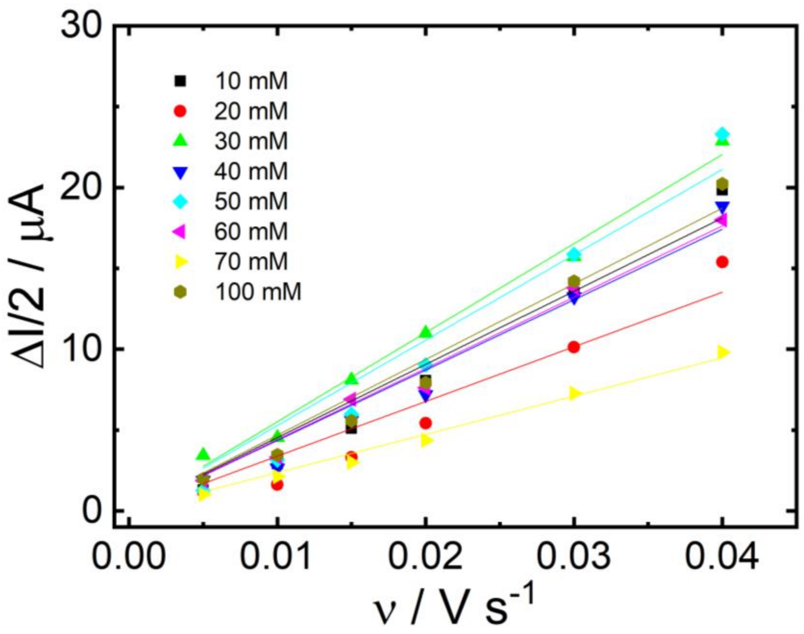

Figure S11. ΔI/2 *vs.* potential scan rates (ν) plots. The plots were constructed based on the differences between anodic (I_a) and cathodic (I_c) double layer charging currents (ΔI) from Figure S12.

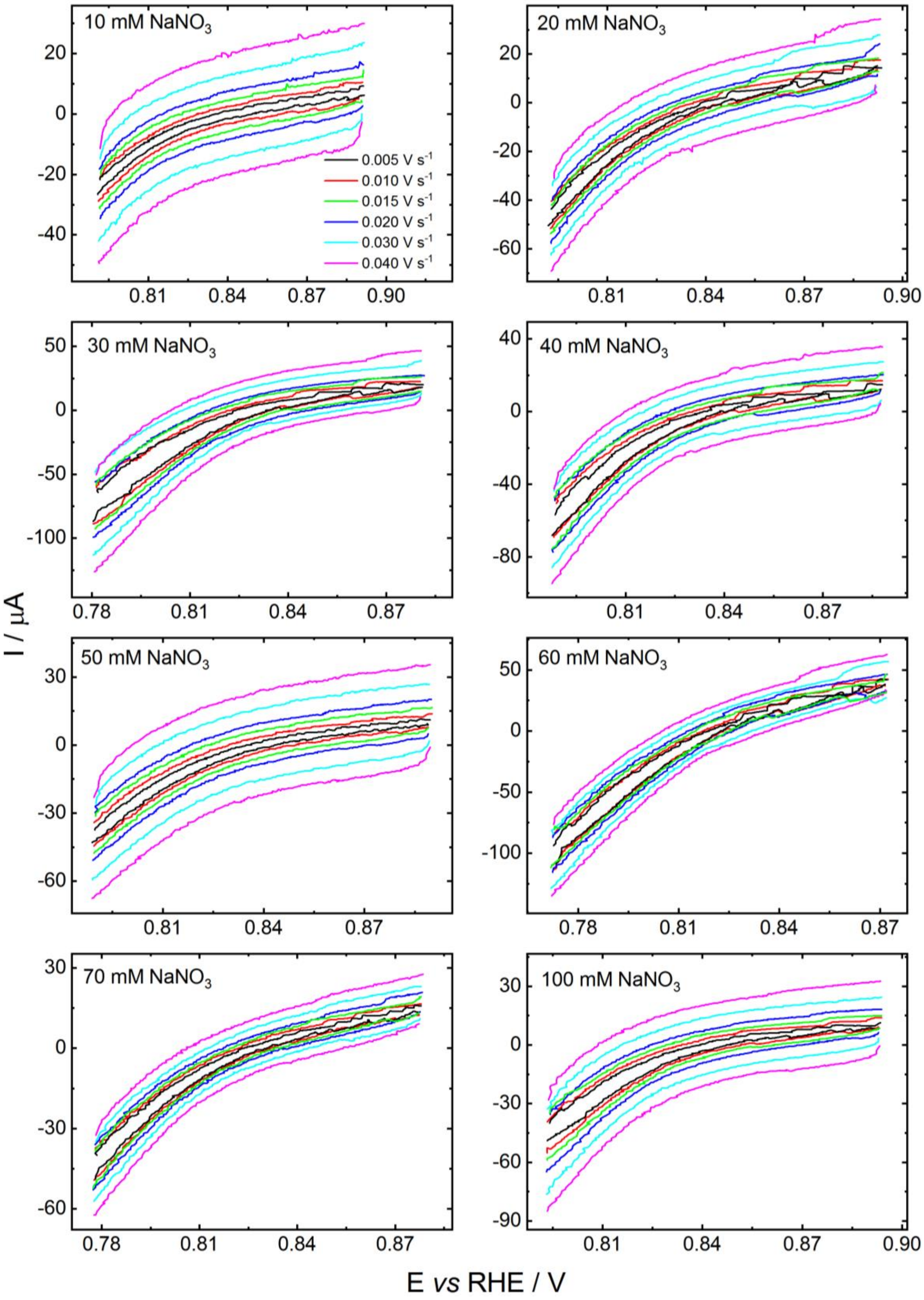

Figure S12. Cyclic voltammograms obtained in a non–Faradaic potential range for the CP electrode modified with 37.5 µg cm^{-2} of the Co_3O_4(Cowt.%55)GNR catalyst employed in Ar–saturated 0.1 M K_2SO_4 in the presence of varying $NaNO_3$ concentrations. Prior to the beginning of the subsequent potential sweep, the modified electrode was kept at each vertex potential for 10 seconds. Scanning was initiated at higher potentials.

Table S5. C_{dl} values (eq. 1), obtained from the data in Figure S11, and ECSA values recorded for the CP electrode modified with 37.5 µg cm^{-2} of the Co_3O_4(Cowt.%55)GNR catalyst, employed in Ar–saturated 0.1 M K_2SO_4 in the presence of varying $NaNO_3$ concentrations.

NO_3^- concentration / mM	C_{dl}/ mF	ECSA / cm²
**10**	0.45	11.3
**20**	0.34	8.5
**30**	0.55	13.8
**40**	0.43	10.9
**50**	0.53	13.2
**60**	0.44	11.0
**70**	0.24	5.9
**100**	0.47	11.7

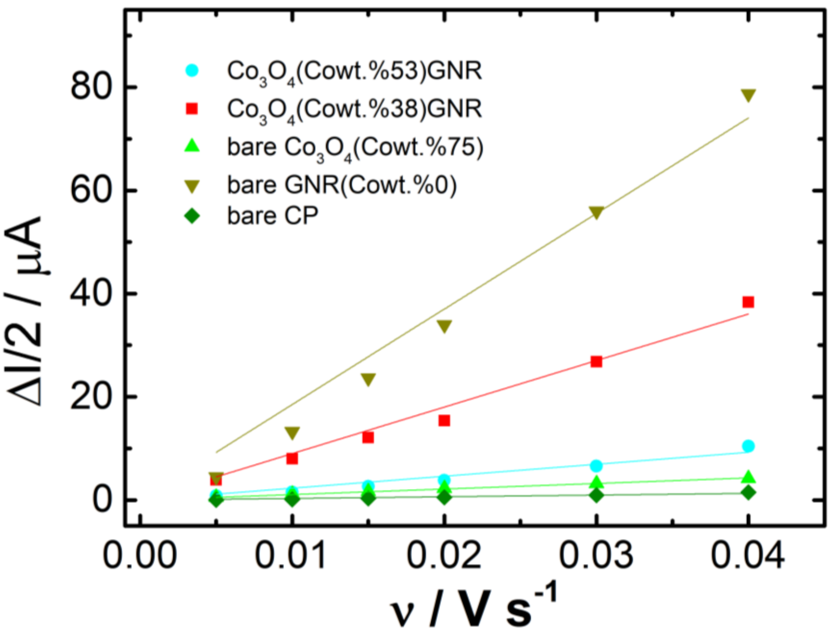

Figure S13. $\Delta I/2$ *vs.* potential scan rates (ν) plots. The plots were constructed based on the differences between the anodic (I_a) and cathodic (I_c) double layer charging currents (ΔI) from Figure S14.

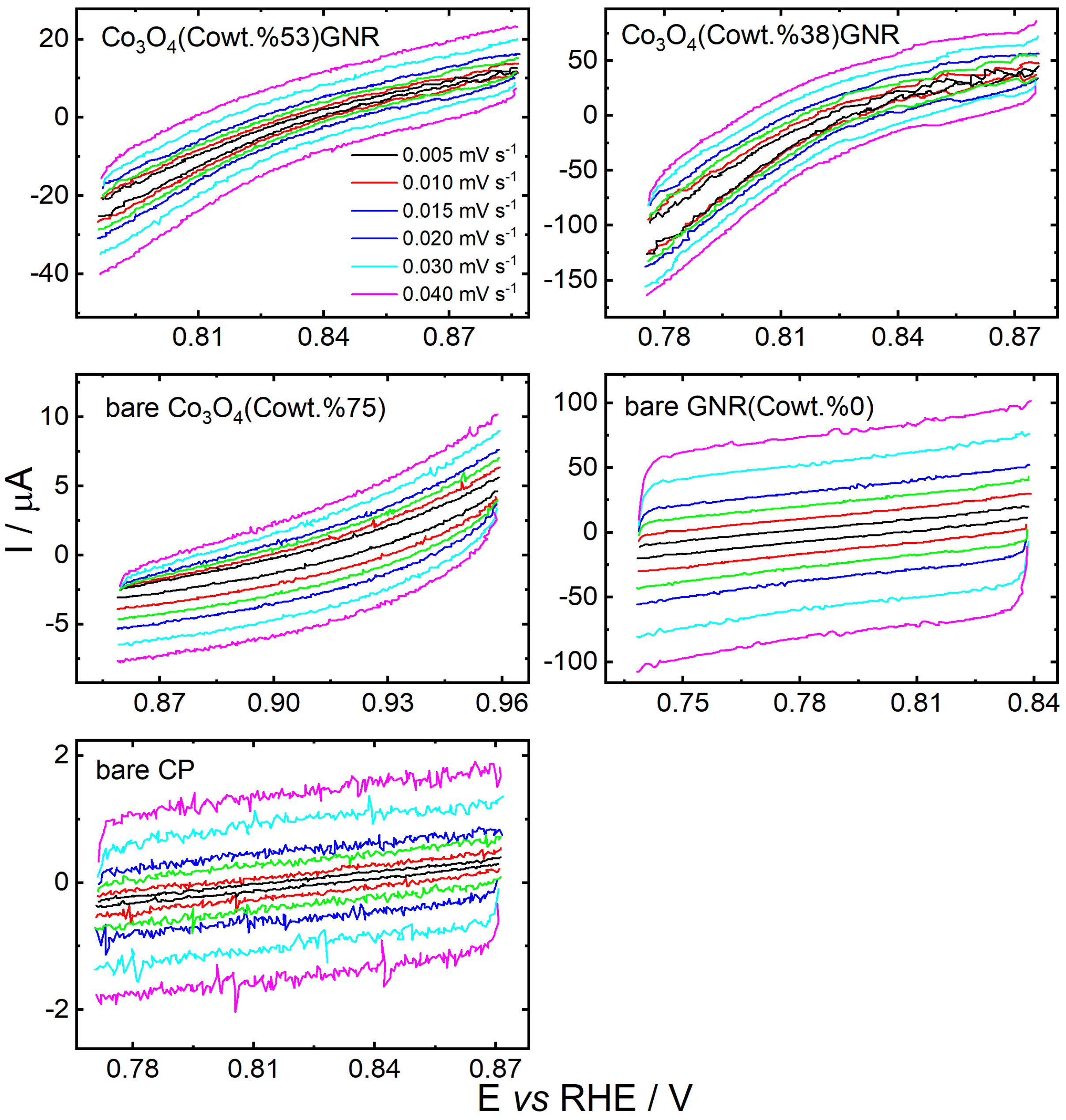

Figure S14. Cyclic voltammograms obtained in a non–Faradaic potential range for the different electrodes investigated, employed in Ar–saturated 0.1 M K_2SO_4, in the presence of 40 mM $NaNO_3$. Prior to the beginning of the subsequent potential sweep, the modified electrode was kept at each vertex potential for 10 seconds. Scanning was initiated at higher potentials.

Table S6. C_{dl} values (eq. 1), obtained from the data in Figure S13, and ECSA values recorded for the different electrodes investigated (for the modified electrodes, an amount of 37.5 µg cm^{-2} of the catalyst was applied), employed in Ar–saturated 0.1 M K_2SO_4 in the presence of 40 mM $NaNO_3$.

Catalyst	C_{dl} / mF	ECSA / cm²
**Co_3O_4(Cowt.%53)GNR**	0.23	5.8
**Co_3O_4(Cowt.%38)GNR**	0.90	22.5
**bare Co_3O_4(Cowt.%75)**	0.11	2.7
**bare GNR(Cowt.%0)**	1.85	46.3
**bare CP**	0.03	0.8

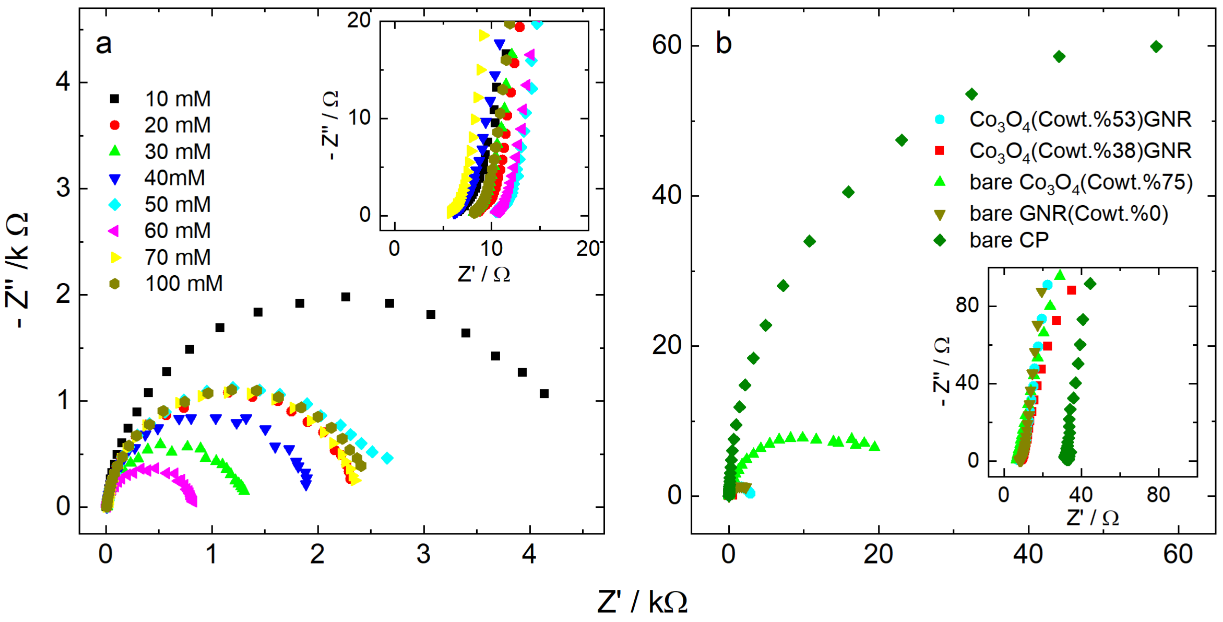

Figure S15. EIS results (Nyquist plots) obtained for the (a) CP electrode modified with 37.5 µg cm^{-2} of the Co_3O_4(Cowt.%55)GNR catalyst, employed in Ar–saturated 0.1 M K_2SO_4 in the presence of varying $NaNO_3$ concentrations, and (b) different electrodes employed in Ar–saturated 0.1 M K_2SO_4 in the presence of 40 mM $NaNO_3$, where the open circuit potential (OCP) value was employed as a constant potential for the EIS analysis.

Table S7. Resistance of electrolytic solution (R_S) and charge transfer (R_{ct}) values obtained for the CP electrode modified with 37.5 µg cm^{-2} of the Co_3O_4(Cowt.%55)GNR catalyst employed in Ar–saturated 0.1 M K_2SO_4 in the presence of varying $NaNO_3$ concentrations, and R_S and R_{ct} values obtained for the electrodes modified with 37.5 µg cm^{-2} of the catalyst employed in Ar–saturated 0.1 M K_2SO_4 in the presence of 40 mM $NaNO_3$. The R_S and R_{ct} values were obtained based on the EIS results (data shown in Figure S15). The solution pH (0.1 M K_2SO_4 with varying $NaNO_3$ concentrations or 40 mM $NaNO_3$) are reported below before and after the chronoamperometry experiments.

NO_3^- concentration / mM	R_S / Ω	R_{ct} / kΩ	Solution pH before chronoamperometry	Solution pH after chronoamperometry
**10**	7.3	4.5	9.08	9.45
**20**	9.6	2.3	9.41	12.39
**30**	9.0	1.4	9.27	11.39
**40**	6.6	1.9	9.77	10.22
**50**	11.6	2.7	9.64	9.72
**60**	11.4	0.8	9.12	12.1
**70**	6.5	2.4	9.11	10.23
**100**	9.0	2.5	9.28	12.35

Catalyst	R_S / Ω	R_{ct} / kΩ	Solution pH before chronoamperometry	Solution pH after chronoamperometry
**bare CP**	34.5	>>60.0	8.58	9.85
**bare GNR(Cowt.%0)**	8. 9	2.6	9.37	11.56
**bare Co_3O_4(Cowt.%75)**	7.3	16.0	10.7	12.09
**Co_3O_4(Cowt.%38)GNR**	9.3	0.7	10.18	12.51
**Co_3O_4(Cowt.%53)GNR**	8.7	2.7	9.76	11.71

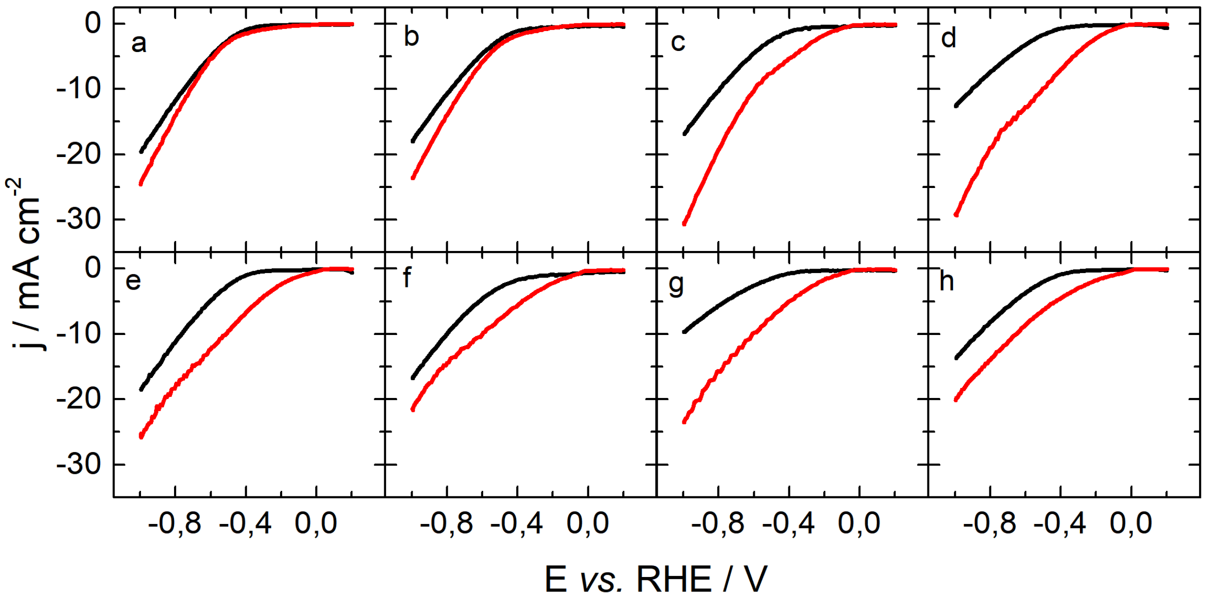

Figure S16. Stationary linear sweep voltammograms (LSVs) recorded for the CP electrode modified with 37.5 µg cm^{-2} of the Co_3O_4(Cowt.%55)GNR catalyst employed in Ar–saturated 0.1 M K_2SO_4 (black lines), in the presence of varying $NaNO_3$ concentrations (a = 10, b = 20, c = 30, d = 40, e = 50, f = 60, g = 70, and h = 100 mM) (red lines), at scan rate (ν) = 5 mV s^{-1}. Scans were initiated at 0.2 V.

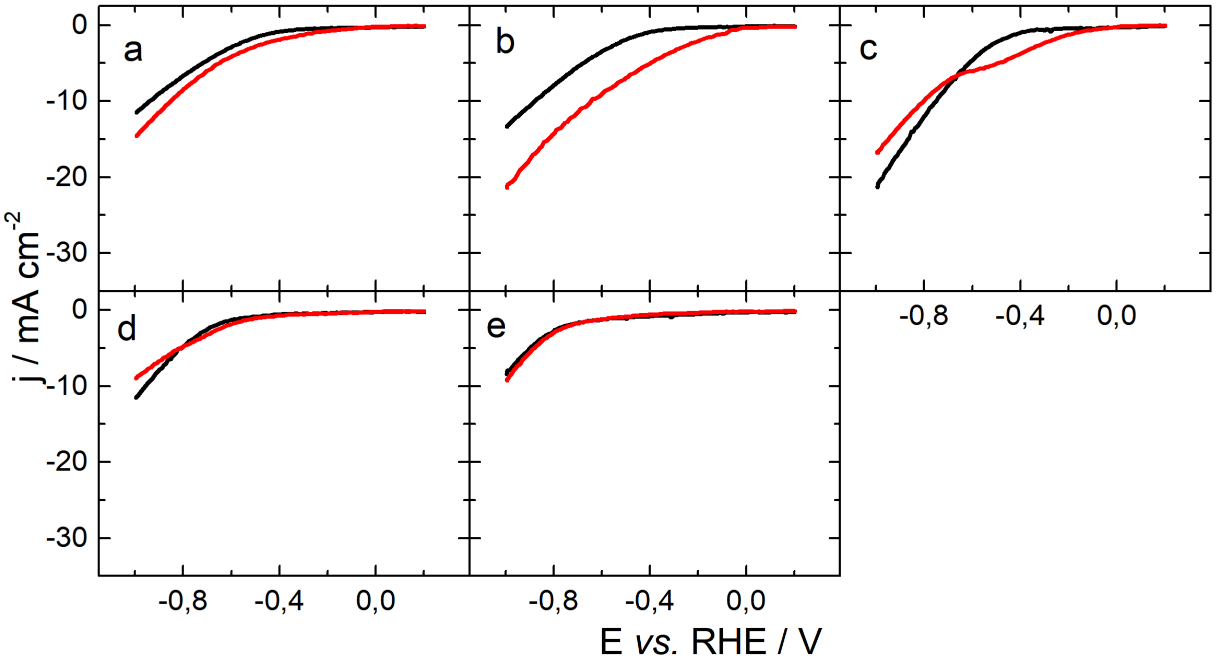

Figure S17. Stationary linear sweep voltammograms (LSVs) recorded for the bare CP (e) and CP electrode modified with 37.5 µg cm^{-2} of the bare GNR(Cowt.%0) (d), bare Co_3O_4(Cowt.%75)(c), Co_3O_4(Cowt.%38)GNR (b), and Co_3O_4(Cowt.%53)GNR (a) catalysts employed in Ar–saturated 0.1 M K_2SO_4 (black lines) in the presence of 40 mM $NaNO_3$ (red lines), at ν = 5 mV s^{-1}. Scans were initiated at 0.2 V.

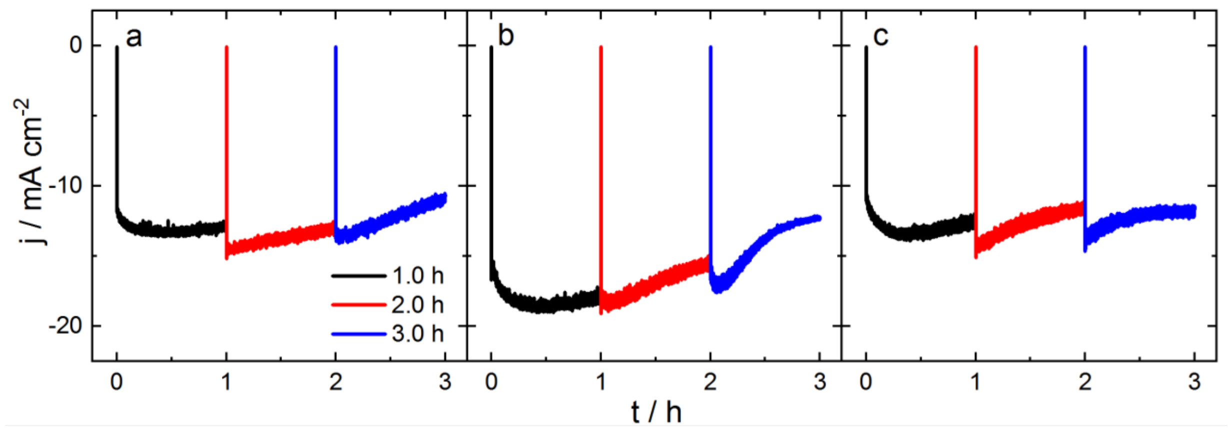

Figure S18. Chronoamperometric results ((a) –0.5, (b) –0.6, and (c) –0.7 V) obtained for the Co_3O_4(Cowt.%55)GNR catalyst employed in Ar–saturated 0.1 M K_2SO_4 in the presence of 40 mM $NaNO_3$. After each one hour of chronoamperometric experiment, the experiment was interrupted in order to remove aliquots from the solution.

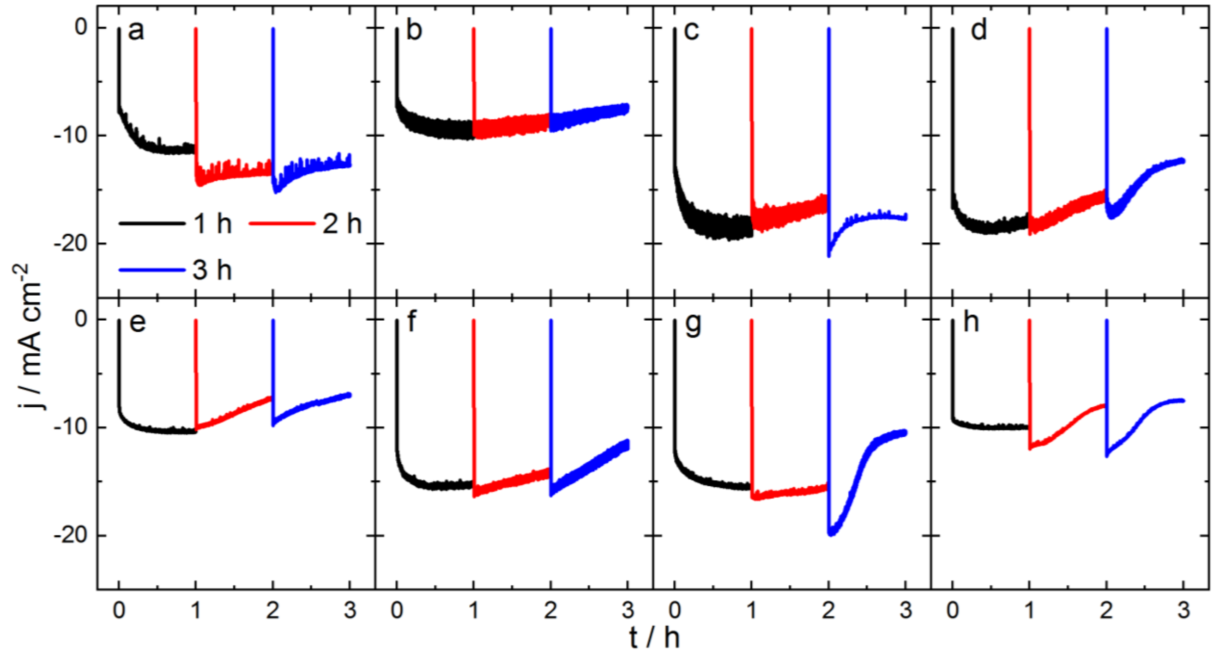

Figure S19. Chronoamperometric results (–0.6 V) obtained for the Co_3O_4(Cowt.%55)GNR catalyst employed in Ar–saturated 0.1 M K_2SO_4 in the presence of (a) 10, (b) 20, (c) 30, (d) 40, (e) 50, (f) 60, (g) 70, and (h) 100 mM $NaNO_3$ concentration. After each one hour of chronoamperometric experiment, the experiment was interrupted in order to remove aliquots from the solution.

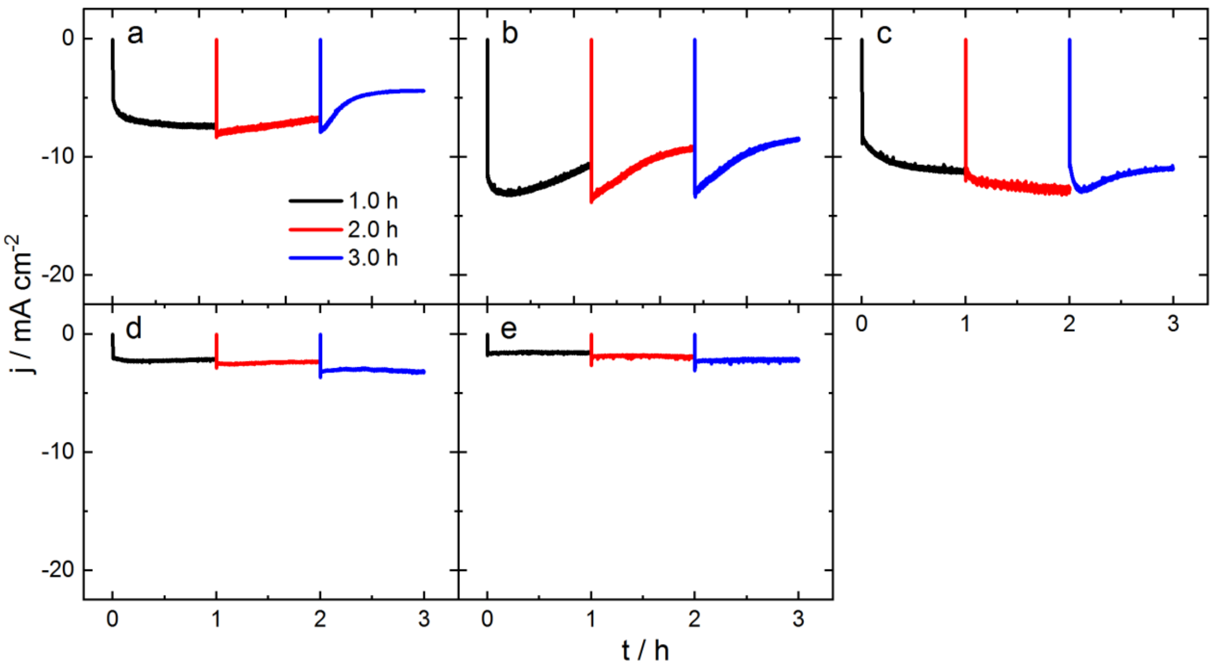

Figure S20. Chronoamperometric results obtained for the (a) Co_3O_4(Cowt.%53)GNR, (b) Co_3O_4(Cowt.%38)GNR, (c) bare Co_3O_4(Cowt.%75), (d) bare GNR(Cowt.%0), and (e) bare CP catalysts employed in Ar–saturated 0.1 M K_2SO_4, in the presence of 40 mM $NaNO_3$. After each one hour of chronoamperometric experiment, the experiment was interrupted in order to remove aliquots from the solution.

The nitrate concentration was determined by the colorimetric method in the wavelength range of 300-190 nm, using UV spectroscopy[6]. For this analysis, we mixed the following in a volumetric flask: 100 µL HCl solution (1.0 M), 10 µL of 0.8 wt% sulfamic acid aqueous solution, few mL of the standard or sample solution, and water up to 10 mL; the mixture was then left to rest for 10 min. It is worth noting that the samples and standards were collected and diluted, taking the detection range into account. The nitrate concentrations were analyzed relative to the absorbance at 220 nm minus 2 times (2*) the absorbance at 275 nm. The nitrate concentrations were determined based on the application of $NaNO_3$ standard solutions with concentration ranging from 0.5 to 6.4 ppm in 0.1 M K_2SO_4 solution (Figure S21).

The Griess reagent was used for the determination of nitrite concentration through UV-visible spectroscopy [7]; the reagent was prepared by solubilizing 0.4 g of p-aminobenzenesulfonamide and 0.02 g of N-(1-Naphthyl)-ethylenediamine dihydrochloride in 1 mL of H_3PO_4, followed by the addition of water up to the volume of 5 mL, in a volumetric flask. In a typical colorimetric assay, 0.1 mL of the Griess reagent was poured into 0.4 mL of the standard or sample solution and water was added into the mixture up to a volume of 10 mL in a volumetric flask; the mixture was then left to rest in the dark for 20 minutes. After that, the absorption spectrum was measured. The

NO_2^- concentration was calculated using the standard NO_2^- solutions with concentrations ranging from 0.01 to 1 ppm in 0.1 M K_2SO_4 solution. The absorption intensity at wavelength of 540 ηm was evaluated in relation to NO_2^- concentration (Figure S21).

The ammonium concentration was also determined by UV-visible spectroscopy using the indophenol blue method[8]. Solutions A and B were prepared separately in two volumetric flasks: Solution A was prepared using 1 g of phenol and 5 mg of nitroprusside dissolved in water (filled up to 100 mL); Solution B was prepared using 0.5 g of NaOH and 0.84 mL of sodium hypochlorite solution (10-12%) solubilized with water (filled up to 100 mL). For the preparation of the samples and standards to be measured, a flask was employed; the solutions were prepared by adding an aliquot of 10 µL of the sample or standard solution and 4.99 mL of each solution A and B in a flask. The solutions were then left to rest for 1 h; after that, the UV-visible absorption spectra were measured, where the ammonium concentration was evaluated relative to absorbance at 625 nm. The absorbance-ammonia concentration curve was obtained using a standard NH_4Cl solution with concentrations ranging from 0.02 to 0.7 ppm in 0.1 M K_2SO_4 solution (Figure S21).

The hydrazine concentration was determined using the method developed by Watt and Chrisp [9]. For this analysis, a solution was prepared by dissolving 0.2 g of p-(dimethylamine)benzaldehyde in 1 mL HCl (concentrated), followed by the addition of 10 mL of ethanol – employed as a colour reagent solution. The absorption spectra were measured in solutions prepared by mixing 0.4 mL of Watt and Chrisp's reagent, 0.3 mL of the sample or standard solution and 9.3 mL water (10 mL volumetric flask), followed by fast stirring and leaving the mixture to rest for 10 min at room temperature. The absorbance of each of the resulting solution was measured at 455 nm (Figure S21).

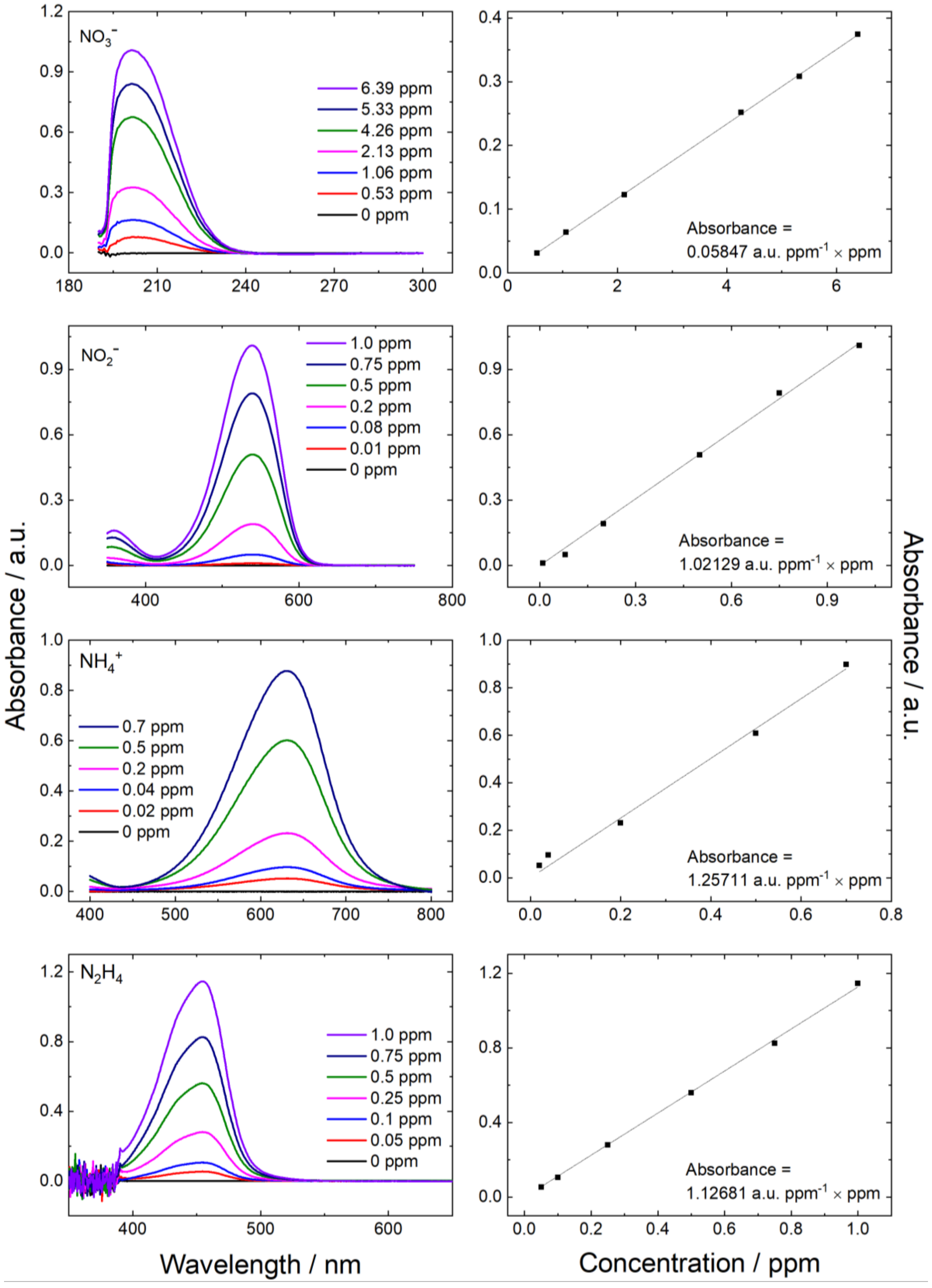

Figure S21. UV-visible standard curves (images on the left) used to quantify NO_3^-, NO_2^-, NH_4^+, and N_2H_4 products. The images on the right show the linear behaviour based on the UV-visible responses for each compound with its respective linear equation.

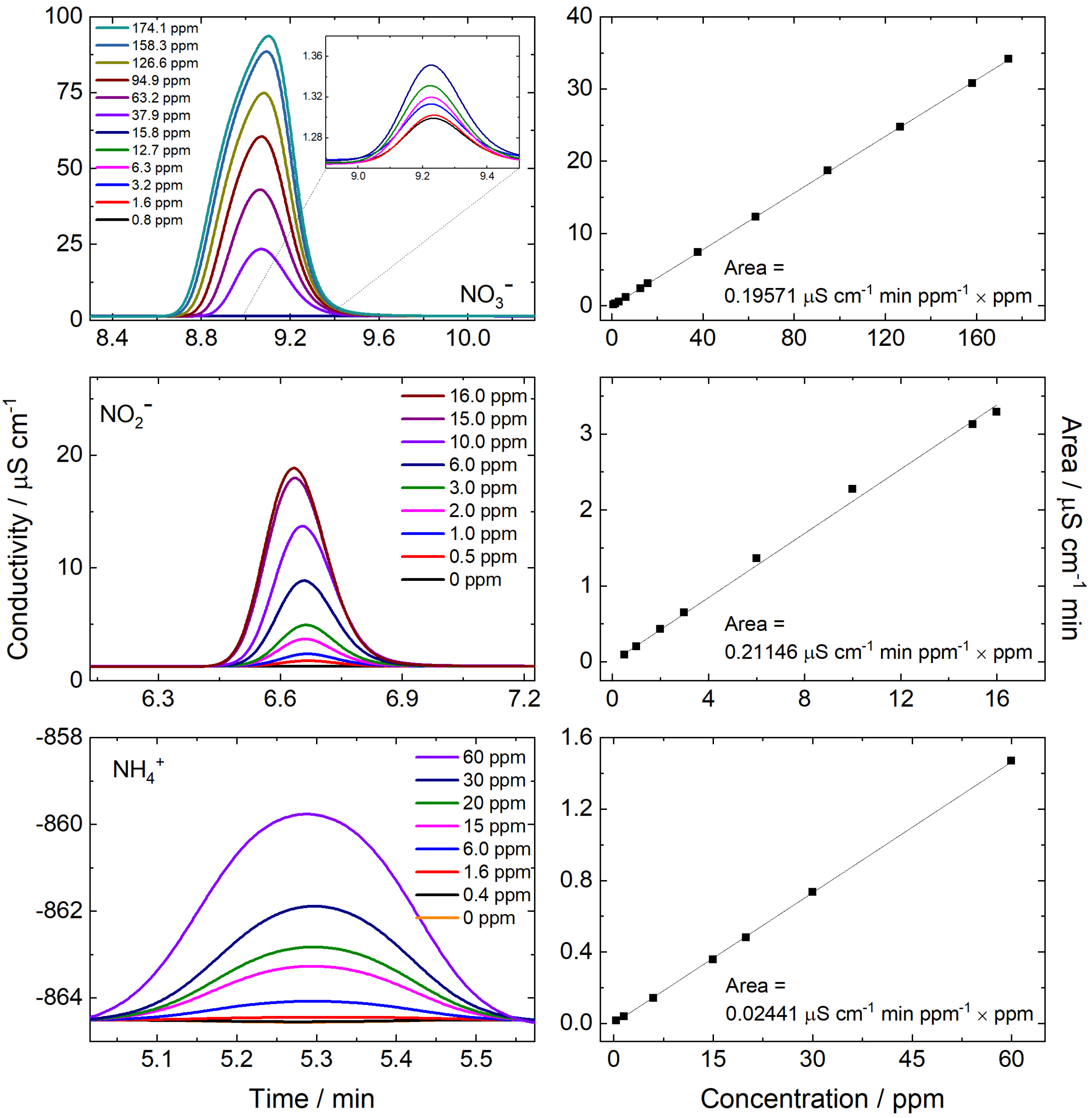

Figure S22. Ion chromatography (IC) standard curves (images on the left) used to quantify NO_3^-, NO_2^-, and NH_4^+ products. The images on the right show the linear behaviour based on the IC responses for each compound with its respective linear equation.

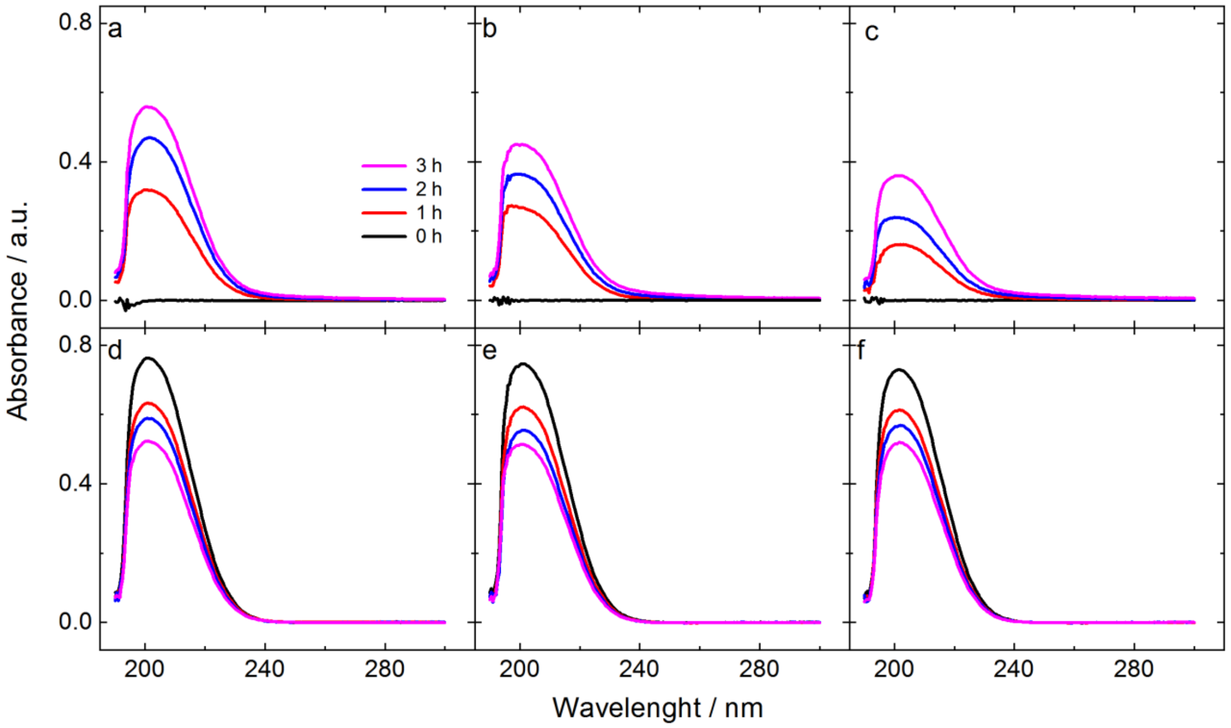

Figure S23. UV curves used to quantify NO_3^- from the chronoamperometric experiments conducted based on the application of the Co_3O_4(Cowt.%55)GNR catalyst in Ar–saturated 0.1 M K_2SO_4, in the presence of 40 mM $NaNO_3$, at the following potentials: (a) –0.5, (b) –0.6, and (c) –0.7 V (cell anodic branch, aliquot of 60 µL) and (d) –0.5, (e) –0.6, and (f) –0.7 V (cell cathodic branch, aliquot of 20 µL).

Table S8. NO_3^- migration percentage relative to time obtained from the UV results for the H-cell anodic branch. The experiment was conducted using Ar–saturated 0.1 M K_2SO_4 in the presence of 40 mM $NaNO_3$ (as electrolyte solution), without the application of any potential.

**Time / h**	**Anodic branch / %**
0	0
1	0.09
2	2.96
3	3.86

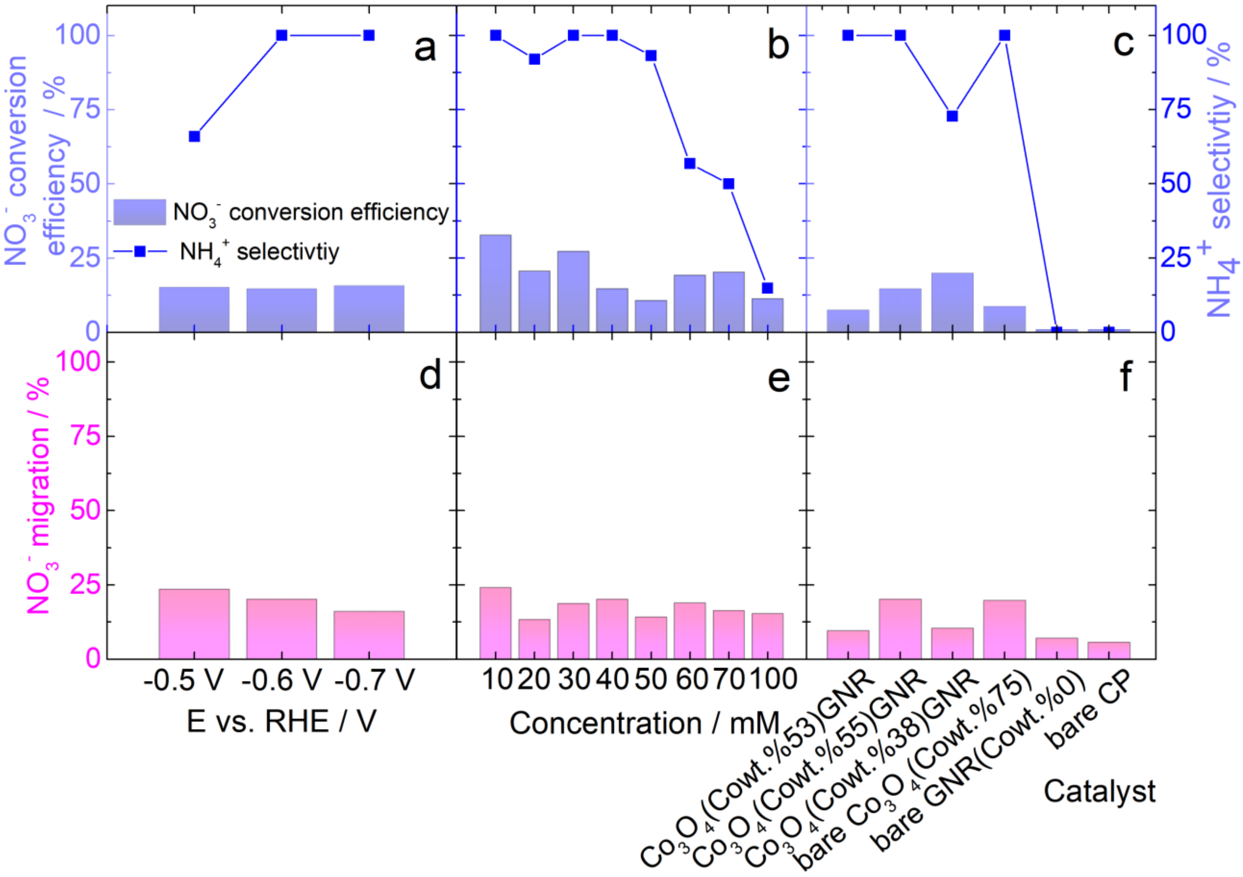

Figure S24. Results obtained after three hours of chronoamperometric experiments based on the application of the following: Co_3O_4(Cowt.%55)GNR catalyst employed in Ar–saturated 0.1 M K_2SO_4 in the presence of 40 mM $NaNO_3$, at applied potentials of –0.5, –0.6, and –0.7 V ((a) NO_3^- conversion efficiency and NH_4^+ selectivity and (d) NO_3^- migration); Co_3O_4(Cowt.%55)GNR catalyst employed in Ar–saturated 0.1 M K_2SO_4 in the presence of 10, 20, 30, 40, 50, 60, 70, and 100 mM $NaNO_3$ at –0.6 V ((b) NO_3^- conversion efficiency and NH_4^+ selectivity and (e) NO_3^- migration); and Co_3O_4(Cowt.%53)GNR, Co_3O_4(Cowt.%55)GNR, Co_3O_4(Cowt.%38)GNR, bare Co_3O_4(Cowt.%75), bare GNR(Cowt.%0), and bare CP catalysts employed in Ar–saturated 0.1 M K_2SO_4 in the presence of 40 mM $NaNO_3$ at –0.6 V ((c) NO_3^- conversion efficiency and NH_4^+ selectivity and (f) NO_3^- migration).

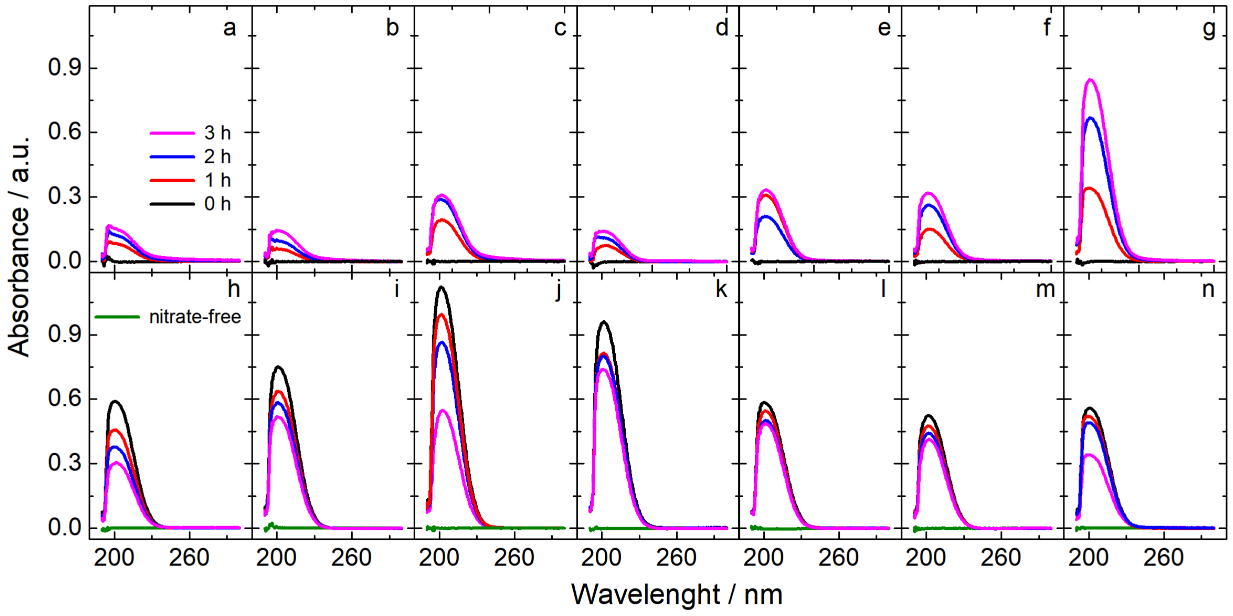

Figure S25. UV curves used to quantify NO_3^- from the chronoamperometric experiments conducted based on the application of the Co_3O_4(Cowt.%55)GNR catalyst in Ar–saturated 0.1 M K_2SO_4 in the presence of (a) 10, (b) 20, (c) 30, (d) 50, (e) 60, (f) 70, and (g) 100 mM $NaNO_3$ concentration (cell anodic branch, aliquot of 60, 60, 60,20,30, 30, and 20µL, respectively) and (h) 10, (i) 20, (j) 30, (k) 50, (l) 60, (m) 70, and (n) 100 mM $NaNO_3$ concentration (cell cathodic branch, aliquot of 20,40, 40,20, 10, 0.8,and 0.6µL, respectively); applied potential: –0.6 V.

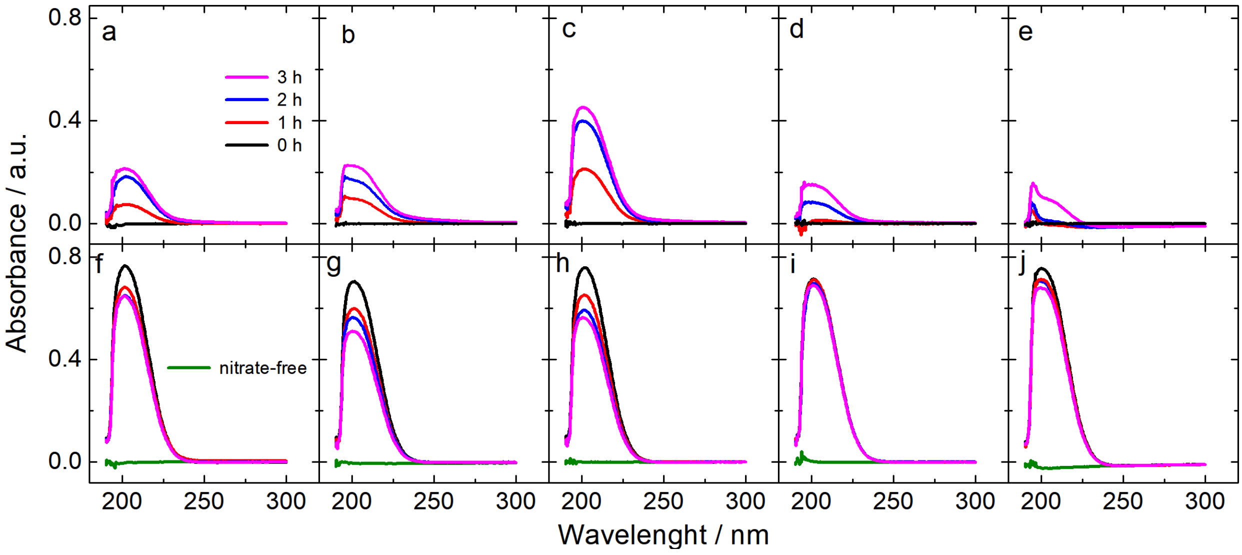

Figure S26. UV curves used to quantify NO_3^- from the chronoamperometric experiments conducted based on the application of the (a) Co_3O_4(Cowt.%53)GNR, (b) Co_3O_4(Cowt.%38)GNR, (c) bare Co_3O_4(Cowt.%75), (d) bare GNR(Cowt.%0), and (e) bare CP (cell anodic branch, aliquot of 60 µL), and (f) Co_3O_4(Cowt.%53)GNR, (g) Co_3O_4(Cowt.%38)GNR, (h) bare Co_3O_4(Cowt.%75), (i) bare GNR(Cowt.%0), and (j) bare CP (cell cathodic branch, aliquot of 20 µL) catalysts in Ar–saturated 0.1 M K_2SO_4,

in the presence of 40 mM $NaNO_3$; applied potential: –0.6 V.

Figure S24c shows that at the potential of –0.6 V vs RHE, the Co_3O_4(Cowt.%38)GNR catalyst recorded the highest NO_3^- conversion efficiency (20.0%) among the catalysts investigated, though it presented lower NH_4^+ selectivity (72.77%) in comparison with the Co_3O_4(Cowt.%55)GNR catalyst (with NH_4^+ selectivity of 100%). The bare Co_3O_4(Cowt.%75) catalyst exhibited NH_4^+ selectivity of 100% despite its extremely low NO_3^- conversion efficiency (8.83%). The bare GNR(Cowt.%0) and CP catalysts exhibited negligible NH_4^+ selectivity with some NO_3^- conversion efficiency. NO_3^- migration was high (around 20%, Figure S24f) for the Co_3O_4(Cowt.%55)GNR and bare Co_3O_4(Cowt.%75) catalysts, low for the Co_3O_4(Cowt.%38)GNR catalysts, and much lower for the bare GNR(Cowt.%0) and CP catalysts.

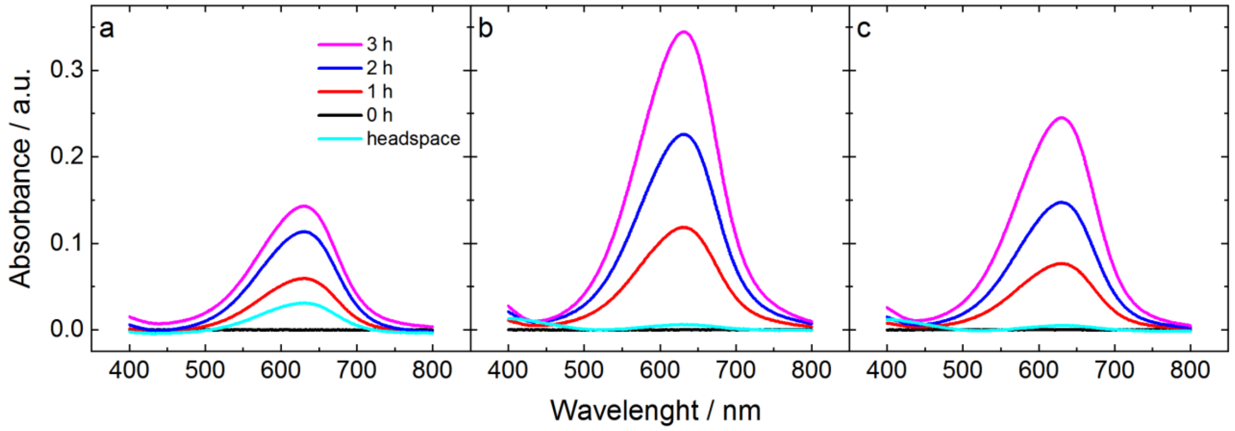

Figure S27. UV-visible curves used to quantify NH_4^+ from the chronoamperometric experiments conducted based on the application of the Co_3O_4(Cowt.%55)GNR catalyst in Ar–saturated 0.1 M K_2SO_4 in the presence of 40 mM $NaNO_3$, at the potentials of (a) –0.5, (b) –0.6, and (c) –0.7 V (cell cathodic branch, aliquot of 20 µL).

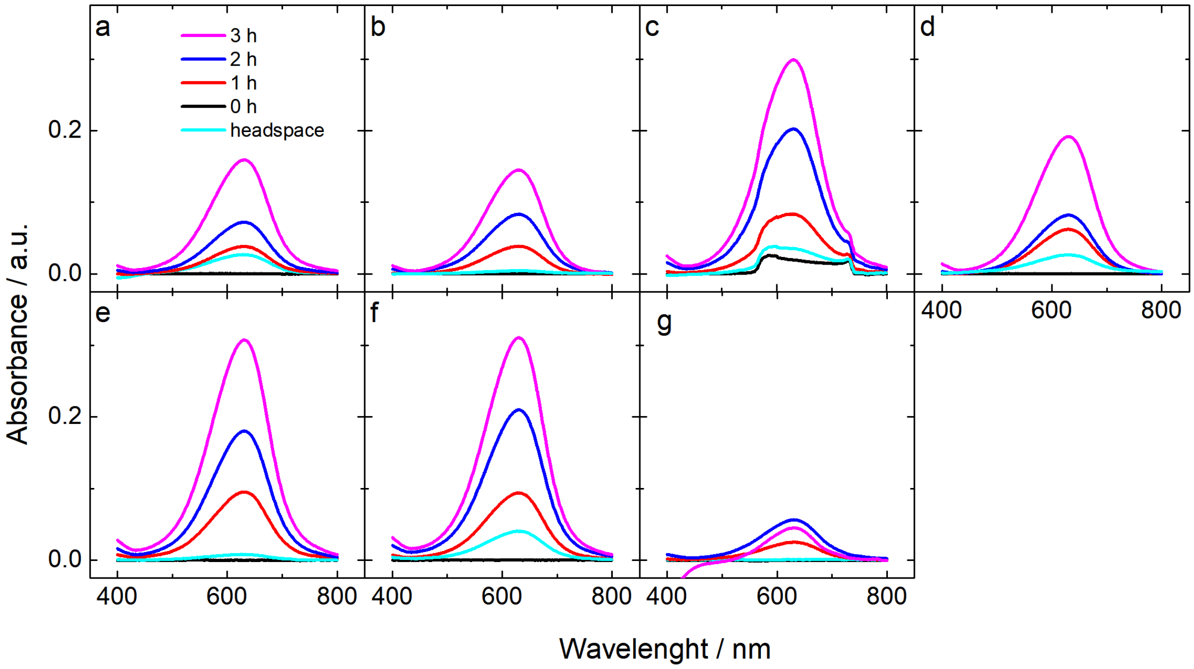

Figure S28. UV-visible curves used to quantify NH_4^+ from the chronoamperometric experiments conducted based on the application of the Co_3O_4(Cowt.%55)GNR catalyst in Ar–saturated 0.1 M K_2SO_4 in the presence of 40 mM $NaNO_3$, using the following $NaNO_3$ concentrations: (a) 10, (b) 20, (c) 30, (d) 50, (e) 60, (f) 70, and (g) 100 mM $NaNO_3$ (cell cathodic branch, aliquot of 20 µL); applied potential:–0.6 V.

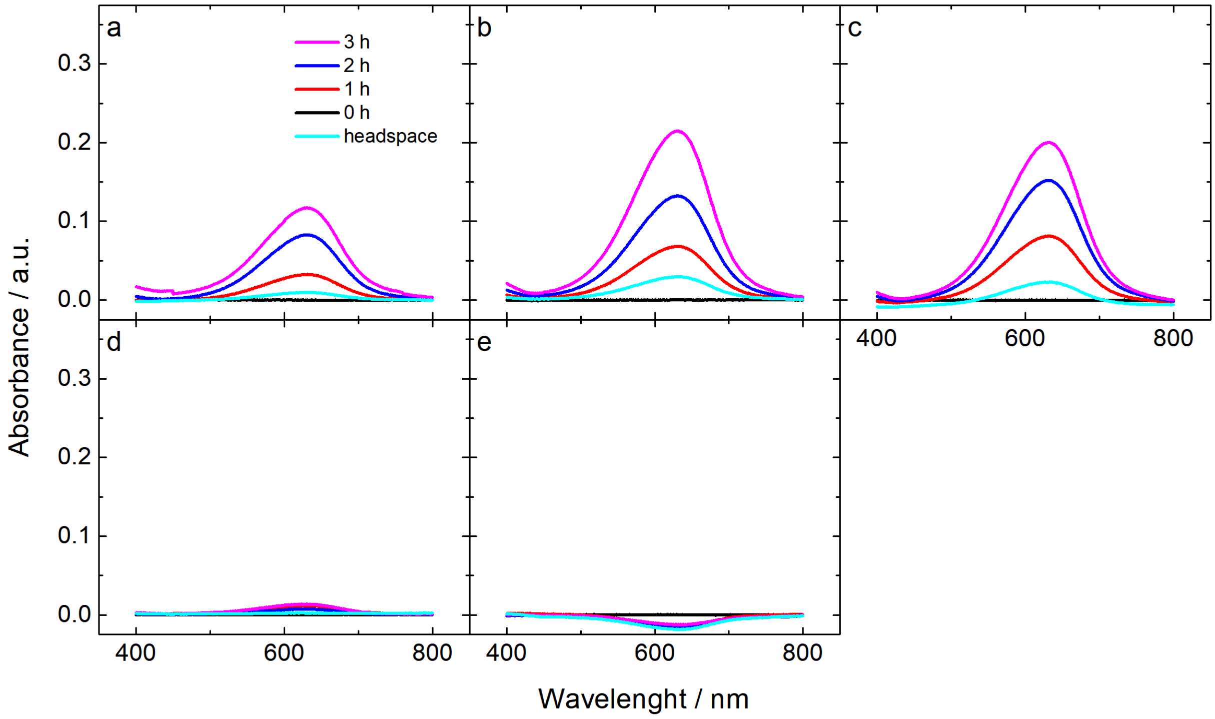

Figure S29. UV-visible curves used to quantify NH_4^+ from the chronoamperometric experiments conducted based on the application of the (a) Co_3O_4(Cowt.%53)GNR, (b) Co_3O_4(Cowt.%38)GNR, (c) bare Co_3O_4(Cowt.%75), (d) bare GNR(Cowt.%0), and (e)

bare CP (cell cathodic branch, aliquot of 20 µL) catalysts in Ar–saturated 0.1 M K_2SO_4, in the presence of 40 mM $NaNO_3$, at –0.6 V.

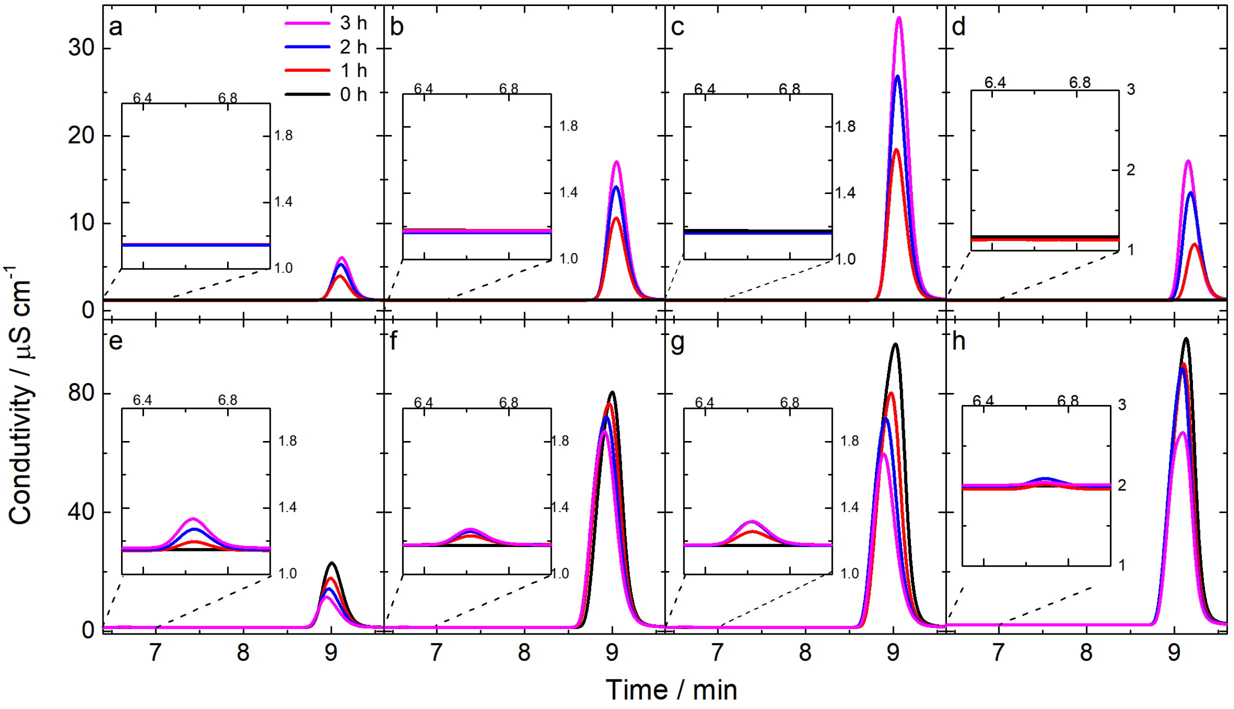

Figure S30. IC curves used to quantify NO_3^- from the chronoamperometric experiments conducted based on the application of the Co_3O_4(Cowt.%55)GNR catalyst in Ar–saturated 0.1 M K_2SO_4 in the presence of (a) 10, (b) 50, (c) 70, and (d) 100 mM $NaNO_3$ concentration (cell anodic branch, aliquot of 500, 500, 400, and 250 µL, respectively) and (e) 10, (f) 50, (g) 70, and (h) 100 mM $NaNO_3$concentration (cell cathodic branch, aliquot of 500, 500, 400, and 250 µL, respectively); applied potential: –0.6 V.

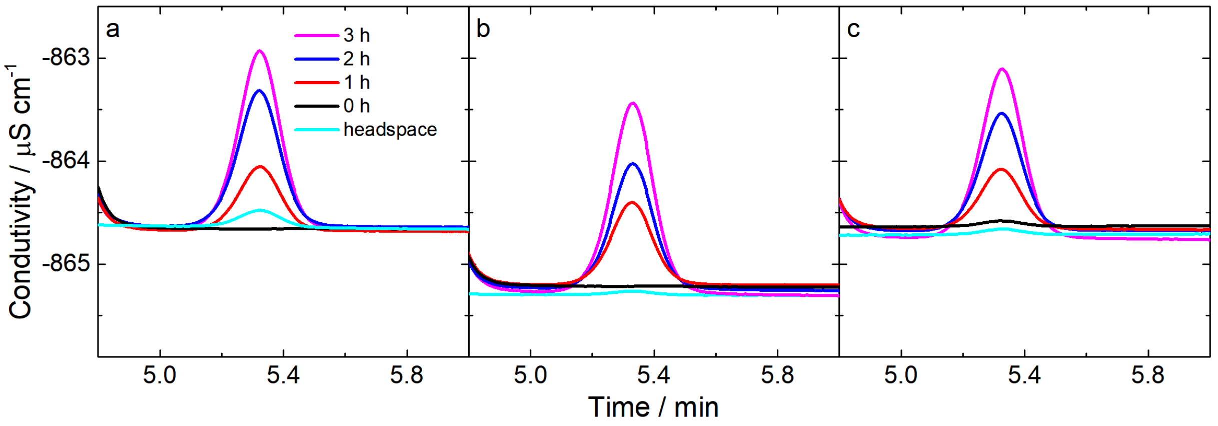

Figure S31. IC curves used to quantify NH_4^+ from the chronoamperometric experiments conducted based on the application of the Co_3O_4(Cowt.%55)GNR catalyst in Ar–saturated 0.1 M K_2SO_4, in the presence of 40 mM $NaNO_3$, at the following applied potentials: (a) –0.5, (b) –0.6, and (c) –0.7 V (cell cathodic branch, aliquot of 100 µL).

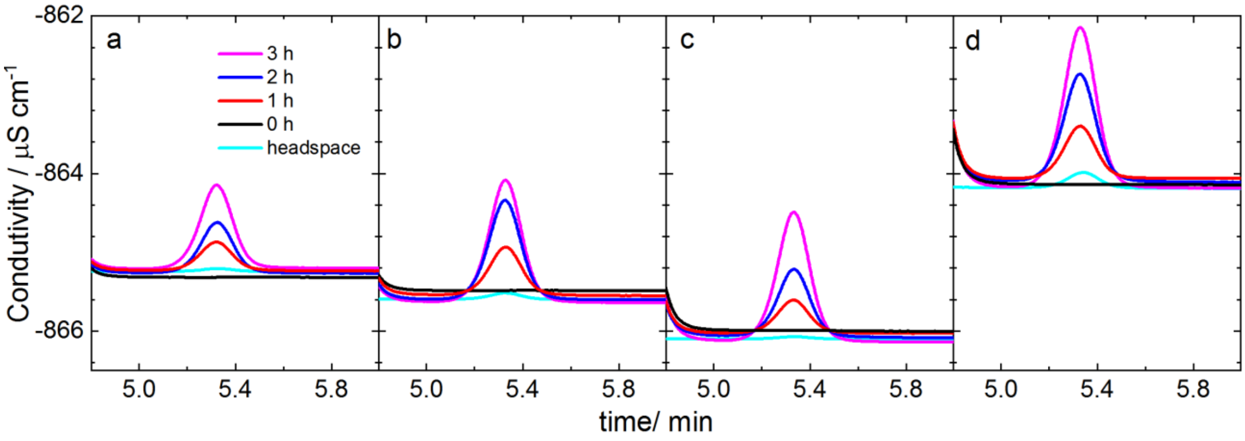

Figure S32. IC curves used to quantify NH_4^+ from the chronoamperometric experiments conducted based on the application of the Co_3O_4(Cowt.%55)GNR catalyst in Ar–saturated 0.1 M K_2SO_4 in the presence of (a) 20, (b) 30, (c) 60, and (d) 70 mM $NaNO_3$ concentration (cell cathodic branch, aliquot of 100 µL); applied potential:–0.6 V.

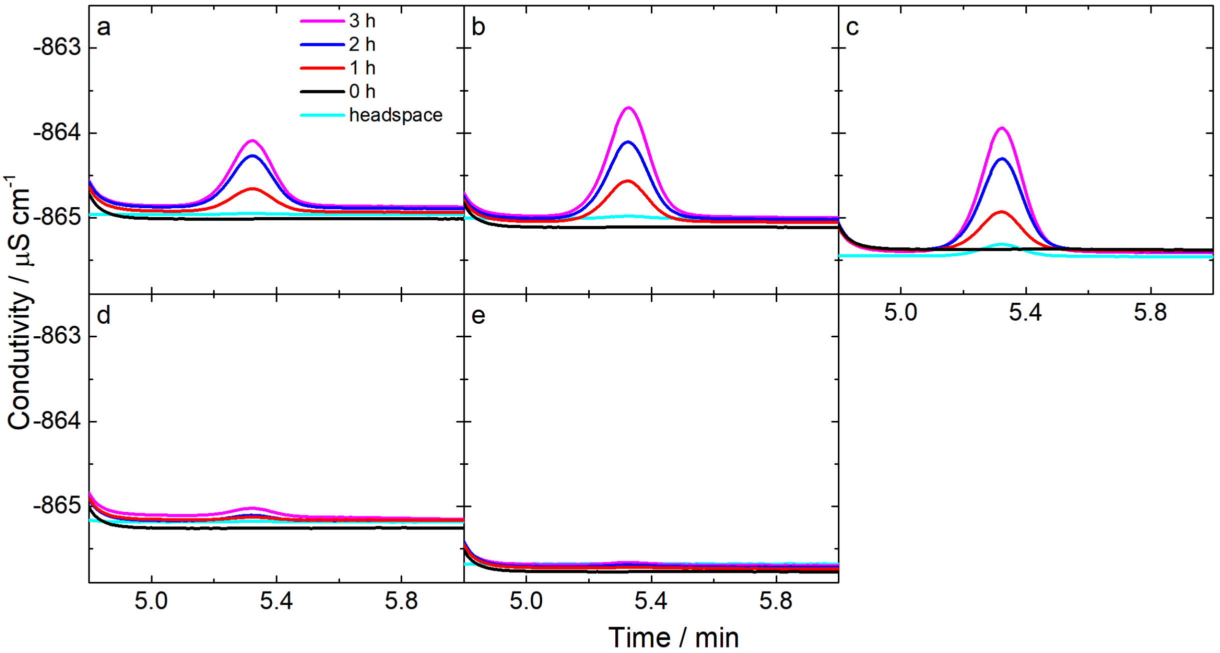

Figure S33. IC curves used to quantify NH_4^+ from the chronoamperometric experiments conducted based on the application of the (a) Co_3O_4(Cowt.%53)GNR, (b) Co_3O_4(Cowt.%38)GNR, (c) bare Co_3O_4(Cowt.%75), (d) bare GNR(Cowt.%0), (e) bare CP (cell cathodic branch, aliquot of 100 µL) catalysts in Ar–saturated 0.1 M K_2SO_4, in the presence of 40 mM $NaNO_3$; applied potential: –0.6 V.

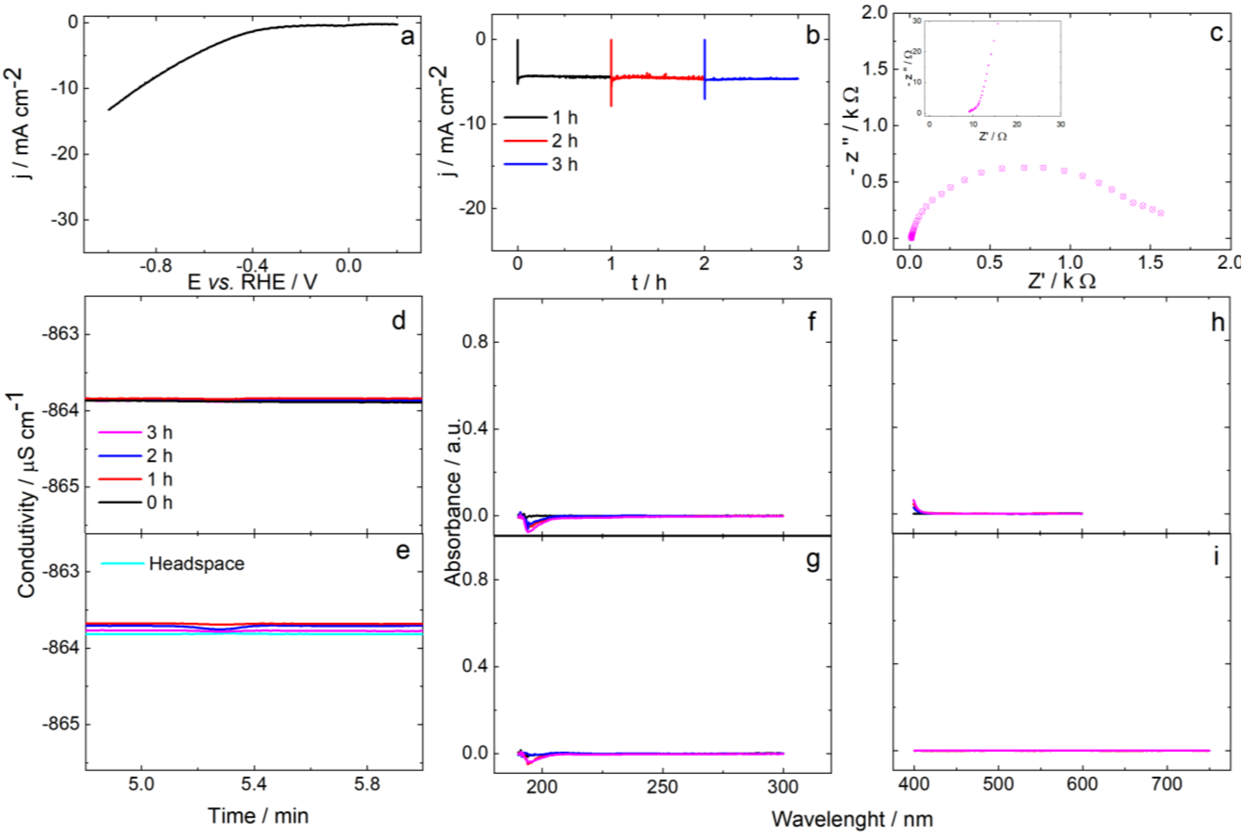

Figure S34. Responses obtained for the CP electrode modified with 37.5 µg cm^{-2} of Co_3O_4(Cowt.%55)GNR employed in Ar–saturated 0.1 M K_2SO_4. (a) stationary linear sweep voltammogram (LSV) recorded at ν = 5 mV s^{-1}. Scan was initiated at 0.2 V; (b) chronoamperometric results (–0.6 V). After each one hour of chronoamperometric experiment, the experiment was interrupted in order to remove aliquots from the solution; (c) EIS result (Nyquist plot); IC curves used to quantify NH_4^+ in the cell anodic (d) and cathodic (e) branches (aliquots of 100 µL) during the chronoamperometric experiments conducted at the potential of –0.6 V; UV curves used to quantify NO_3^- in the cell anodic (f, 60 µL) and cathodic (g, 20 µL) branches during the chronoamperometric experiments conducted at the potential of –0.6 V; UV-visible curves used to quantify (h) hydrazine in the cell cathodic branch (300 µL), and (i) NO_2^- in the cell cathodic branch (200 µL)during the chronoamperometric experiments conducted at the potential of –0.6 V (both).

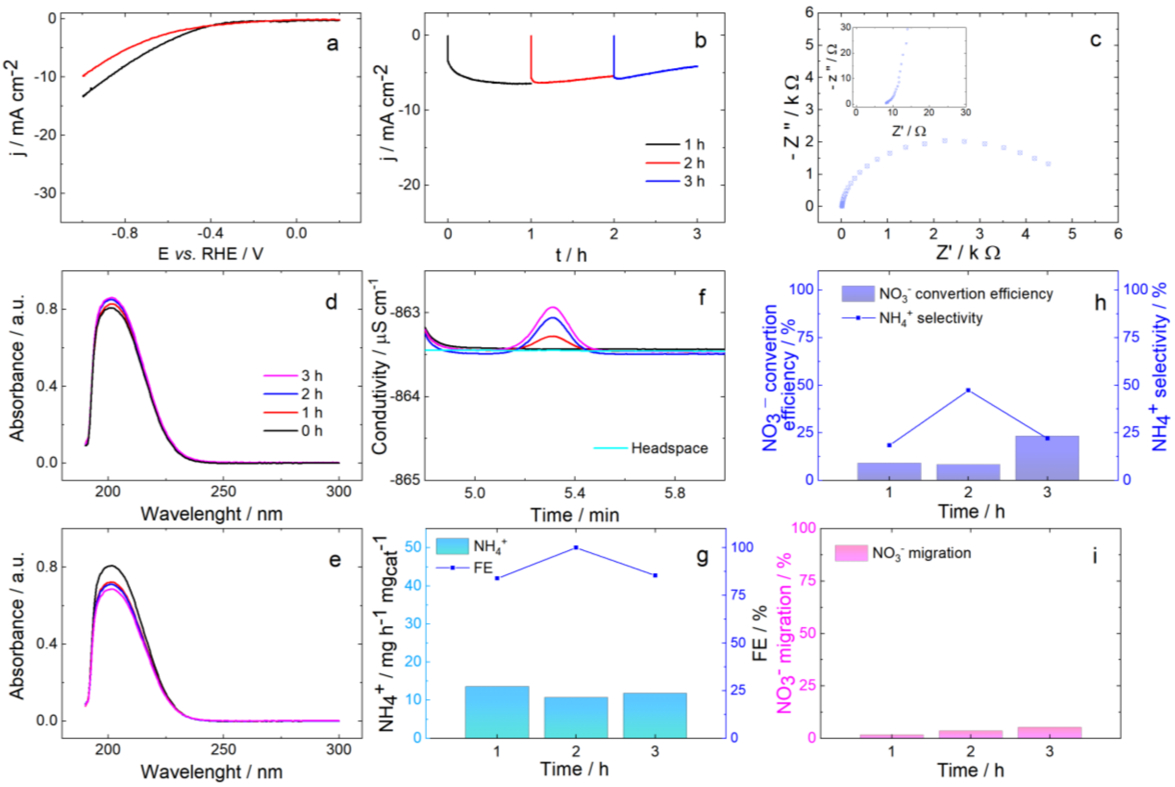

Figure S35. Responses obtained for the CP electrode modified with 37.5 µg cm^{-2} of Co_3O_4(Cowt.%55)GNR: (a) stationary linear sweep voltammograms (LSVs) recorded at ν = 5 mV s^{-1} using Ar–saturated 0.1 M K_2SO_4 (black line) as supporting electrolyte in the presence of 40 mM $NaNO_3$ (red line) in both the H-cell anodic and cathodic branches. Scans were initiated at 0.2 V; (b) chronoamperometric result (–0.6 V) obtained from the application of 0.1 M K_2SO_4 as electrolyte solution in the presence of 40 mM $NaNO_3$ in both the H-cell anodic and cathodic branches. After each one hour of chronoamperometric experiment, the experiment was interrupted in order to remove aliquots from the solution; (c) EIS result (Nyquist plot) obtained from the application of 0.1 M K_2SO_4 as supporting electrolyte in the presence of 40 mM $NaNO_3$ in both the H-cell anodic and cathodic branches; UV curves used to quantify NO_3^- in the H-cell anodic (d, 60 µL) and cathodic (e, 20 µL) branches during the chronoamperometric experiments conducted at –0.6 V; (f) IC curves used to quantify NH_4^+ (100 µL) in the H-cell cathodic branch during the chronoamperometric experiments conducted at –0.6 V; (h) NO_3^- conversion efficiency and NH_4^+ selectivity, (i) NO_3^- migration, and (g) NH_4^+ yield rate and FE values obtained after three hours of chronoamperometric experiments conducted based on the application of the Co_3O_4(Cowt.%55)GNR catalyst in Ar–saturated 0.1 M K_2SO_4, in the presence 40 mM $NaNO_3$, at the potential of –0.6 V.

The nitrate migration (%) from the H-cell cathodic branch to the anodic branch, in the presence of initially equal NO_3^- moles in both the H-cell anodic and cathodic branches was determined by:

NO_3^- migration (%) = $\frac{(moles_{NO_3^-,t} - moles_{NO_3^-,initial})\ cell\ anodic\ branch}{moles_{NO_3^-,initial,\ cell\ anodic\ branch}} \times 100$ (S1)

where $moles_{NO_3^-,initial,\ cell\ anodic\ branch}$ stands for the nitrate moles in the H-cell anodic branch at time zero, and $moles_{NO_3^-,t,\ cell\ anodic\ branch}$ is the nitrate moles in the H-cell anodic branch at time t.

The nitrate conversion efficiency (%) of initially equal NO_3^- moles in both the H-cell anodic and cathodic branches was determined by:

NO_3^- conversion efficiency (%) $= \frac{(moles_{NO_3^-,initial} - moles_{NO_3^-,t})cell\ cathodic\ branch - (moles_{NO_3^-,t} - moles_{NO_3^-,initial})cell\ anodic\ branch}{moles_{NO_3^-,initial,\ cell\ cathodic\ branch}} \times 100$(S2)

where $moles_{NO_3^-,initial,\ cell\ cathodic\ branch}$ stands for the nitrate moles in the H-cell cathodic branch at time zero, and $moles_{NO_3^-,t,\ cell\ cathodic\ branch}$ is the nitrate moles that remained in the H-cell cathodic branch at time t.

The NH_4^+ selectivity (%) of initially equal NO_3^- moles in both the H-cell anodic and cathodic branches was determined by:

NH_4^+ selectivity (%) $= \frac{moles_{NH_4^+,t}}{(moles_{NO_3^-,initial} - moles_{NO_3^-,t})cell\ cathodic\ branch - (moles_{NO_3^-,t} - moles_{NO_3^-,initial})cell\ anodic\ branch} \times 100$ (S3)

where $[NH_4^+]_t$ is the ammonia moles at time t.

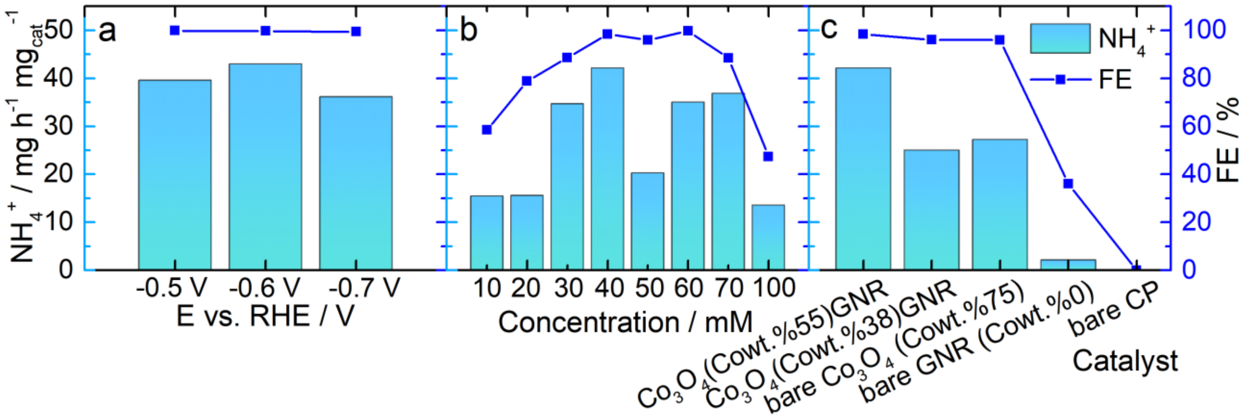

Figure 36. Average NH_4^+ yield rate and FE values obtained in three hours of chronoamperometry experiments conducted based on the application of: (a) the Co_3O_4(Cowt.%55)GNR catalyst in Ar–saturated 0.1 M K_2SO_4 in the presence of 40 mM $NaNO_3$ at different applied potentials; (b) the Co_3O_4(Cowt.%55)GNR catalyst in Ar–saturated 0.1 M K_2SO_4 in the presence of varying $NaNO_3$ concentrations at –0.6 V vs.

RHE; and (c) for other different catalysts in Ar–saturated 0.1 M K_2SO_4 in the presence of 40 mM $NaNO_3$ at –0.6 V vs. RHE. The quantification values were based on the results obtained from UV-visible and IC (Figures S27-S29 and S31-33) and chronoamperometric experiments (Figures S18-20).

Tabel S9. Average NH_4^+ yield rates obtained after three hours of chronoamperometry experiments conducted at –0.6 V vs. RHE using the following: Co_3O_4(Cowt.%55)GNR catalyst employed in Ar–saturated 0.1 M K_2SO_4 containing 40 mM $NaNO_3$;Co_3O_4(Cowt.%55)GNR catalyst employed in Ar–saturated 0.1 M K_2SO_4, in the presence of varying $NaNO_3$ concentrations; and for different catalysts employed in Ar–saturated 0.1 M K_2SO_4 containing 40 mM $NaNO_3$. Quantification obtained from UV-visible and IC results (Figures S27-S29 and S31-33).

Potential (V vs. RHE)	µmol h^{-1} cm^{-2}	mg h^{-1} cm^{-2}	mg $h^{-1}mg_{cat}^{-1}$	mmol $h^{-1}mg_{cat}^{-1}$
–0.5	82.49	1.48	39.58	2.20
–0.6	87.79	1.58	42.11	2.34
–0.7	75.35	1.35	36.15	2.01
**NO_3^- concentration (mM)**	**µmol h^{-1} cm^{-2}**	**mg h^{-1} cm^{-2}**	**mg $h^{-1}mg_{cat}^{-1}$**	**mmol $h^{-1}mg_{cat}^{-1}$**
10	32.25	0.58	15.46	0.86
20	32.52	0.58	15.59	0.87
30	72.30	1.30	34.68	1.93
40	87.79	1.58	42.11	2.34
50	42.34	0.76	20.30	1.129
60	73.05	1.31	35.03	1.95
70	76.87	1.38	36.87	2.05
100	28.23	0.51	13.55	0.75
**Catalysts**	**µmol h^{-1} cm^{-2}**	**mg h^{-1} cm^{-2}**	**mg $h^{-1}mg_{cat}^{-1}$**	**mmol $h^{-1}mg_{cat}^{-1}$**
Co_3O_4(Cowt.%53)GNR	41.55	0.75	19.94	1.11
Co_3O_4(Cowt.%55)GNR	87.79	1.58	42.11	2.34
Co_3O_4(Cowt.%38)GNR	52.13	0.94	25.00	1.39
bare Co_3O_4(Cowt.%75)	56.72	1.02	27.21	1.51
bare GNR(Cowt.%0)	4.44	0.08	2.13	0.12
bare CP	0	0	0	0

Table S10. Overview of catalysts, potentials, nitrate conversion efficiency, ammonia yields or production rates, Faradaic efficiencies, ammonia selectivity, and electrolyte solutions involved in the electrochemical reduction of nitrate.

**Catalyst; loading**	**Potential**	**Nitrate conversion efficiency (%)**	**Ammonia yield or production rate**	**Faradaic efficiency (%)**	**Ammonia selectivity (%)**	**Electrolyte; nitrate concentration**	**Reference**
Co_3O_4(Co wt.%55)GNR; 37.5 µg cm^{-2}	−0.6 V vs. RHE	14.7	42.11 mg $h^{-1}mg_{cat}^{-1}$ or 87.79 µmol h^{-1} cm^{-2} or 2.34 mmol $h^{-1}mg_{cat}^{-1}$ or 1.58 mg h^{-1} cm^{-2}	98.7	49.95	0.1 M K_2SO_4; 40mMNaNO_3	This work
Co-NAs; 1,600µg cm^{-2}	−0.24 V vs. RHE<br>−0.6 V vs. RHE	–	10.4 mmol h^{-1} cm^{-2}<br>3.1 mmol h^{-1} cm^{-2}	≥97%<br>≥96%	–	1 M KOH;0.1 M NO_3^-.0.5 M Na_2SO_4;0.1 M NO_3^-	[10]
Co_2AlO_4/CC; –	-	–	7.9mg $h^{-1}cm^{-2}$ at −0.9 V vs. RHE	92.6 at −0.7 V vs. RHE	–	0.1 M PBS; 0.1 M NO_3^-	[11]
Core-shellCu/CuO_x and Co/CoO phases;–	−0.175 V vs. RHE	–	1.17 mmol h^{-1} cm^{-2}	90.6	–	0.1 M KOH; 0.1 M NO_3^-	[12]
CoO_x nanosheets; 35 µg cm^{-2}	−0.3 V vs. RHE	–	82.4 ± 4.8 mg h^{-1} mg_{cat}^{-1} (36.62 mg h^{-1} cm^{-2})	93.4 ± 3.8	–	0.1 M KOH; 0.1 M KNO_3	[13]
Co/CoO; probablyaround 370µg cm^{-2}	−1.3 V vs. SCE	–	194.46 µmol h^{-1} cm^{-2}	93.8	91.2	0.1 M Na_2SO_4; 200 ppmNO_3^-	[14]

Co-P/Ti; –	−0.3 V vs. RHE	86.9 (10 h)	416.0 ±7.2 mg h^{-1} cm^{-2} at −0.6 V vs. RHE	93.6 ± 3.3	–	0.2 M Na_2SO_4; 200 ppm NO_3^-	15
Co_3O_4-TiO_2/Ti; –	Currentdensity = 10 mA cm^{-2}	~80	–	–	100% of N_2 selectivity with Cl^-conc. of 2000 mg L^{-1}	0.1 M Na_2SO_4; 50 mg L^{-1} NO_3^-	16
CoP PANSs; 114.3 µg cm^{-2}	−0.5 V vs. RHE	–	19.28 ± 0.53 mg h^{-1} mg_{cat}^{-1} (2.204 mg h^{-1} cm^{-2})	94.24 ± 2.8	–	0.5 M K_2SO_4; 0.05 M KNO_3	17
Ni/Cu_2O/Co$(OH)_x$; –	current density = 40 mA cm^{-2}	90.3	1.22 mmol NH_3 g_{cat}^{-1} h^{-1}; electrical energy per order = 8 kWh L^{-1} $order^{-1}$	22	94.0	12.5 mmol L^{-1} Na_2SO_4; 30 mg L^{-1} NO_3^-–N	18
MR Co-NC; 600 µg cm^{-2}	−0.7 V vs. RHE	–	1.25 ± 0.023 mmol h^{-1} cm^{-2}, 35.42 ± 0.65 mg_{NH3} $h^{-1}mg_{cat}^{-1}$, 2.08 ± 0.038 mol $h^{-1}g_{cat}^{-1}$	95.35 ± 1.75	–	0.1 M KOH; 0.1 M KNO_3	19
Co_3O_4/CF; –	Currentdensity = 5 mA cm^{-2}	72.9	–	–	96.2% of N_2 selectivity	50 mM Na_2SO_4;50 mg N/L NO_3^-	20
Co_3O_4/CF; 6,400 µg cm^{-2}	−1.3 V vs Ag/AgCl	NO_3^-removal (91%)	283 µg mg^{-1} h^{-1}	22.19	82.1	50 mM Na_2SO_4; 50 mg L^{-1} NO_3^-	21
Cu-Co_3O_4/CC	−0.6 V vs. RHE	~100	36.71 mmol $h^{-1}g^{-1}$	86.5	~95	0.1 M Na_2SO_4;5	22

; –						00 ppm NO_3^-	
Co_3O_4/CC with cobalt vacancies (v_{Co}-Co_3O_4/CC); –	−0.6 V vs. RHE	–	517.5 μmol h^{-1} cm^{-2}	~93	high	0.1 M NaOH; 0.1 M NO_3^-	[23]
$(Cu_{0.6}Co_{0.4})Co_2O_4$; 1,000 μg cm^{-2}	−0.45 V vs. RHE	–	1.09 mmol h^{-1} cm^{-2}	96.5	–	1.0 M KOH; 0.1 M NO_3^-	[24]
S-Co_3O_4; 400 μg cm^{-2}	−0.60 V vs. RHE	–	174.2 mmol h^{-1} g^{-1}; 314.5 mmol h^{-1} g^{-1}	89.9; 87.6	–	0.1 M Na_2SO_4; 0.1 M $NaNO_3$; 0.1 M $NaNO_2$	[25]
O_v-Co_3O_4 NPs; 1,000 μg cm^{-2}	−0.60 V vs. RHE	90.76% of NO_x^- conversion	24.92 mg h^{-1} cm^{-2}	93.77	98.75% of NO_2^- selectivity	1 M NaOH;0.1 1 M NO_x^-	[26]
Cu_2O+Co_3O_4 on CP; 500 μg cm^{-2}	−0.30 V vs. RHE	–	12.76 mg h^{-1} cm^{-2}	85.4	~98	0.1 M NaOH; 0.1 M $NaNO_3$	[27]
Co_2NiO_4; –	−1.20 V vs. RHE	–	27 mg h^{-1} cm^{-2}	94.9 at −1.00 V vs. RHE	–	0.5 M K_2SO_4; 0.1 M KNO_3	[28]
Co_3O_4@CNF; 100 μg cm^{-2}	−0.70 V vs. RHE	–	23.4 mg $h^{-1}mg^{-1}_{cat}$	92.7	–	0.1 M PBS; 0.1 M $NaNO_3$	[29]

CoP PANSs = porous and amorphous cobalt phosphide nanoshuttles.

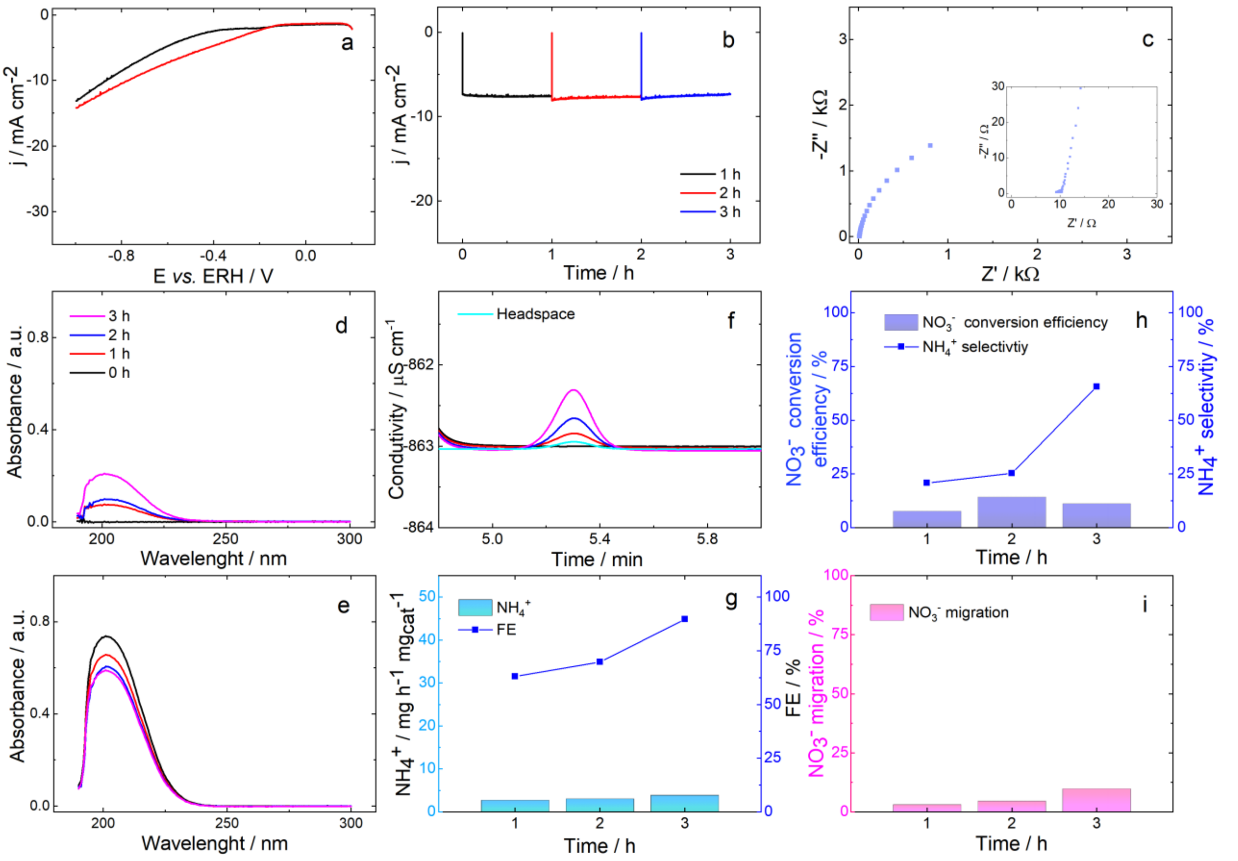

Figure S37. Responses obtained for the CP electrode modified with 150 µg cm^{-2} of Co_3O_4(Cowt.%55)GNR: (a) stationary linear sweep voltammograms (LSVs) recorded at ν = 5 mV s^{-1} using Ar–saturated 0.1 M K_2SO_4 (black line) as supporting electrolyte in the presence of 40 mM $NaNO_3$ (red line) in the H-cell cathodic branch. Scans were initiated at 0.2 V; (b) chronoamperometric result (–0.6 V) obtained from the application of 0.1 M K_2SO_4 as electrolyte solution in the presence of 40 mM $NaNO_3$ in the H-cell cathodic branch. After each one hour of chronoamperometric experiment, the experiment was interrupted in order to remove aliquots from the solution; (c) EIS result (Nyquist plot) obtained from the application of 0.1 M K_2SO_4 as supporting electrolyte in the presence of 40 mM $NaNO_3$ in the H-cell cathodic branch; UV curves used to quantify NO_3^- in the H-cell anodic (d, 60 µL) and cathodic (e, 20 µL) branches during the chronoamperometric experiments conducted at –0.6 V; (f) IC curves used to quantify NH_4^+ (100 µL) in the H-cell cathodic branch during the chronoamperometric experiments conducted at –0.6 V; (h) NO_3^- conversion efficiency and NH_4^+ selectivity, (i) NO_3^- migration, and (g) NH_4^+ yield rate and FE values obtained after three hours of chronoamperometric experiments conducted based on the application of the Co_3O_4(Cowt.%55)GNR catalyst in Ar–saturated 0.1 M K_2SO_4, in the presence 40 mM $NaNO_3$, at the potential of –0.6 V.

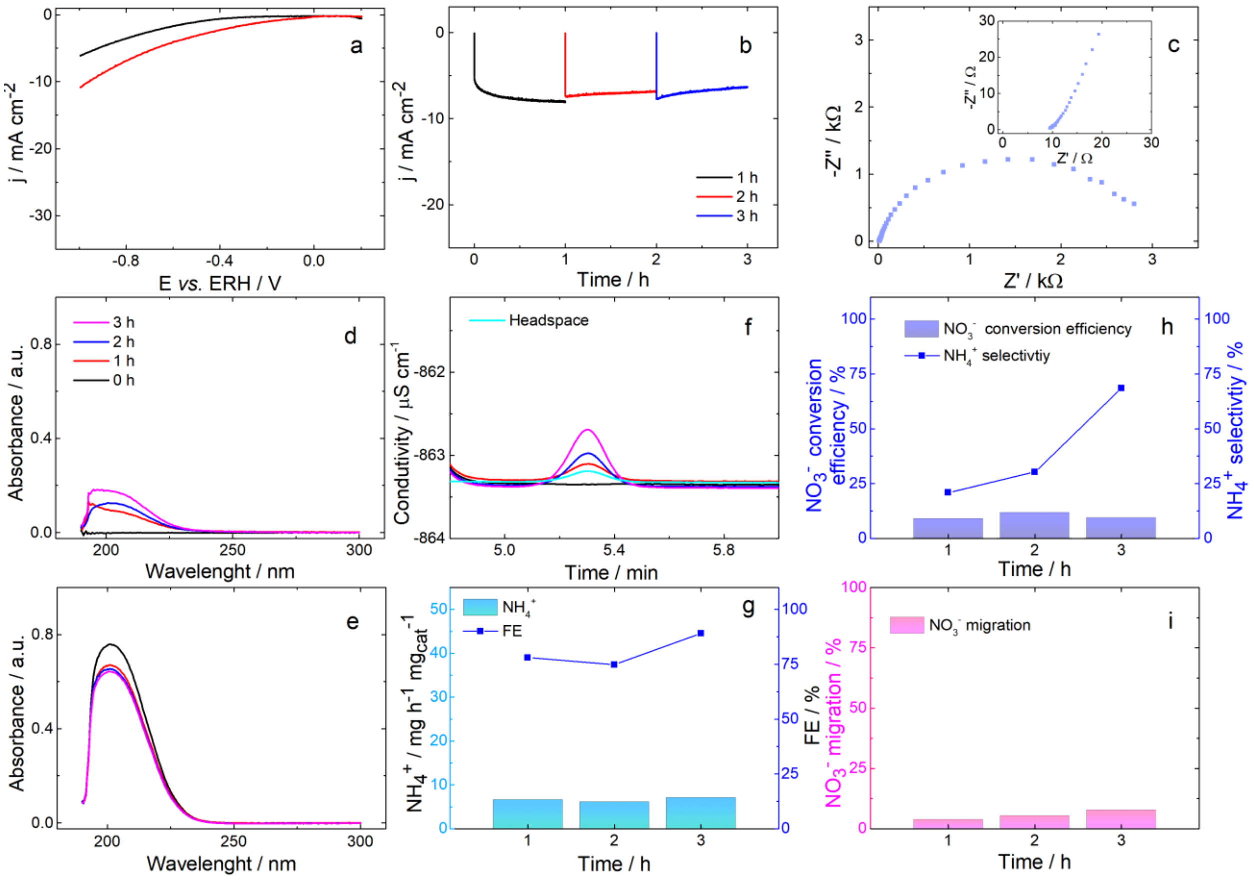

Figure S38. Responses obtained for the CP electrode modified with 75 µg cm^{-2} of Co_3O_4(Cowt.%55)GNR: (a) stationary linear sweep voltammograms (LSVs) recorded at ν = 5 mV s^{-1} using Ar–saturated 0.1 M K_2SO_4 (black line) as supporting electrolyte in the presence of 40 mM $NaNO_3$ (red line) in the H-cell cathodic branch. Scans were initiated at 0.2 V; (b) chronoamperometric result (–0.6 V) obtained from the application of 0.1 M K_2SO_4 as electrolyte solution in the presence of 40 mM $NaNO_3$ in the H-cell cathodic branch. After each one hour of chronoamperometric experiment, the experiment was interrupted in order to remove aliquots from the solution; (c) EIS result (Nyquist plot) obtained from the application of 0.1 M K_2SO_4 as supporting electrolyte in the presence of 40 mM $NaNO_3$ in the H-cell cathodic branch; UV curves used to quantify NO_3^- in the H-cell anodic (d, 60 µL) and cathodic (e, 20 µL) branches during the chronoamperometric experiments conducted at –0.6 V; (f) IC curves used to quantify NH_4^+ (100 µL) in the H-cell cathodic branch during the chronoamperometric experiments conducted at –0.6 V; (h) NO_3^- conversion efficiency and NH_4^+ selectivity, (i) NO_3^- migration, and (g) NH_4^+ yield rate and FE values obtained after three hours of chronoamperometric experiments conducted based on the application of the Co_3O_4(Cowt.%55)GNR catalyst in Ar–saturated 0.1 M K_2SO_4, in the presence of 40 mM $NaNO_3$, at the potential of –0.6 V.

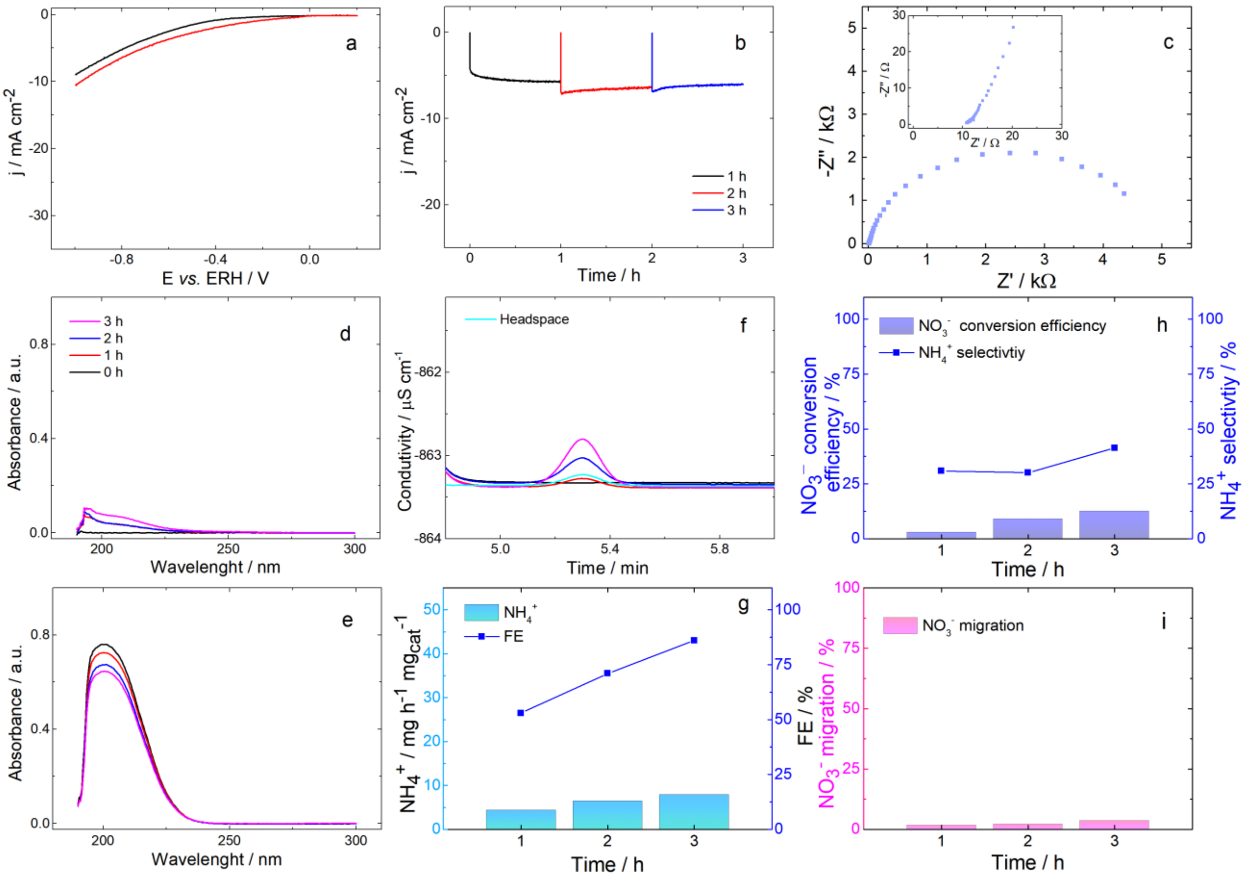

Figure S39. Responses obtained for the CP electrode modified with 56 µg cm^{-2} of Co_3O_4(Cowt.%55)GNR: (a) stationary linear sweep voltammograms (LSVs) recorded at ν = 5 mV s^{-1} using Ar–saturated 0.1 M K_2SO_4 (black line) as supporting electrolyte in the presence of 40 mM $NaNO_3$ (red line) in the H-cell cathodic branch. Scans were initiated at 0.2 V; (b) chronoamperometric result (–0.6 V) obtained from the application of 0.1 M K_2SO_4 as electrolyte solution in the presence of 40 mM $NaNO_3$ in the H-cell cathodic branch. After each one hour of chronoamperometric experiment, the experiment was interrupted in order to remove aliquots from the solution; (c) EIS result (Nyquist plot) obtained from the application of 0.1 M K_2SO_4 as supporting electrolyte in the presence of 40 mM $NaNO_3$ in the H-cell cathodic branch; UV curves used to quantify NO_3^- in the H-cell anodic (d, 60 µL) and cathodic (e, 20 µL) branches during the chronoamperometric experiments conducted at –0.6 V; (f) IC curves used to quantify NH_4^+ (100 µL) in the H-cell cathodic branch during the chronoamperometric experiments conducted at –0.6 V; (h) NO_3^- conversion efficiency and NH_4^+ selectivity, (i) NO_3^- migration, and (g) NH_4^+ yield rate and FE values obtained after three hours of chronoamperometric experiments conducted based on the application of the Co_3O_4(Cowt.%55)GNR catalyst in Ar–saturated 0.1 M K_2SO_4, in the presence of 40 mM $NaNO_3$, at the potential of –0.6 V.

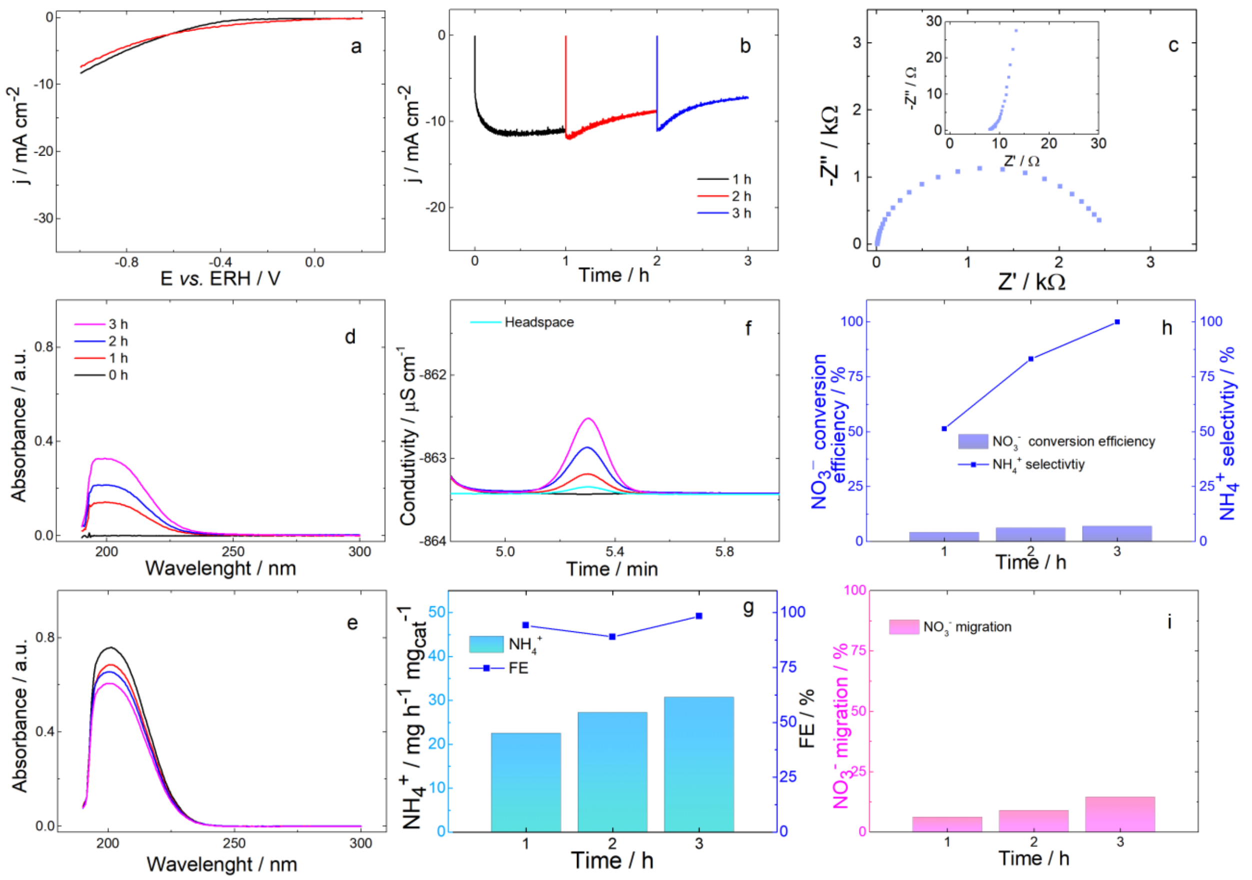

Figure S40. Responses obtained for the CP electrode modified with 19 µg cm^{-2} of Co_3O_4(Cowt.%55)GNR: (a) stationary linear sweep voltammograms (LSVs) recorded at ν = 5 mV s^{-1} using Ar–saturated 0.1 M K_2SO_4 (black line) as supporting electrolyte in the presence of 40 mM $NaNO_3$ (red line) in the H-cell cathodic branch. Scans were initiated at 0.2 V; (b) chronoamperometric result (–0.6 V) obtained from the application of 0.1 M K_2SO_4 as electrolyte solution in the presence of 40 mM $NaNO_3$ in the H-cell cathodic branch. After each one hour of chronoamperometric experiment, the experiment was interrupted in order to remove aliquots from the solution; (c) EIS result (Nyquist plot) obtained from the application of 0.1 M K_2SO_4 as supporting electrolyte in the presence of 40 mM $NaNO_3$ in the H-cell cathodic branch; UV curves used to quantify NO_3^- in the H-cell anodic (d, 60 µL) and cathodic (e, 20 µL) branches during the chronoamperometric experiments conducted at –0.6 V; (f) IC curves used to quantify NH_4^+ (100 µL) in the H-cell cathodic branch during the chronoamperometric experiments conducted at –0.6 V; (h) NO_3^- conversion efficiency and NH_4^+ selectivity, (i) NO_3^- migration, and (g) NH_4^+ yield rate and FE values obtained after three hours of chronoamperometric experiments conducted based on the application of the Co_3O_4(Cowt.%55)GNR catalyst in Ar–saturated 0.1 M K_2SO_4, in the presence of 40 mM $NaNO_3$, at the potential of –0.6 V.

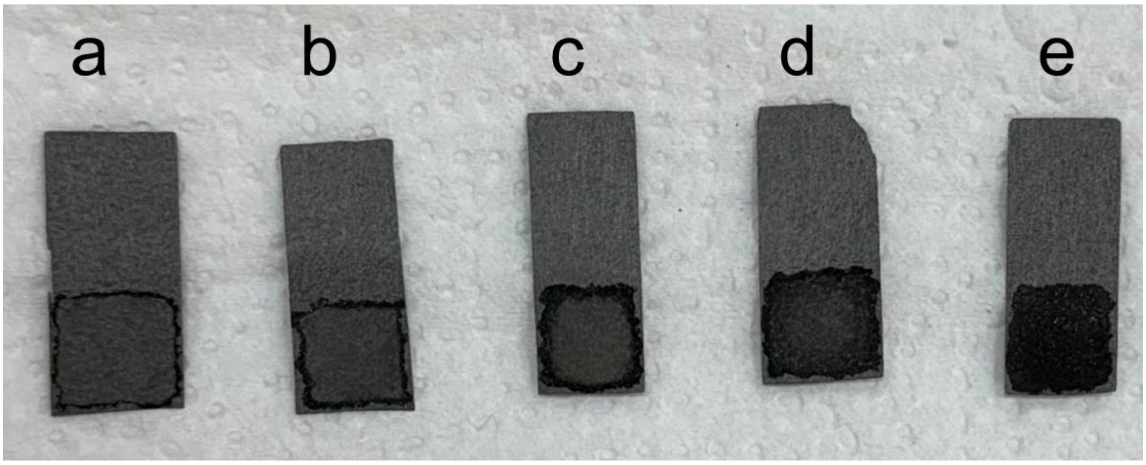

Figure S41. CP modified with (a) 19, (b)37.5, (c)56, (d) 75, and (e) 150 µg cm^{-2} of Co_3O_4(Cowt.%55)GNR.

Table S11. The R_S and R_{ct} values obtained for the CP electrodes modified with different loadings of the Co_3O_4(Cowt.%55)GNR catalyst employed in Ar–saturated 0.1 M K_2SO_4 in the presence of 40 mM $NaNO_3$. The R_S and R_{ct} values were obtained from the EIS results (data shown in Figures S37c-S40c and S43c). The solution pH (0.1 M K_2SO_4 in the presence of 40 mM $NaNO_3$) is reported below before and after the chronoamperometry experiments.

**Loading of Co_3O_4(Cowt.%55)GNR / µg cm^{-2}**	**R_S / Ω**	**R_{ct} / kΩ**	**Solution pH before chronoamperometry**	**Solution pH after chronoamperometry**
**19**	9.1	2.6	7.24	12.51
**37.5 (H-cell with Nafion 117 membrane)**	6.9	1.3	8.38	12.97
**56**	12.1	5.1	7.83	11.59
**75**	10.6	2.9	6.85	11.73
**150**	10.5	2.3	8.80	13.39

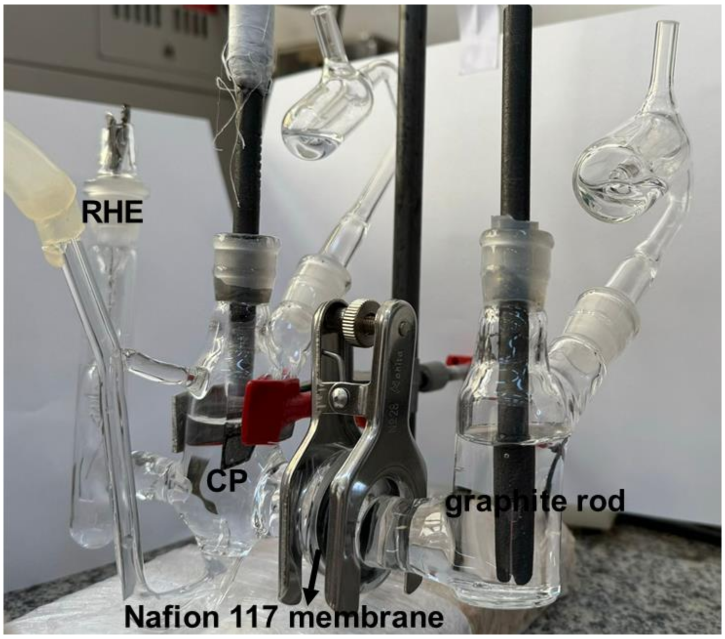

Figure S42. H-cell image with cathodic and anodic branches separated by Nafion 117 membrane. The distance between the working electrode (modified CP) and the counter electrode (graphite rod) was 8.5 cm.

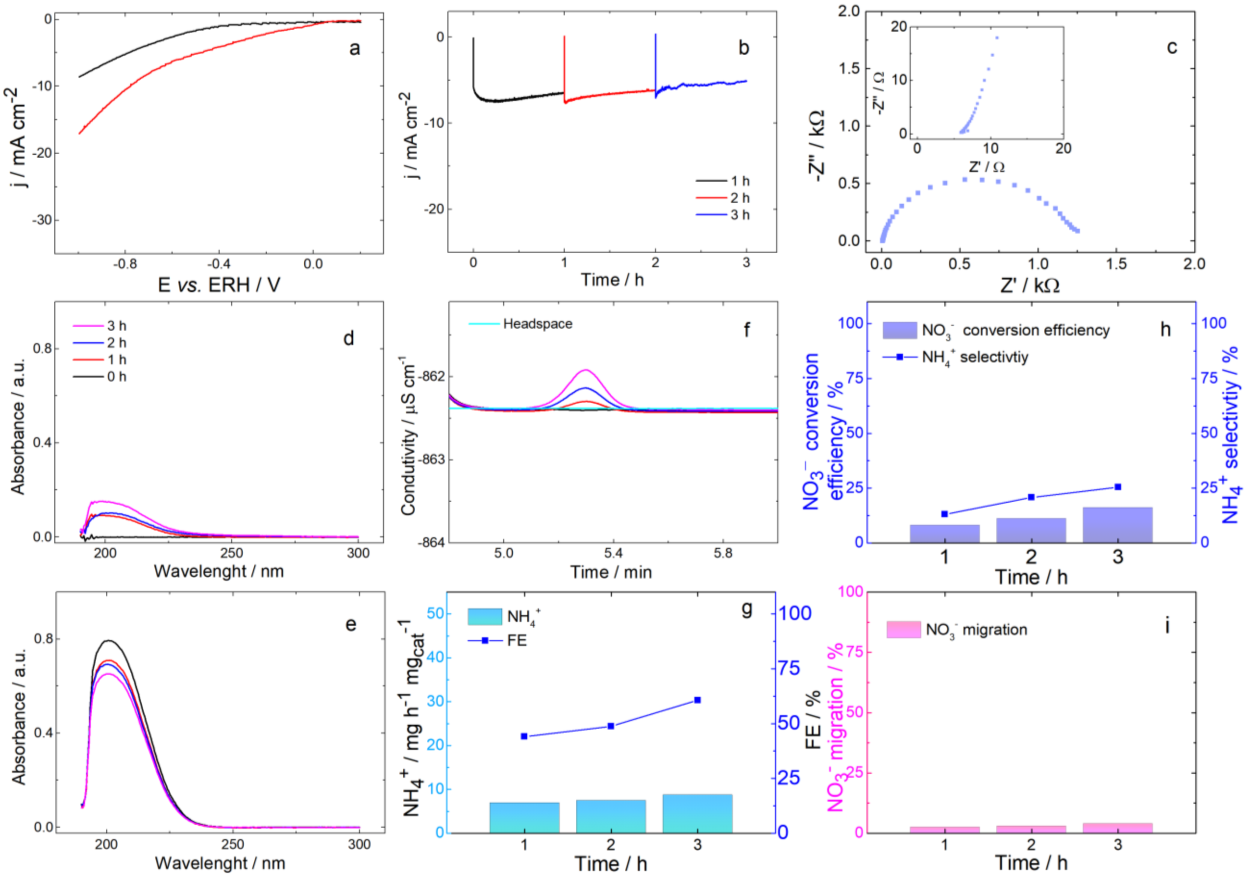

Figure S43. Responses obtained for the CP electrode modified with 37.5 µg cm^{-2} of Co_3O_4(Cowt.%55)GNR: (a) stationary linear sweep voltammograms (LSVs) recorded at

ν = 5 mV s^{-1} using Ar–saturated 0.1 M K_2SO_4 (black line) as supporting electrolyte in the presence of 40 mM $NaNO_3$ (red line) in the H-cell (separated by Nafion 117 membrane) cathodic branch. Scans were initiated at 0.2 V; (b) chronoamperometric result (–0.6 V) obtained from the application of 0.1 M K_2SO_4 as electrolyte solution in the presence of 40 mM $NaNO_3$ in the H-cell cathodic branch. After each one hour of chronoamperometric experiment, the experiment was interrupted in order to remove aliquots from the solution; (c) EIS result (Nyquist plot) obtained from the application of 0.1 M K_2SO_4 as supporting electrolyte in the presence of 40 mM $NaNO_3$ in the H-cell cathodic branch; UV curves used to quantify NO_3^- in the H-cell anodic (d, 60 µL) and cathodic (e, 20 µL) branches during the chronoamperometric experiments conducted at –0.6 V; (f) IC curves used to quantify NH_4^+ (100 µL) in the H-cell cathodic branch during the chronoamperometric experiments conducted at –0.6 V; (h) NO_3^- conversion efficiency and NH_4^+ selectivity, (i) NO_3^- migration, and (g) NH_4^+ yield rate and FE values obtained after three hours of chronoamperometric experiments conducted based on the application of the Co_3O_4(Cowt.%55)GNR catalyst in Ar–saturated 0.1 M K_2SO_4, in the presence 40 mM $NaNO_3$, at the potential of –0.6 V.

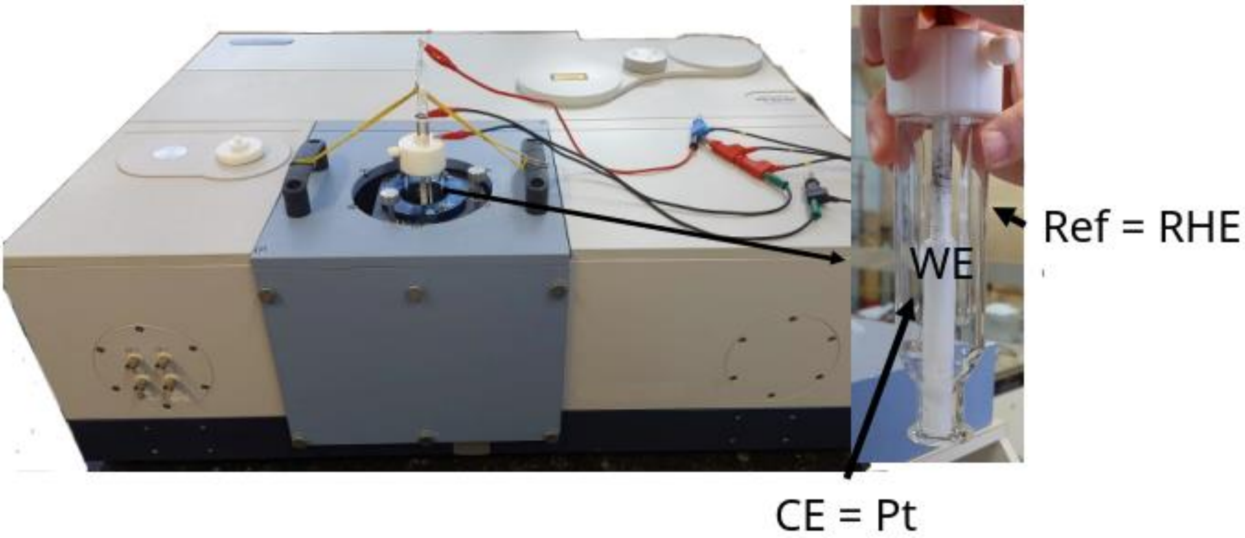

Figure S44. Images of the in-situ FTIR equipment and electrochemical cell used for the conduct of the experiments where the working electrode (WE), reference electrode (Ref = RHE), and counter electrode (CE) are identified.

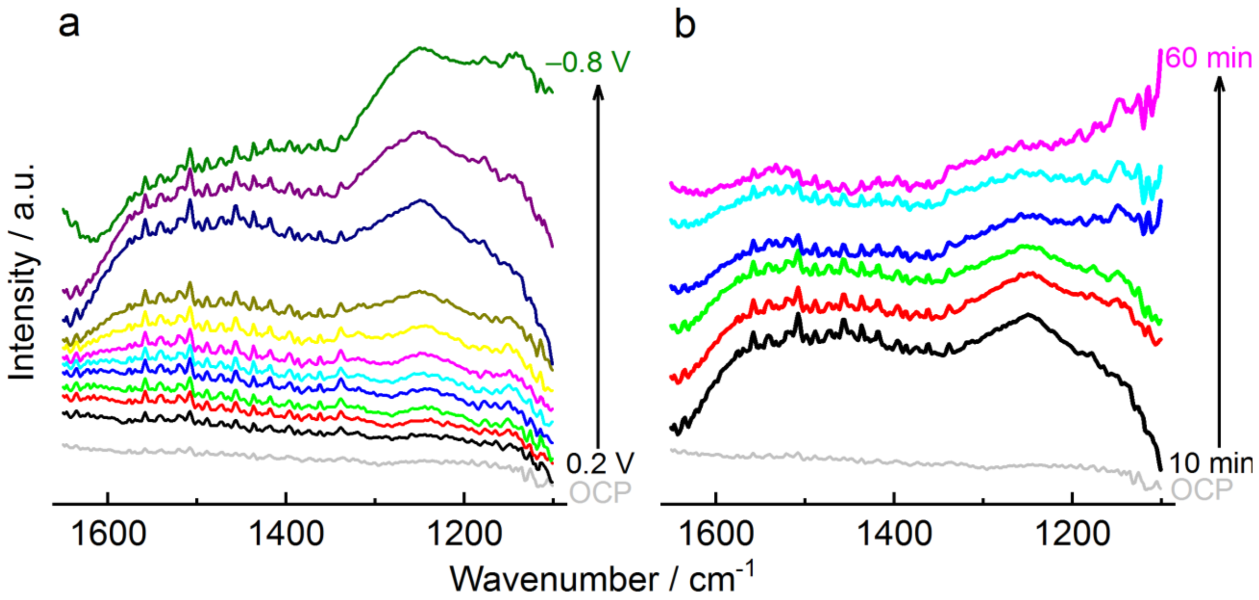

Figure S45. a) In-situ FTIR spectra at different chronoamperometric potentials (difference of 100 mV for each spectrum from 0.2 to –0.8 V vs RHE) and the open circuit potential (OCP) spectrum and b) in-situ FTIR spectra in different times (after 10 min of chronoamperometry at –0.6 V vs RHE for the acquisition of each spectrum) and the OCP spectrum. The Co_3O_4(Cowt.%55)GNR catalyst (37.5 µg cm^{-2}) supported on GC was used as working electrode in the presence of 0.1 M K_2SO_4.

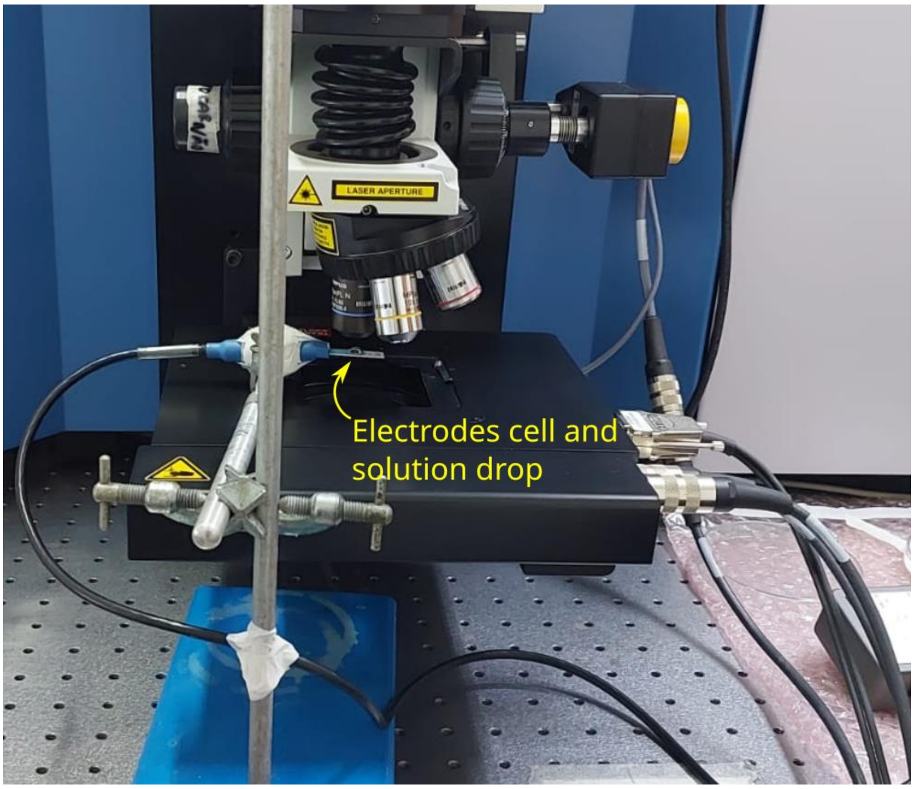

Figure S46. Image of the in-situ Raman equipment and electrodes cell with modified carbon working electrode, Ag reference electrode, and carbon counter electrode (CE) coverage with a drop solution.

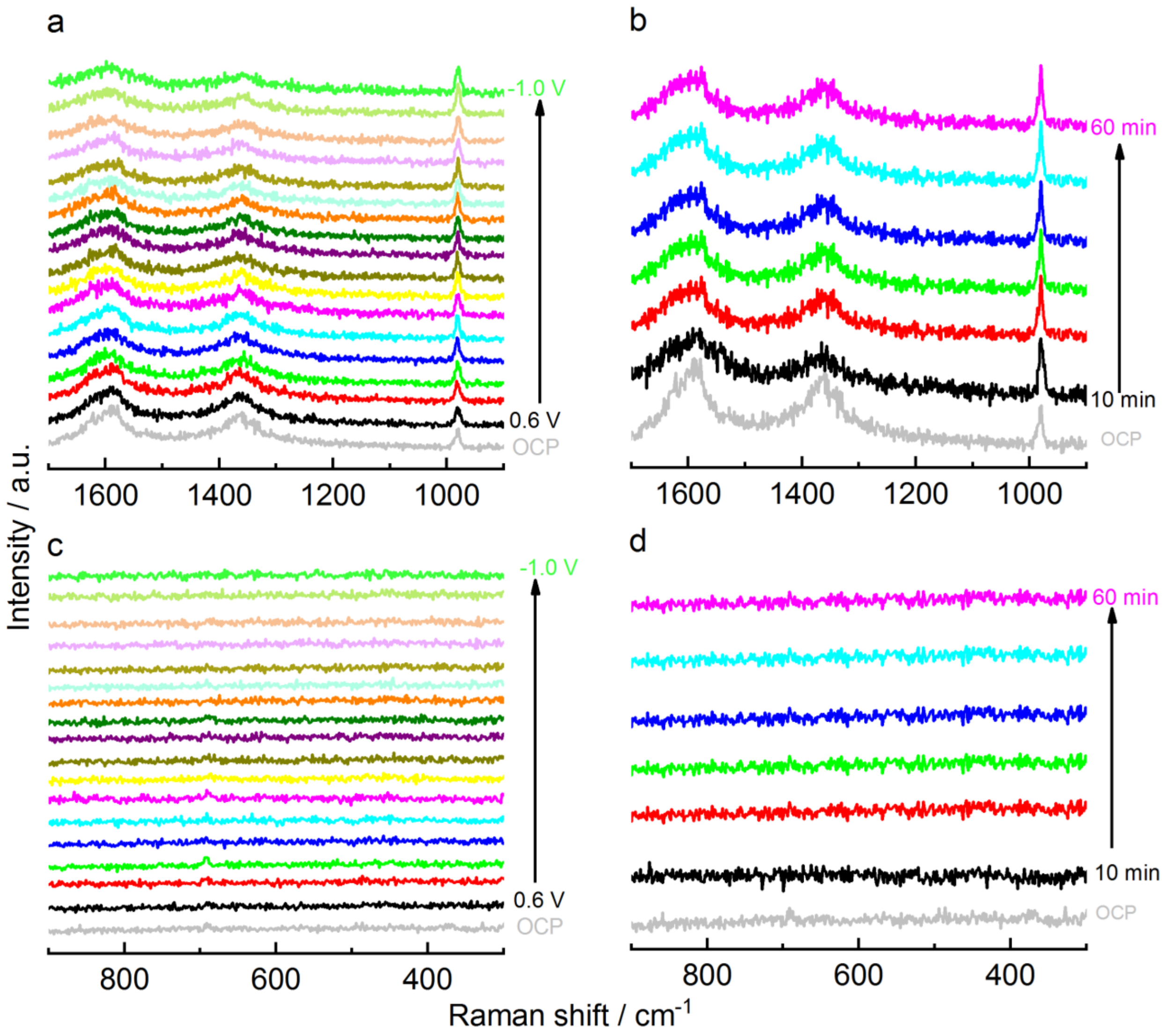

Figure S47. a and c) In-situ Raman spectra at different chronoamperometric potentials (difference of 100 mV for each spectrum from 0.6 to –1.0 V vs RHE) and the OCP spectrum, and b and d) in-situ Raman spectra in different times (after 10 min of chronoamperometry at –0.6 V vs RHE for the acquisition of each spectrum) and the OCP spectrum. The Co_3O_4(Cowt.%55)GNR catalyst (37.5 µg cm^{-2}) supported on carbon was used as working electrode in the presence of 0.1 M K_2SO_4.

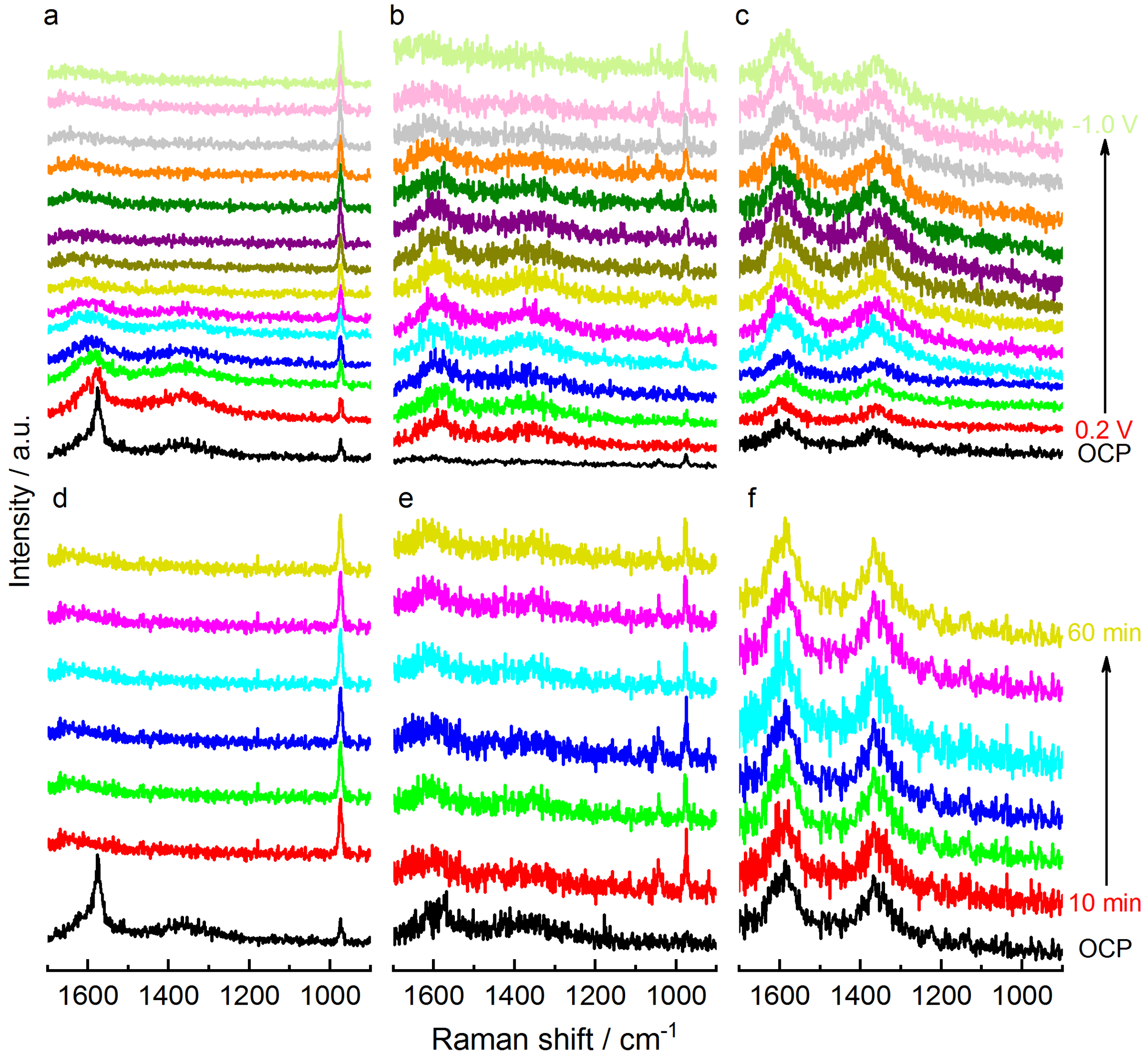

Figure S48. In-situ Raman spectra at different chronoamperometric potentials (difference of 100 mV for each spectrum from 0.6 to –1.0 V vs RHE) and the OCP spectrum for the bare Co_3O_4(Cowt.%75) catalyst (37.5 µg cm^{-2}) supported on carbon used as working electrode, in the presence of 0.1 M K_2SO_4 (a) and 40 mM $NaNO_3$ and 0.1 M K_2SO_4 (b), and for the bare GNR(Cowt.%0) catalyst (37.5 µg cm^{-2}) supported on carbon used as working electrode, in the presence of 40 mM $NaNO_3$ and 0.1 M K_2SO_4 (c). In-situ Raman spectra in different times (after 10 min of chronoamperometry at –0.6 V vs RHE for the acquisition of each spectrum) and the OCP spectrum for the bare Co_3O_4(Cowt.%75) catalyst (37.5 µg cm^{-2}) supported on carbon used as working electrode, in the presence of 0.1 M K_2SO_4 (d) and 40 mM $NaNO_3$ and 0.1 M K_2SO_4 (e), and for the bare GNR(Cowt.%0) catalyst (37.5 µg cm^{-2}) supported on carbon used as working electrode, in the presence of 40 mM $NaNO_3$ and 0.1 M K_2SO_4 (f).

The NH_3 stepwise reaction process used in the DFT calculations is outlined below:

(1) surf + $HNO_3 \rightarrow {}^*NO_3 + H^+ + e^-$

(2) $^*NO_3 + 2H^+ + 2e^- \rightarrow {}^*NO_2 + H_2O(l)$

(3) $^*NO_2 + 2H^+ + 2e^- \rightarrow {}^*NO + H_2O\ (l)$

(4) $^*NO + H^+ + e^- \rightarrow {}^*NHO$

(5) $^*NHO + H^+ + e^- \rightarrow {}^*NH_2O$

(6) $^*NH_2O + H^+ + e^- \rightarrow {}^*NH_2OH$

(7) $^*NH_2OH + H^+ + e^- \rightarrow {}^*NH_2 + H_2O\ (l)$

(8) $^*NH_2 + H^+ + e^- \rightarrow$ surf + NH_3

Here, "surf" represents the adsorption site on the surface, while $^*NO_3$, $^*NO_2$, $^*NO$, $^*NHO$, $^*NH_2O$, $^*NH_2OH$, and $^*NH_2$, and NH_3 refer to the adsorbed species.

The Gibbs free energy changes (ΔG) for each reaction step were calculated using the following expressions:

$\Delta G_1 = E_{*NO3} - E_{surf} - E_{HNO3} + 1/2E_{H2} + (\Delta ZPE - T\Delta S)_1 + \Delta G_{pH} + \Delta G_U$

$\Delta G_2 = E_{*NO2} + E_{H2O} - E_{*NO3} - E_{H2} + (\Delta ZPE - T\Delta S)_2 + \Delta G_{pH} + \Delta G_U$

$\Delta G_3 = E_{*NO} + E_{H2O} - E_{*NO2} - E_{H2} + (\Delta ZPE - T\Delta S)_3 + \Delta G_{pH} + \Delta G_U$

$\Delta G_4 = E_{*NHO} - E_{*NO} - 1/2E_{H2} + (\Delta ZPE - T\Delta S)_4 + \Delta G_{pH} + \Delta G_U$

$\Delta G_5 = E_{*NH2O} - E_{*NHO} - 1/2E_{H2} + (\Delta ZPE - T\Delta S)_5 + \Delta G_{pH} + \Delta G_U$

$\Delta G_6 = E_{*NH2OH} - E_{*NH2O} - 1/2E_{H2} + (\Delta ZPE - T\Delta S)_6 + \Delta G_{pH} + \Delta G_U$

$\Delta G_7 = E_{*NH2} + E_{H2O} - E_{*NH2OH} - 1/2E_{H2} + (\Delta ZPE - T\Delta S)_7 + \Delta G_{pH} + \Delta G_U$

$\Delta G_8 = E_{NH3} + E_{surf} - E_{*NH2} - 1/2E_{H2} + (\Delta ZPE - T\Delta S)_8 + \Delta G_{pH} + \Delta G_U$

where:

- E represents the interaction energy obtained directly from the DFT calculations.
- ΔZPE and TΔS are the changes in zero-point energy and the entropic term, respectively, with values taken from references [30] and [31].

- ΔG_{pH} accounts for the contribution of H^+ concentration to the free energy and is given by:

$$\Delta G_{pH} = - k_B T \ln[H^+] = pH\ k_B T \ln 10$$

where k_B is the Boltzmann constant, with T = 298.15 K and pH = 9 used in the present study.

- ΔG_U represents the energy shift at a specific electrode potential for all states involving electron transfer, calculated as −eU. The reference potential is set to that of the standard hydrogen electrode (SHE), and the chemical potential of H^+ + e^- is related to ½H_2 in the gas phase under standard conditions (pH = 0, p = 1 bar, T = 298 K, U = 0).

References

(1) Cardoso, E. S. F.; Fortunato, G. V.; Rodrigues, C. D.; Júnior, F. E. B.; Ledendecker, M.; Lanza, M. R. V.; Maia, G. Impacts of Graphene Nanoribbon Production Methods on Oxygen-Reduction Electrocatalysis in Different Environments. *ChemElectroChem* **2024**, *11* (4), e202300505. https://doi.org/10.1002/celc.202300505.

(2) Cardoso, E. S. F.; Fortunato, G. V.; Rodrigues, C. D.; Lanza, M. R. V.; Maia, G. Exploring the Potential of Heteroatom-Doped Graphene Nanoribbons as a Catalyst for Oxygen Reduction. *Nanomaterials* **2023**, *13* (21), 2831. https://doi.org/10.3390/nano13212831.

(3) Martini, B. K.; Maia, G. Using a Combination of Co, Mo, and Pt Oxides along with Graphene Nanoribbon and MoSe2 as Efficient Catalysts for OER and HER. *Electrochim. Acta* **2021**, *391*, 138907. https://doi.org/10.1016/j.electacta.2021.138907.

(4) Cardoso, E. S. F.; Fortunato, G. V.; Maia, G. Use of Rotating Ring-Disk Electrodes to Investigate Graphene Nanoribbon Loadings for the Oxygen Reduction Reaction in Alkaline Medium. *ChemElectroChem* **2018**, *5* (13), 1691–1701. https://doi.org/10.1002/celc.201800331.

(5) Rodrigues, C. D.; Bezerra, L. S.; Cardoso, E. S. F.; Fortunato, G. V.; Boruah, P. K.; Das, M. R.; Lanza, M. R. V.; Maia, G. Using Coupled Ni and Zn Oxides Based on ZIF8 as Efficient Electrocatalyst for OER. *Electrochim. Acta* **2022**, *435*, 141362. https://doi.org/10.1016/j.electacta.2022.141362.

(6) Wang, H.; Mao, Q.; Ren, T.; Zhou, T.; Deng, K.; Wang, Z.; Li, X.; Xu, Y.; Wang, L. Synergism of Interfaces and Defects: Cu/Oxygen Vacancy-Rich Cu-Mn 3 O 4 Heterostructured Ultrathin Nanosheet Arrays for Selective Nitrate Electroreduction to Ammonia. *ACS Appl. Mater. Interfaces* **2021**, *13* (37), 44733–44741. https://doi.org/10.1021/acsami.1c11249.

(7) Li, L.-X.; Sun, W.-J.; Zhang, H.-Y.; Wei, J.-L.; Wang, S.-X.; He, J.-H.; Li, N.-J.; Xu, Q.-F.; Chen, D.-Y.; Li, H.; Lu, J.-M. Highly Efficient and Selective Nitrate Electroreduction to Ammonia Catalyzed by Molecular Copper Catalyst@Ti 3 C 2 T x MXene. *J. Mater. Chem. A* **2021**, *9* (38), 21771–21778.

https://doi.org/10.1039/D1TA06664A.

(8) Weatherburn, M. W. Phenol-Hypochlorite Reaction for Determination of Ammonia. *Anal. Chem.* **1967**, *39* (8), 971–974. https://doi.org/10.1021/ac60252a045.

(9) Watt, G. W.; Chrisp, J. D. Spectrophotometric Method for Determination of Hydrazine. *Anal. Chem.* **1952**, *24* (12), 2006–2008. https://doi.org/10.1021/ac60072a044.

(10) Deng, X.; Yang, Y.; Wang, L.; Fu, X.; Luo, J. Metallic Co Nanoarray Catalyzes Selective NH 3 Production from Electrochemical Nitrate Reduction at Current Densities Exceeding 2 A Cm −2. *Adv. Sci.* **2021**, *8* (7), 2004523. https://doi.org/10.1002/advs.202004523.

(11) Deng, Z.; Liang, J.; Liu, Q.; Ma, C.; Xie, L.; Yue, L.; Ren, Y.; Li, T.; Luo, Y.; Li, N.; Tang, B.; Ali Alshehri, A.; Shakir, I.; Agboola, P. O.; Yan, S.; Zheng, B.; Du, J.; Kong, Q.; Sun, X. High-Efficiency Ammonia Electrosynthesis on Self-Supported Co2AlO4 Nanoarray in Neutral Media by Selective Reduction of Nitrate. *Chem. Eng. J.* **2022**, *435*, 135104. https://doi.org/10.1016/j.cej.2022.135104.

(12) He, W.; Zhang, J.; Dieckhöfer, S.; Varhade, S.; Brix, A. C.; Lielpetere, A.; Seisel, S.; Junqueira, J. R. C.; Schuhmann, W. Splicing the Active Phases of Copper/Cobalt-Based Catalysts Achieves High-Rate Tandem Electroreduction of Nitrate to Ammonia. *Nat. Commun.* **2022**, *13* (1), 1129. https://doi.org/10.1038/s41467-022-28728-4.

(13) Wang, J.; Cai, C.; Wang, Y.; Yang, X.; Wu, D.; Zhu, Y.; Li, M.; Gu, M.; Shao, M. Electrocatalytic Reduction of Nitrate to Ammonia on Low-Cost Ultrathin CoO x Nanosheets. *ACS Catal.* **2021**, *11* (24), 15135–15140. https://doi.org/10.1021/acscatal.1c03918.

(14) Yu, Y.; Wang, C.; Yu, Y.; Wang, Y.; Zhang, B. Promoting Selective Electroreduction of Nitrates to Ammonia over Electron-Deficient Co Modulated by Rectifying Schottky Contacts. *Sci. China Chem.* **2020**, *63* (10), 1469–1476. https://doi.org/10.1007/s11426-020-9795-x.

(15) Li, Z.; Wen, G.; Liang, J.; Li, T.; Luo, Y.; Kong, Q.; Shi, X.; Asiri, A. M.; Liu, Q.; Sun, X. High-Efficiency Nitrate Electroreduction to Ammonia on Electrodeposited Cobalt–Phosphorus Alloy Film. *Chem. Commun.* **2021**, *57* (76), 9720–9723. https://doi.org/10.1039/D1CC02612G.

(16) Gao, J.; Jiang, B.; Ni, C.; Qi, Y.; Zhang, Y.; Oturan, N.; Oturan, M. A. Non-Precious Co3O4-TiO2/Ti Cathode Based Electrocatalytic Nitrate Reduction: Preparation, Performance and Mechanism. *Appl. Catal. B Environ.* **2019**, *254*, 391–402. https://doi.org/10.1016/j.apcatb.2019.05.016.

(17) Jia, Y.; Ji, Y.-G.; Xue, Q.; Li, F.-M.; Zhao, G.-T.; Jin, P.-J.; Li, S.-N.; Chen, Y. Efficient Nitrate-to-Ammonia Electroreduction at Cobalt Phosphide Nanoshuttles. *ACS Appl. Mater. Interfaces* **2021**, *13* (38), 45521–45527. https://doi.org/10.1021/acsami.1c12512.

(18) Cerrón-Calle, G. A.; Wines, A.; Garcia-Segura, S. Atomic Hydrogen Provision by Cobalt Sites in a Bimetallic Ni/Co(OH)x and Trimetallic Ni/Cu2O/Co(OH)x Configurations for Superior Ammonia Production. *Appl. Catal. B Environ.* **2023**, *328*, 122540. https://doi.org/10.1016/j.apcatb.2023.122540.

(19) Xu, S.; Shi, Y.; Wen, Z.; Liu, X.; Zhu, Y.; Liu, G.; Gao, H.; Sun, L.; Li, F. Polystyrene Spheres-Templated Mesoporous Carbonous Frameworks Implanted with Cobalt Nanoparticles for Highly Efficient Electrochemical Nitrate Reduction to Ammonia. *Appl. Catal. B Environ.* **2023**, *323*, 122192. https://doi.org/10.1016/j.apcatb.2022.122192.

(20) Fu, J.; Yao, F.; Xie, T.; Zhong, Y.; Tao, Z.; Chen, S.; He, L.; Pi, Z.; Hou, K.; Wang,

D.; Li, X.; Yang, Q. In-Situ Growth of Needle-like Co3O4 on Cobalt Foam as a Self-Supported Cathode for Electrochemical Reduction of Nitrate. *Sep. Purif. Technol.* **2021**, *276*, 119329. https://doi.org/10.1016/j.seppur.2021.119329.

(21) Fu, W.; Du, X.; Su, P.; Zhang, Q.; Zhou, M. Synergistic Effect of Co(III) and Co(II) in a 3D Structured Co 3 O 4 /Carbon Felt Electrode for Enhanced Electrochemical Nitrate Reduction Reaction. *ACS Appl. Mater. Interfaces* **2021**, *13* (24), 28348–28358. https://doi.org/10.1021/acsami.1c07063.

(22) Niu, Z.; Fan, S.; Li, X.; Liu, Z.; Wang, J.; Duan, J.; Tadé, M. O.; Liu, S. Facile Tailoring of the Electronic Structure and the D-Band Center of Copper-Doped Cobaltate for Efficient Nitrate Electrochemical Hydrogenation. *ACS Appl. Mater. Interfaces* **2022**, *14* (31), 35477–35484. https://doi.org/10.1021/acsami.2c04789.

(23) Deng, Z.; Ma, C.; Li, Z.; Luo, Y.; Zhang, L.; Sun, S.; Liu, Q.; Du, J.; Lu, Q.; Zheng, B.; Sun, X. High-Efficiency Electrochemical Nitrate Reduction to Ammonia on a Co 3 O 4 Nanoarray Catalyst with Cobalt Vacancies. *ACS Appl. Mater. Interfaces* **2022**, *14* (41), 46595–46602. https://doi.org/10.1021/acsami.2c12772.

(24) Hu, Q.; Qi, S.; Huo, Q.; Zhao, Y.; Sun, J.; Chen, X.; Lv, M.; Zhou, W.; Feng, C.; Chai, X.; Yang, H.; He, C. Designing Efficient Nitrate Reduction Electrocatalysts by Identifying and Optimizing Active Sites of Co-Based Spinels. *J. Am. Chem. Soc.* **2024**, *146*, 2967−2976. https://doi.org/10.1021/jacs.3c06904.

(25) Niu, Z.; Fan, S.; Li, X.; Yang, J.; Wang, J.; Tao, Y.; Chen, G. Tailored Electronic Structure by Sulfur Filling Oxygen Vacancies Boosts Electrocatalytic Nitrogen Oxyanions Reduction to Ammonia. *Chem. Eng. J.* **2023**, *451*, 138890. https://doi.org/10.1016/j.cej.2022.138890.

(26) Meng, Z.; Yao, J.; Sun, C.; Kang, X.; Gao, R.; Li, H.; Bi, B.; Zhu, Y.; Yan, J.; Jiang, Q. Efficient Ammonia Production Beginning from Enhanced Air Activation. *Adv. Energy Mater.* **2022**, *12* (38), 2202105 (1 to 9). https://doi.org/10.1002/aenm.202202105.

(27) Zhang, J.; He, W.; Quast, T.; Junqueira, J. R. C.; Saddeler, S.; Schulz, S.; Schuhmann, W. Single-entity Electrochemistry Unveils Dynamic Transformation during Tandem Catalysis of Cu 2 O and Co 3 O 4 for Converting NO 3 − to NH 3. *Angew. Chemie Int. Ed.* **2023**, *62* (8), e202214830 (1 to 8). https://doi.org/10.1002/anie.202214830.

(28) Qiao, L.; Liu, D.; Zhu, A.; Feng, J.; Zhou, P.; Liu, C.; Ng, K. W.; Pan, H. Nickel-Facilitated in-Situ Surface Reconstruction on Spinel Co3O4 for Enhanced Electrochemical Nitrate Reduction to Ammonia. *Appl. Catal. B Environ.* **2024**, *340*, 123219. https://doi.org/10.1016/j.apcatb.2023.123219.

(29) Zhong, L.; Chen, Q.; Yin, H.; Chen, J. S.; Dong, K.; Sun, S.; Liu, J.; Xian, H.; Li, T. Co 3 O 4 Nanoparticles Embedded in Porous Carbon Nanofibers Enable Efficient Nitrate Reduction to Ammonia. *Chem. Commun.* **2023**, *59* (58), 8973–8976. https://doi.org/10.1039/D3CC02023A.

(30) Nørskov, J. K.; Rossmeisl, J.; Logadottir, A.; Lindqvist, L.; Kitchin, J. R.; Bligaard, T.; Jónsson, H. Origin of the Overpotential for Oxygen Reduction at a Fuel-Cell Cathode. *J. Phys. Chem. B* **2004**, *108* (46), 17886–17892. https://doi.org/10.1021/jp047349j.

(31) Atkins, P.; Paula, J. D. *Physical Chemistry: Thermodynamics, Structure, and Change*, 10th ed.; W. H. Freeman and Company: New York, 2014.
